# Supplementary material for: The FIP 1.0 Data Set: Highly resolved annotated image time series of 4,000 wheat plots grown in 6 years
Source: Gigascience. 2025 Jun 11;14:giaf051. doi: 10.1093/gigascience/giaf051 (PMC12153353; doi:10.1093/gigascience/giaf051)
Supplement: giaf051_GIGA-D-24-00504_Revision_1 [file giaf051_giga-d-24-00504_revision_1.pdf]

GigaScience

The FIP 1.0 Data Set: Highly Resolved Annotated Image Time Series of 4,000 Wheat Plots Grown in Six Years

--Manuscript Draft--

Manuscript Number:	GIGA-D-24-00504R1
Full Title:	The FIP 1.0 Data Set: Highly Resolved Annotated Image Time Series of 4,000 Wheat Plots Grown in Six Years
Article Type:	Data Note
Funding Information:	Schweizerischer Nationalfonds zur Förderung der Wissenschaftlichen Forschung (169542) — Prof. Dr. Achim Walter<br>Schweizerischer Nationalfonds zur Förderung der Wissenschaftlichen Forschung (200756) — Prof. Dr. Achim Walter<br>Eidgenössische Technische Hochschule Zürich (C21-04) — Dr Lukas Roth
Abstract:	Background:<br>Understanding genotype-environment interactions of plants is crucial for crop improvement, yet limited by the scarcity of quality phenotyping data. This data note presents the Field Phenotyping Platform 1.0 data set, a comprehensive resource for winter wheat research that combines imaging, trait, environmental, and genetic data.<br><br>Findings: We provide time series data for more than 4,000 wheat plots, including aligned high-resolution image sequences totaling more than 153,000 aligned images across six years. Measurement data for eight key wheat traits is included, namely canopy cover values, plant heights, wheat head counts, senescence ratings, heading date, final plant height, grain yield, and protein content. Genetic marker information and environmental data complement the time series. Data quality is demonstrated through heritability analyses and genomic prediction models, achieving accuracies aligned with previous research.<br><br>Conclusions: This extensive data set offers opportunities for advancing crop modeling and phenotyping techniques, enabling researchers to develop novel approaches for understanding genotype-environment interactions, analyzing growth dynamics, and predicting crop performance. By making this resource publicly available, we aim to accelerate research in climate-adaptive agriculture and foster collaboration between plant science and machine learning communities.
Corresponding Author:	Norbert Kirchgessner<br>ETH Zurich D-USYS: Eidgenossische Technische Hochschule Departement Umweltsystemwissenschaften<br>Zürich, SWITZERLAND
Corresponding Author Secondary Information:	
Corresponding Author's Institution:	ETH Zurich D-USYS: Eidgenossische Technische Hochschule Departement Umweltsystemwissenschaften
Corresponding Author's Secondary Institution:	
First Author:	Lukas Roth
First Author Secondary Information:	
Order of Authors:	Lukas Roth<br>Mike Boss, MSc

Powered by Editorial Manager® and ProduXion Manager® from Aries Systems Corporation

	Norbert Kirchgessner
	Helge Aasen
	Brenda Patricia Aguirre-Cuellar
	Price Pius Atuah Akiina
	Jonas Anderegg
	Joaquin Gajardo Castillo
	Xiaoran Chen
	Simon Corrado
	Krzysztof Cybulski
	Beat Keller
	Stefan Kortstee
	Lukas Kronenberg
	Frank Liebisch
	Paraskevi Nousi
	Corina Oppliger
	Gregor Perich
	Johannes Pfeifer
	Kang Yu
	Nicola Storni
	Flavian Tschurr
	Michele Volpi
	Simon Treier
	Hansueli Zellweger
	Olivia Zumsteg
	Andreas Hund
	Achim Walter
**Order of Authors Secondary Information:**	
**Response to Reviewers:**	Dear editors of GigaScience,<br><br>Thank you for giving us the chance to perform major revisions on our data note manuscript based on the comments of two reviewers. In the last weeks, we have invested a collaborative effort into revising our manuscript as well as providing additional data and analyses to further improve its value for the community. In particular, we have reacted to the major concerns of reviewer #1 and have<br>clearly stated the availability of open genetic data for the majority of the presented data and the ability to have full access to all genetic data via collaborations under clearly defined conditions<br>addressed the MIAPPE standard and are now fully compatible with MIAPPE v1.1<br><br>In reaction to the concerns of reviewer #2 regarding quality control and consistency checks, we have substantially revised our manuscript to clarify open points.<br><br>Please find our point-by-point reply to all raised questions and issues by the reviewers as well as the revised manuscript and the manuscript with highlighted changes below.

Powered by Editorial Manager® and ProduXion Manager® from Aries Systems Corporation

Reviewer #1: Thank you for the submission. The dataset surely holds value for the plant breeding community

Thank you very much for your thorough review. We have substantially revised the manuscript to address your concerns. Please find indications of all revisions and reactions to your comments below.

but my major concerns are
(1) the availability of genetic data,

We agree that the dependence on non-public genetic data would restrict the value of the dataset. Yet, this is not the case: For the majority of the data presented in this data note, marker data are public. This data set allows all the uses described in the manuscript, and we used it as default for the baselines, splits, etc. (see e.g. Figure 5). Yet, we wanted to inform the reader of the data note that it is possible to extend the public dataset via collaborating with the Swiss plant breeders, which may be an interesting option for some, but absolutely no prerequisite. We have reformulated the section in the introduction to point this out more clearly:

"For the first three years (2016-2018), the GABI-WHEAT [13] panel was grown as the genotype set. From 2019-2022, a subset of the GABI panel was grown in addition to other genotypes (Figure 2, green bars). The GABI panel consists of registered genotypes from different climatic regions of Europe [14, 13]. Genetic marker data and MET data from eight year-locations for GABI-WHEAT are publicly available.

The GABI panel was largely superseded by the Swiss breeding set in 2021 (Figure 2, orange bars). This new set primarily consists of eighth-generation (F8) breeding genotypes.
Private genetic marker data are available for the Swiss breeding set. These can be shared directly through the breeders as part of a collaboration. The remaining genotypes, linked to specific projects such as INVITE, were present throughout all years but were generally only grown in a single year each (Figure 2, in purple). These genotypes currently lack available marker data.

In summary, by default the dataset contains public genotypic data from ~300 genotypes over six years, allowing all the uses described in this dataset note. Through collaborations, this set can be expanded to ~800 genotypes."

(2) non-conformity to MIAPPE standards (https://www.miappe.org/).

We agree that MIAPPE is an important logical data model to ensure a minimum set of metadata describing phenotyping experiments. We have used MIAPPE as reference and checklist for the design of our own internal database that holds our experimental data and metadata (https://kp.ethz.ch/infrastructure/cropydb.html). This approach corresponds to the aims of MIAPPE (https://www.miappe.org/: "MIAPPE provides a specification including a checklist and a data model of metadata required to adequately describe plant phenotyping experiments.")

Consequently, we consider the exports of the database in the shared data repositories accompanying this data note as fully conform to MIAPPE standards.

To further facilitate the re-use of our MIAPPE-compatible dataset, we have now added the necessary MIAPPE tsv files to both the research collection and Huggingface dataset repository. In addition, we also added the script to generate the MIAPPE files for the biological material, observation unit and observed variables to the fip1_dataset repository under miappe.py.

We have added a corresponding hint to the "Interoperable" declaration:
"Interoperable: The use of the open-source Hugging Face datasets [27] package makes it easy to use and export to different formats and is fully MIAPPE v1.1 [28] conform."

These restrict value of the otherwise excellent publication. I would welcome a

Powered by Editorial Manager® and ProduXion Manager® from Aries Systems Corporation

submission addressing these major points. In addition, I have some minor points for specific sections. Please use the strings in quotation marks ("") to locate the specific sections.
1. Context
* Change of Equipment: Please indicate how the change of equipment from TLS to drone affects data interoperability.

We have indeed demonstrated in previous publications that those two measurements are in high accordance with each other. We have revised the sentence accordingly:

“Up to three times a week, RGB images of all experimental units (so-called `plots') were collected, and plant heights were measured simultaneously using either the TLS (2016, 2017) [8, 9] or drones (2018--2022) [10, 11, 12], two methods of height measurement that have been in good accordance with one another (R2: 0.99 [12]).”

Furthermore, we added supplementary information to the materials and methods section:

“The accuracy of TLS and drone measurements to approximate manual plant height measurements were demonstrated in the respective publications (TLS [8]: R2 : 0.99, drone [37]: R2 : 0.96). The two methods are in good accordance with one another (R2 : 0.99, intercept: 0.057 m, slope: 1.0 [12]).”

* "Figure 2, gray bars": Kindly update Figure 2 to clarify the representation of the gray bars.

Sorry for that oversights in the text, we confused “grey bar” with “orange bar”:

“The GABI-WHEAT panel was largely superseded by the Swiss breeding set in 2021 (Figure 2, orange bars)”

* "Heads were annotated": Does this mean that not all relevant images were annotated? If so, please modify the title to avoid confusion.

Image annotation may be done on different levels, from labels of images to bounding boxes or polygons. All of these data are considered “annotations”. The time series in this data set are annotated with multiple labels per image (canopy cover, number of wheat heads, plant height, senescence) and multiple labels per time series (yield, protein content, heading date, final height). Hence, we have chosen the title “highly resolved annotated image time series”, which we found to adequately describe the characteristics of the dataset.

To point out not only the potential but also the limitations of the data set, we have added a discussion to the section “Re-use Potential” and renamed it to “Re-use Potential and Limitations”:

“While the data set opens up the possibility of analysing HTFP data to a wide audience, it also has its inherent limitations that should be taken into account if working with it:
The immobility of the FIP restricts the data set to only one location.
Field-based data collection introduces various sources of errors that one must consider in analysis (see e.g. [20] for a discussion).
Annotation at the image level only requires further annotation effort if semantic segmentation or object detection methods are targeted.”

If you do not agree with this argumentation and still have concerns about confusion on the readers side, we are of course open for suggestions on how to more accurately describe this type of annotations in the title.

* Description of FAIR: Please revise this section. Both links listed under "Findable" and "Accessible" are eligible for these tags. Please modify "Interoperability" with reference to the publication listed in the "Re-use Potential."

Powered by Editorial Manager® and ProduXion Manager® from Aries Systems Corporation

We have added the most important publications from the re-use potential to the interoperability section.

“Given the shared genotypes the data set can be used to enhance the data by Gogna et al. [13] by 6 environments, to a total of 14. The data set expands on existing sub-sets of the data already released that can be used as baseline approaches such as [8, 9, 19, 20, 21, 12, 25, 15, 16, 17].”

2. Reference measurements
* "Senescence was": Was this measurement done for all relevant images? Please include this information.

The period and years of measurement were indeed missing for the senescence ratings, thank you for pointing this out. We revised the sentence:

“Senescence was assessed visually in 2016, 2017 and 2018 from approximately 20 days after flowering to full senescence for the central plot area canopy, following guidelines provided by Pask et al. [39]. Plot senescence was scored according to Anderegg et al. [16] based on the portion of green leaf area on a scale from 0 to 10, equivalent to 0 to 100 %.”

* "Adjusted genotype means with year calculation": Please add variance decomposition data for traits.

Thank you for this suggestion. We extracted the estimated variance components for the intermediate and target traits and displayed the results in a new table, Table 4. When rerunning the mixed models, we took the opportunity to further increase the consistency with the low-level trait BLUEs and heritability estimates that were performed in SpATS, and changed estimations for intermediate and target traits also to SpATS. Heritabilities changed slightly, but not significantly (Table 3).

When doing the recalculations, we realized that the spatial variances for yield for 2019 were exceptionally high in comparison to other years. Further investigations revealed that not all plots in 2019 had the same size: While some were 1 m x 1.5 m size as all plots of all other years, 144 plots had size 5 m x 1.5 (yield plots). The yield values for those yield plots were on average 1.5 times lower than the ones for the small plots, despite being normalized to area. Hence, we concluded that the small plot size has led to a systematic overestimation of yield, which is not relevant if staying in sets with the same plot size, but becomes relevant for 2019 where plot sizes are heterogeneous.

We have added corresponding parts to the manuscript that explain how the conversion factor between the two sizes was evaluated using common genotypes, and how yield for small plot sizes was compensated afterwards, leading to a new trait “Grain yield (adjusted)”. We also have re-run all baseline approaches (genomic predictions) and further clarified the description of the linear mixed models used and fall-back versions in case of failed convergence.

Changes:
Table 4: Variance decomposition results
Table 3: Heritability for Grain yield (adjusted) 2019 added
Table 7: Genomic prediction results for Grain yield (adjusted) added
Materials and Methods, Adjusted genotype means within year calculation: Correction of definition of fixed and random factors, fall-back options for failed convergence introduced
Materials and Methods, Target Traits: Description added how yield for small plot sizes was compensated:

“In 2019, plot sizes varied between experiments, which influences yield measurements.
While large plot sizes deliver absolute estimates comparable with METs, in our experience, small plot sizes tend to overestimate yield per area. To compensate for this effect, adjusted genotype means (see next Section) of twelve common genotypes between the experiments with large and small plots were used to calculate a conversion factor. A linear regression with intercept zero estimated a conversion factor

Powered by Editorial Manager® and ProduXion Manager® from Aries Systems Corporation

of 1.50 from large to small plots (R2 of 0.47). This conversion factor was used to transform all yield measurement estimates of small plots to the range of large plots, resulting in a new trait `Grain yield (adjusted)'."

3. Compilation as Data set
* "pure GABI-WHEAT set for the extended set": Please revise this sentence for clarity.

We have corrected and clarified the sentences:
"The splits were calculated separately for the pure GABI-WHEAT set and for the extended set. The extended set additionally includes F8 generation genotypes with private marker data."

4. Heritabilities of intermediate and target traits
* "y of the public marker" - Please revise the sentence for clarity.

We have corrected the sentence:

"The quality of the public marker data set for the GABI-WHEAT panel was demonstrated in Gogna et al. by means of testing for genomic prediction ability [13]."

5. Genomic prediction ability of unseen multi-environment trial
* Is the CDC data part of the data publication? Please add this information.

We have clarified this point:

"These additional environmental covariate data and MET data are available in the data repository for convenience, but not part of the core data set (see folder `MET_repository_clone')."

6. Example 1 to 6
* Please revise all code for consistency and updated results. Also, include the necessary packages required to run the code.

Given the changes to the dataset we adjusted the code examples and added a clarifying sentence that only the datasets package is required.

To run the following examples the Huggingface datasets [27] library is required. The examples were run using version 3.3.2.

7. Availability of Source code and Requirement Please create connectivity between repositories and add descriptive README files outlining their usage. Additionally, please provide instructions on how individual repositories may be used.

We have updated the READMEs of the three repositories as well as added explanatory text to the "Availability of Source Code and Requirements" section:

"The code to recreate the derived data and the dataset is publicly available in three repositories, namely the FIP 1.0 Data Set - Traits, fip1-alignment, and fip1-dataset repositories.

The complete process to create the dataset involves extracting trait data from the raw data using the FIP 1.0 Data Set - Traits repository, then aligning the image time-series using the fip1-alignment repository, and finally aggregating the derived data into the final dataset using the fip1-dataset repository.

In addition, the dataset can be recreated using the fip1-dataset repository from the derived data that is freely available in the ETH research collection."

I appreciate your attention to these points and believe that addressing them will strengthen your manuscript.

Reviewer #2: The manuscript presents a comprehensive dataset spanning six years, encompassing data from eight key growth stages of wheat, along with corresponding phenotypic data. The construction of such a comprehensive dataset is highly valuable. However, from the perspective of dataset construction itself, quality control and consistency checks require further refinement.

Thank you very much for your thorough review. We have substantially revised the manuscript to address your concerns. Please find indications of all revisions and reactions to your comments below.

Specific issues are as follows:
1. How is the consistency check of parameters such as canopy cover and plant height at the eight key growth stages ensured? Especially for parameters like phenological stages and senescence assessment, which are determined through visual evaluation and thus susceptible to subjective influences, quality control and consistency check become particularly crucial. It is recommended to supplement relevant content for detailed explanation.

We have added supplementary information to the quality of all measured and extracted low-level and intermediate traits:

Plant height:
"The accuracy of TLS and drone measurements to approximate manual plant height measurements were demonstrated in the respective publications (TLS [8]: R2 : 0.99, drone [37]: R2 : 0.96). The two methods are in good accordance with one another (R2 : 0.99, intercept: 0.057 m, slope: 1.0 [12])."

Canopy cover:
"From images, canopy cover was extracted using a deep learning model described in Zenkl et al. [22], a model that reached a pixel accuracy of 0.945 on a FIP test set."

Wheat head count:
"As the wheat head detection method, the winning model of the global wheat head challenge [24] (https://github.com/ksnxr/GWC_solution) was used, a model that achieved an average domain accuracy of 0.7 on a test set that included FIP images."

Senescence:
"To avoid bias, manual ratings were performed by the same expert in all three years."

Heading date:
"To ensure consistent ratings over time, heading date ratings were started at approximately BBCH 55 and continue to BBCH 61, with two to three rating events per week."

Final height:
"Final height was extracted from TLS or SfM plant height measurements using the QMER method described in Roth et al. [20], which has a reported accuracy of close to 1.0 on simulated data."

2. For all images (151,150 out of 158,891 images), the success rate of alignment and within-field detection exceeded 95%. Does this mean that the final RGB sequence image dataset consists of 151,150 images?

Yes, this is the case if one uses the aligned sequences, but not if using unaligned images. We have revised this section and added a table that shows the number of successfully aligned images for each year. As we updated our the data set, numbers changed slightly.

"In total, the alignment and inner plot detection was successful for more than 95% of all images. The number of successfully aligned images per year can be seen in Table 2. Both the 160,772 original images and their corresponding 153,022 inner plot cutouts

	are made<br>available as part of this data set."<br><br>3. Regarding plant height measurement, the text mentions that "TLS (2016, 2017) or UAV (2018 to 2022) was used to measure plant height." Given the potential differences in height measurements obtained from these two methods, how were these differences addressed in the manuscript?<br><br>We have indeed demonstrated in previous publications that those two measurements are in high accordance with each other. We have revised the sentence accordingly:<br><br>"Up to three times a week, RGB images of all experimental units (so-called `plots') were collected, and plant heights were measured simultaneously using either the TLS (2016, 2017) [8, 9] or drones (2018--2022) [10, 11, 12], two methods of height measurement that have been in good accordance with one another (R2: 0.99, Offset: 0.057 m [12])."<br><br>4. Does this dataset cater to different tasks and include annotated data? If so, it is recommended to specify the concrete annotation methods and data.<br><br>The data set does not include image annotations, e.g. segmentation masks. Only scalar values for all traits are included. We have added two clarifying sentences in the measurements section.<br><br>"Trait measurements may have been made at times different from the image captures. All measurements are scalars that correspond to a single measurement at a given time point during the season."<br><br>5. If possible, it is recommended to provide a summary table that specifies the different types of data contained in the dataset along with their respective quantities, facilitating readers' comprehensive understanding of the dataset.<br><br>We have added a table for the images / aligned images as well as an additional table for the low-level traits. Both these tables contain the minimum, maximum and mean number of measurements for the aligned images / trait for each year (Table 3 and 4).<br><br>6. What are the potential limitations of this dataset? It is recommended to point them out.<br><br>We have enhanced the "Re-use Potential" section to a "Re-use Potential and Limitations" section:<br><br>"While the data set opens up the possibility of analysing HTFP data to a wide audience, it also has its inherent limitations that should be taken into account if working with it:<br>The immobility of the FIP restricts the data set to only one location.<br>Field-based data collection introduces various sources of errors that one must consider in analysis (see e.g. [20] for a discussion).<br>Yield measurements in the FIP and hence this data set are more prone to error than in METs.<br>Annotation at the image level only requires further annotation effort if semantic segmentation or object detection methods are targeted."<br>While the aligned image time series provides extensive opportunities to analyze growth dynamics, this kind of highly preprocessed image data is yet rare and therefore interoperability with other data sources is limited.
**Additional Information:**	
**Question**	**Response**
Are you submitting this manuscript to a special series or article collection?	No
**Experimental design and statistics**	Yes

Powered by Editorial Manager® and ProduXion Manager® from Aries Systems Corporation

Full details of the experimental design and statistical methods used should be given in the Methods section, as detailed in our Minimum Standards Reporting Checklist. Information essential to interpreting the data presented should be made available in the figure legends.<br><br>Have you included all the information requested in your manuscript?	
**Resources**<br><br>A description of all resources used, including antibodies, cell lines, animals and software tools, with enough information to allow them to be uniquely identified, should be included in the Methods section. Authors are strongly encouraged to cite Research Resource Identifiers (RRIDs) for antibodies, model organisms and tools, where possible.<br><br>Have you included the information requested as detailed in our Minimum Standards Reporting Checklist?	Yes
**Availability of data and materials**<br><br>All datasets and code on which the conclusions of the paper rely must be either included in your submission or deposited in publicly available repositories (where available and ethically appropriate), referencing such data using a unique identifier in the references and in the "Availability of Data and Materials" section of your manuscript.<br><br>Have you have met the above requirement as detailed in our Minimum Standards Reporting Checklist?	Yes
GigaScience has policies and guidelines in place for the use of generative AI-	No

writing tools such as ChatGPT. If you have used such writing tools to assist with writing the manuscript this must be declared and cited in the text. Authors should not list AI-writing tools and other AI-assisted technologies as an author or co-author and should acknowledge that they are fully responsible for text generated or refined by AI-writing tools.<p><br><br>A summary of use (particularly in the introduction or among methods) needs to be included at the end of the paper, and the outputs should also be included as a supplementary file hosted in GigaDB or other open repositories. Please read our guidelines for more information. <p><br><br>By submitting to GigaScience, you are aware of the journal's AI-writing tools policy, and if you have declared use of such tools below, you have acknowledged this where appropriate in your manuscript and have made a summary of use and outputs available. <p><br><b>AI-assisted writing tools have been used in the preparation of this manuscript?	

Powered by Editorial Manager® and ProduXion Manager® from Aries Systems Corporation

Manuskript

Click here to access/download;Manuscript;FIP1.0_20250227.pdf

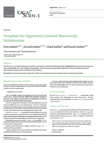

GigaScience, 0000, 1–16

doi: xx.xxxx/xxxx
Manuscript in Preparation
Data Note

DATA NOTE

The FIP 1.0 Data Set: Highly Resolved Annotated Image Time Series of 4,000 Wheat Plots Grown in Six Years

Lukas Roth[1,†], Mike Boss[1,†], Norbert Kirchgessner[1,†,*], Helge Aasen[1,a], Brenda Patricia Aguirre-Cuellar[1,b], Price Pius Atuah Akiina[1,c], Jonas Anderegg[1,d], Joaquin Gajardo Castillo[1], Xiaoran Chen[2], Simon Corrado[1], Krzysztof Cybulski[1,e], Beat Keller[1], Stefan Göbel Kortstee[1,f], Lukas Kronenberg[1,g], Frank Liebisch[1,h], Paraskevi Nousi[2], Corina Oppliger[1], Gregor Perich[1], Johannes Pfeifer[1,i], Kang Yu[1,k], Nicola Storni[1], Flavian Tschurr[1], Simon Treier[1], Michele Volpi[2], Hansueli Zellweger[1,l], Olivia Zumsteg[1], Andreas Hund[1] and Achim Walter[1]

[1]ETH Zürich, Institute of Agricultural Sciences, Zürich, Switzerland and [2]ETH Zürich and EPFL, Swiss Data Science Center, Switzerland and [a]Agroscope, Earth Observation of Agroecosystems Team, Zürich, Switzerland and [b]Universidad Nacional de Colombia, Facultad de Ciencias Agropecuarias, Bogotá, Colombia and [c]University of Wyoming, Department of Plant Sciences, Laramie, Wyoming, USA and [d]ETH Zurich, Plant Pathology Group, Zürich, Switzerland and [e]ETH Zurich, Seminar for Statistics, Zürich, Switzerland and [f]Sítio Florosa, São Paulo, Brazil and [g]The John Innes Centre, Crop Genetics, Norwich, United Kingdom and [h]Agroscope, Water Protection and Substance Flows Team, Zürich, Switzerland and [i]Federal Office for Agriculture and Food, Bonn, Germany and [k]Technical University of Munich, Precision Agriculture Lab, Freising, Germany and [l]Departement für Inneres und Volkswirtschaft (DIV), Landwirtschaftsamt, Thurgau, Switzerland

[*]corresponding author: Norbert Kirchgessner (norbert.kirchgessner@usys.ethz.ch)
[†]equal contribution
[‡] current affiliations listed as a–k

Abstract

Background: Understanding genotype-environment interactions of plants is crucial for crop improvement, yet limited by the scarcity of quality phenotyping data. This data note presents the Field Phenotyping Platform 1.0 data set, a comprehensive resource for winter wheat research that combines imaging, trait, environmental, and genetic data.
Findings: We provide time series data for more than 4,000 wheat plots, including aligned high-resolution image sequences totaling more than 153,000 aligned images across six years. Measurement data for eight key wheat traits is included, namely canopy cover values, plant heights, wheat head counts, senescence ratings, heading date, final plant height, grain yield, and protein content. Genetic marker information and environmental data complement the time series. Data quality is demonstrated through heritability analyses and genomic prediction models, achieving accuracies aligned with previous research.
Conclusions: This extensive data set offers opportunities for advancing crop modeling and phenotyping techniques, enabling researchers to develop novel approaches for understanding genotype-environment interactions, analyzing growth dynamics, and predicting crop performance. By making this resource publicly available, we aim to accelerate research in climate-adaptive agriculture and foster collaboration between plant science and machine learning communities.

Key words: Winter wheat; High-throughput phenotyping; Field phenotyping platform; Yield; Protein content; Image time series; Deep learning data set

Data Description

Aim

Winter wheat provides a crucial share of calories for human nutrition, with global demand steadily increasing [1]. However, crop production faces challenges due to limited resources like water, agrochemicals, and land [2]. Climate change further threatens crop yields, necessitating responsible and efficient resource use [3].

Crop yields are substantially driven by complex interactions between plant genetics and environmental factors. For instance, genes involved in fruit formation interact with temperatures at flowering, influencing growth and yield potential [4]. Limited phenotyping data is seen as the major reason for the incomplete understanding of such genotype-environment interactions [5].

High-throughput field phenotyping (HTFP) was developed to address this data gap [6]. Imaging HTFP platforms allow researchers to monitor crop canopy development over time, generating dense time series data of plant growth. There are many approaches to process such data ranging from extracting traits at critical time points to modeling growth dynamics and finally using end-to-end methods that directly analyze image time series.

This data set aims to provide a comprehensive foundation for these diverse approaches. Our goal is to foster collaboration between plant physiology, biometrics, and computer vision research, ultimately improving the ability to predict genotype-environment interactions for current and future climates.

Context

The Field Phenotyping Platform (FIP) at ETH was established in 2015 to collect image time series of crops growing under realistic field conditions. The FIP's cable carrying system is capable of carrying a 90 kg sensor head [7]. The original sensor head, hereafter referred to as the FIP 1.0 head, was equipped with a red, green, and blue (RGB) camera and a Terrestrial Laser Scanner (TLS), among other sensors. Wheat field experiments were observed using FIP 1.0 over an eight-year period from 2015 to 2022, yielding six years of data collection, with 2015 and 2020 excluded due to incomplete measuring seasons (Figure 1). RGB images of all experimental units (so-called 'plots') were collected up to three times a week, and plant heights were measured simultaneously using either the TLS (2016, 2017) [8, 9] or drones (2018–2022) [10, 11, 12], two methods of height measurement that have been in good accordance with one another (R^2: 0.99 [12]). In 2023, the FIP 1.0 sensor head was replaced with a new, multi-view RGB sensor head. The described data set includes all RGB and height data collected in winter wheat experiments up to this replacement.

The area of approximately one hectare that the FIP can monitor is divided into six smaller parts (so-called 'lots') that are integrated into a crop rotation. The two FIP lots dedicated to winter wheat provide space for ~350 genotypes, replicated once per lot. For the first three years (2016–2018), the GABI-WHEAT [13] panel was grown as the genotype set. From 2019–2022, a subset of the GABI-WHEAT panel was grown in addition to other genotypes (Figure 2, green bars). The GABI-WHEAT panel consists of registered genotypes from different climatic regions of Europe [14, 13]. Genetic marker data and Multi-Environment Trial (MET) data from eight year-locations for GABI-WHEAT are publicly available.

The GABI-WHEAT panel was largely superseded by the Swiss breeding set in 2021 (Figure 2, orange bars). This new set primarily consists of eighth-generation (F8) breeding genotypes. Private genetic marker data are available for the Swiss breeding set. These can be shared directly through the breeders as part of a collaboration. The remaining genotypes, linked to specific projects such as Innovation in Variety Testing (INVITE), were present throughout all years but were generally only grown in a single year each (Figure 2, purple bars). These genotypes currently lack available marker data.

In summary, by default the data set contains public genotypic data from ~300 genotypes over six years, allowing all the uses described in this data note. Through collaborations, this set can be expanded to ~800 genotypes.

Regular measurements with the FIP 1.0 head were accompanied by reference measurement campaigns as part of several projects. The heading date and senescence ratings were performed to investigate the relationships of senescence dynamics and diseases [15, 16, 17]. Yield measurements taken on the FIP field were combined with data from other locations to train phenomic prediction models [18]. The plant height measurements served as a basis to quantify the temperature response of wheat genotypes in the stem elongation phase [8, 9, 12]. The extracted plant height values demonstrated their usefulness in improving trait extraction methods from longitudinal data [19, 20, 21].

The images collected allowed to quantify canopy cover values [22] and examine their relationship to frost damage events [23] using Convolutional Neural Networks (CNNs). Using a combination of drone data and the high-resolution images the rows in the individual plots were identified [11]. In a small subset (375 images), the wheat heads were annotated and the data was integrated into the public global wheat head detection data set [24]. The image-based canopy cover values served as a test data set to evaluate the cultivar-specific extensions of the thermal time concept [25].

The culmination of these efforts has resulted in a unique, multi-dimensional data set. Dense image time series of diverse wheat genotypes are integrated with trait measurements, genetic markers, and environmental data. The data set has been designed to align with FAIR principles [26]:

- **Findable**: This publication and the Hugging Face data set card (https://doi.org/10.57967/hf/3191) provide detailed metadata and a comprehensive description of the data set's contents, making it discoverable to researchers.
- **Accessible**: The data is hosted on the Research Collection of ETH Zurich (https://doi.org/20.500.11850/697773), a reliable and openly accessible data storage.
- **Interoperable**: The use of the open-source Hugging Face datasets [27] package makes it easy to use and export to different formats. The data is fully MIAPPE v1.1 [28] conform. Given the shared genotypes the data set can be used to enhance the data by Gogna et al. [13] by 6 environments, to a total of 14 environments. The data set expands on existing sub-sets of the data already released that can be used as baseline approaches such as [8, 9, 19, 20, 21, 12, 25, 15, 16, 17].
- **Reusable**: The data is released under the CC0 1.0 Universal license (https://creativecommons.org/publicdomain/zero/1.0/), a permissive license that allows for further use of the data.

Materials and Methods

Experimental Field Designs and Genotypes

All experiments were performed at the ETH research station of plant sciences in Lindau Eschikon, Switzerland (47.449 N, 8.682 E, 556 m a.s.l.). The soil characteristics were determined in 2015 (Eric Schweizer AG, Thun, Switzerland). The soil type is eutric cambisol

Compiled on: February 27, 2025.
Draft manuscript prepared by the author.

consisting of 21% clay and 21% silt with an organic matter content of 3.5% and pH 6.7. A crop rotation was implemented during and before the start of wheat experiments beginning with a year of soybean (*Glycine max* (L.) Merr.), then a year of buckwheat (*Fagopyrum esculentum* Moench) and finally wheat (*Triticum aestivum* L.). After preliminary crops were harvested, the soil was plowed and harrowed before wheat was drill-sown. The wheat was sown in 9 rows per plot with a row length of ~1.7 m, a row distance of 0.125 m and a sowing density of 370–400 plants m^{-2}.

A few days after sowing, herbicide (Herold SC, Bayer AG, Leverkusen, Germany) was applied to ensure weed-free plots. Several fungicides and insecticides were applied in spring to ensure healthy plants. The fertilizer was split into three doses (~ 1:3:1), one at tillering stage, one at start of stem elongation, and one after heading. Approximately 140 kg N, 90 kg P_2O_5, and 100 kg K_2O per ha were applied, depending on site-specific soil analysis. No irrigation was applied. The sowing and harvest dates for each year are provided in Table 1.

For 2016–2018, a GABI-WHEAT panel subset (consisting of ~300 European winter wheat cultivars from the GABI-WHEAT panel [14, 29]) was complemented by 35–52 Swiss winter wheat varieties of commercial importance. For 2019, a small subset of the GABI-WHEAT panel (54 genotypes) was grown alongside genotypes from other experiments (e.g., Swiss variety testing). In 2019, these other genotypes were grown on larger plots with a row length of 5.5 m while GABI-WHEAT genotypes were grown on the same size plots as in the other years. In 2021 and 2022, sets of F8 genotypes from the Swiss breeding program of Agroscope (Nyon, Switzerland) were grown. For an overview of genotype overlaps between years, see Figure 2.

For all years, an experimental design following the principles of good practice—replication, randomization, and blocking [30]—was chosen. The described panels of, on average, 350 genotypes per year were replicated once, each replication was randomized and grown on a different lot in the FIP area (Figure 1). Each replication was augmented with checks in a 3×3 block arrangement. For further details on the experimental design, see [8, 9, 12].

Image Data

The FIP system is divided into two independent parts, (1) the carrier system built and maintained by Spidercam (Spidercam GmbH, Feistritz im Rosental, Austria), and (2) the custom-built FIP 1.0 sensor head and control software [7].

Carrier: The Spidercam Cable-Suspended System

The carrier system, detailed in Kirchgessner et al. [7], uses four corner-mounted poles with pulleys. Cables connect these pulleys to winches, enabling 3-D movement of the FIP 1.0 sensor head. A working distance of 2–3 m from the canopy was maintained during measurements.

Sensors: The FIP 1.0 Imaging Head

The FIP 1.0 sensor head carried, amongst other sensors, a 21 MP full frame DSLR camera (EOS 5D Mark II, 35 mm lens (Canon Inc., Tokyo, Japan) [7]. The camera was triggered automatically via a custom MATLAB script (The Mathworks [31]). The images were mostly captured using auto white balance, auto exposure, an ISO of 100, an exposure time of 1/250 second (4 ms), and zero exposure bias value. The ground sampling distance is approximately 0.55 mm.

Image Registration

The positions of the captured images of the plots change throughout the season due to inaccuracies in the camera carrier system and intentional height adjustments due to plant growth. To allow for consistent image feature extraction, the time series needed to be aligned using an image registration pipeline. Image registration transforms the images so that points that are at the same physical location in the real world are aligned to the same point in the image planes. The used image registration pipeline consists of two steps: a deep learning-based feature matcher and a transformation estimation step. The feature matcher is used to find features that correspond to the same location between an image pair, which are then used to estimate the transformation between the images. The registration was performed between image pairs instead of the whole sequence directly, commonly referred to as image alignment.

To predict aligned polygons of the inner rows (Figure 1) for an image time series three individual steps were performed. First an initial reference polygon was aligned with a drone orthomosaic or prior data. Then subsequent aligned polygons were found based on this initial polygon or a previously aligned polygon. Finally, a single transformation between alignments and the inner plots was estimated and applied to all aligned polygons. The aligned result of a time series can be seen in Figure 3.

To find the initial reference polygon, the 3D-world-coordinates of the plot corners were first extracted from drone-based orthomosaics (2018–2022), from other projects [32, 10, 11, 33, 18, 12], or from extrapolations of these plot corners to earlier years (2016–2017). The 3D plot corners from this initial extraction process were matched to the best-fitting image in the image time series to find their 2D counterparts, which were used as the initial reference polygon.

Using the initial reference image and polygon, the initial image was matched with other images and the resulting transformation used to find the other aligned plot corners. Matching features between different time points in a crop season is challenging due to significant changes in conditions between images, such as varying lighting, plant growth and changing appearance of the plants. For this reason, a modified version of the deep learning LoFTR [34] feature matcher was fine-tuned to focus on consistent soil features, such as stones. Even these consistent objects slowly change their positions throughout the season or rapidly between strong precipitation events. Therefore, the transformations were estimated between image pairs and not the complete time series at a time. The estimated transformations between the image pairs are homographies based on the soil plane.

A prior alignment strategy employing Scale-invariant feature transform (SIFT) based feature matching required manual alignment of about 10,000 image pairs to fill gaps where the matcher failed. While suitable for smaller scale projects, this approach was deemed unviable for the complete data set. These manual alignments, created by choosing four point-pairs to find suitable homographies, were used to fine-tune the LoFTR feature matcher. The homographies correspond to the transformations between the common soil plane of the image pairs. This simplification was used to fine-tune the model to find matches solely in the soil plane, as features in the canopy are at incorrect positions due to not being on the homography plane. Since the canopy moves, sometimes significantly, between time-points, this reduces the number of incorrect matches that the feature matcher produces. The model was fine-tuned for 20 epochs with randomly cropped, rotated and distorted image pairs. These augmentations were applied to each image individually, adjusting the homographies accordingly. The augmentations were chosen such that the image pairs were always partially overlapping.

To verify the correctness of the matches and their homographies, several checks were employed. Basic checks, such as ensuring enough inliers, constraining the ratios of the side lengths and angles of the polygon spanned by the plot corners to their 3D counterparts, and other image-based checks were applied with high thresholds. The essential matrix was calculated, and the warped plot corners were compared to their closest point on the epipolar line. The essential matrix was used to triangulate the 3D points of the plot corners, and they were compared to the original 3D-

world-coordinates by checking the ratios and angles of the polygons spanned by the plot corners. These ratios were used to scale the predicted relative camera movement and compare it individually to the maximum possible movements in each axis. Finally, these predictions and checks were done twice with the image pair swapped and compared to each other. This check filtered cases where LoFTR predicted structured noise that corresponded to the identity homography between the image pairs. To filter outliers these steps were iteratively repeated, and the best prediction based on the checks was chosen.

When an image could not be matched to the initial image, it was iteratively matched with the closest found alignment, based on the number of transformations and then the date differences. Matches were used to find homographies using OpenCV [35], which were then used to warp the plot corners (Figure 1, 'Plot').

The complete process, except the initial 3D-world-coordinates extraction, was iteratively repeated by going from very tight to looser restrictions in the checks. The fine-tuned LoFTR model was bootstrapped, that is, further trained at each step by adding the trusted subset of its predictions that passed the checks in the previous step to the training set.

Finally after predicting all plot corners, a relative inner plot polygon was extracted to mitigate border effects and further correct the alignment. The seven inner rows of plants were detected based on segmented images showing plant and soil pixels. Then, plots were further rectified by rotating them step-wise (-1.5° to 1.5° in steps of 0.2°) to maximize the distance between the minimum and maximum numbers of plant pixels in image columns [25]. Inner plots were filtered if they did not contain the complete plot.

In total, the alignment and inner plot detection was successful for more than 95% of all images. The number of successfully aligned images per year can be seen in Table 2. Both the 160,772 original images and their corresponding 153,022 inner plot cutouts are made available as part of this data set.

Image Preprocessing

The raw images were converted to PNG format, an accessible and lossless image format, using rawpy [36] with minimal post-processing, reducing resolution to half size, employing a linear demosaicing algorithm, and disabling auto adjustments in order to preserve the original sensor data with minimal artifacts or alterations. The inner plots were directly cut out of the pre-processed image by combining the plot and its relative inner plot transformation.

Reference Measurements

As part of several projects [8, 9, 15, 16, 18, 12], reference measurements such as grain yield and growth stage ratings were taken. Those traits can be divided into low-level traits (time series of traits that develop over time), intermediate traits (traits extracted from low-level traits that describe the growth dynamics over time [20, 19]) and target traits (observations that are usually targeted in breeding and agriculture, e.g., yield) (Table 7). Trait measurements may have been made at times different from the image captures. All measurements are scalars that correspond to a single measurement at a given time point during the season.

Low-level Traits

Plant height measurements were performed with a TLS (Focus 3D S 120, 905 nm laser, Faro Technologies Inc., Lake Mary, USA) for 2016 and 2017 on the same date as image acquisition [8]. From 2019–2022, drone RGB images [10, 11] were used to extract plant height estimations with Structure from Motion (SfM) [37]. FIP image collection dates and drone campaign dates were typically within days of each other but did not necessarily overlap. The accuracy of TLS and drone measurements to approximate manual plant height measurements were demonstrated in the respective publications (TLS [8]: R^2: 0.99, drone [37]: R^2: 0.96). The two methods are in good accordance with one another (R^2: 0.99, intercept: 0.057 m, slope: 1.0 [12]).

From images, canopy cover was extracted using a deep learning model described in Zenkl et al. [22], a model that reached a pixel accuracy of 0.945 on a FIP test set. The percentage of soil covered by plant parts was determined for the seven-row inner plot (Figure 1) as described in Tschurr et al. [38]. On the same seven-row inner plot, wheat head count estimations were determined for all dates from May to end of season. As the wheat head detection method, the winning model of the global wheat head challenge [24] (`https://github.com/ksnxr/GWC_solution`) was used, a model that achieved an average domain accuracy of 0.7 on a test set that included FIP images.

Senescence was assessed visually in 2016, 2017 and 2018 from approximately 20 days after flowering to full senescence for the central plot area canopy, following guidelines provided by Pask et al. [39]. Plot senescence was scored according to Anderegg et al. [16] based on the portion of green leaf area on a scale from 0 to 10, equivalent to 0 to 100 %. To avoid bias, manual ratings were performed by the same expert in all three years.

The number of total measurements, as well as the minimum, maximum, and mean number of measurements per year for each low-level trait, can be seen in Table 3.

Intermediate Traits

Heading date was visually assessed as the date when 50% of the spikes were fully emerged from the flag leaf sheath [17] (BBCH 59, [40]). To ensure consistent ratings over time, heading date ratings were started at approximately BBCH 55 and continue to BBCH 61, with two to three rating events per week.

Final height was extracted from TLS or SfM plant height measurements using the Quarter of Maximum Elongation Rate (QMER) method described in Roth et al. [20], which has a reported accuracy of close to 1.0 on simulated data. The number of measurements per year for intermediate and target traits can be seen in Table 4.

Target Traits

Yield for small plots in all years was estimated based on two rows of nine rows (row seven and eight). Ears within these two rows were hand-harvested, dried for at least 24 hours at 30 °C, and threshed using a stand thresher (Saatmeister Allesdrescher K35; Saatzucht Baumann, Germany). Yield for large plots in 2019 was determined with a combine harvester (Nursery- master Elite; Wintersteiger, Ried im Innkreis, Austria). Weight was determined using a scale, and water content with a Wile 55 moisture meter (Farmcomp Oy; FIN04360 Tuusula, Finland). Grain yield was mathematically normalized to 14 % water content. Grain protein content was determined using near-infrared transmission spectroscopy (InfratecTM 1241 Grain Analyzer; Foss, DK-3400 Hilleroed, Denmark).

In 2019, plot sizes varied between experiments, which influences yield measurements. While large plot sizes deliver absolute estimates comparable with METs, in our experience, small plot sizes tend to overestimate yield per area. To compensate for this effect, adjusted genotype means (see next Section) of twelve common genotypes between the experiments with large and small plots were used to calculate a conversion factor. A linear regression with intercept zero estimated a conversion factor of 1.50 from large to small plots (R^2 of 0.47). This conversion factor was used to transform all yield measurement estimates of small plots to the range of large plots, resulting in a new trait 'Grain yield (adjusted)'.

Adjusted Genotype Means within Year Calculation

All intermediate and target traits were processed to adjusted genotype means (Best Linear Unbiased Estimate (BLUE)) using a linear

mixed model in SpATS,

$$y_{ijk} = m_c + g_i + p_{r(k)} + p_{c(k)} + S(r(k)), c(k)) + e_{ijk}, \quad (1)$$

where y_{ijk} is the measured trait value for the ith genotype in the jth year for plot k in row $r(k)$ and column $c(k)$. m_c is a fixed effect marking check varieties ($m_c \in [0, 1]$), g_i is a fixed genotype effect, $p_{r(k)}$ and $p_{c(k)}$ are random spatial row and column effects, and e_{ijk} a spatially independent residual. $S(r(k)), c(k))$ is a spatial smooth surface in row and column direction as defined in [41].

Environmental Covariates

Air temperature, relative humidity, short wavelength solar irradiance, and soil temperature were measured at a local weather station in proximity to the experimental field above or below a grass strip [25]. Air temperature and relative humidity were measured 2 m and 0.1 m above ground, short wavelength solar irradiance 2 m above ground, and soil temperature 0.05 m below ground, respectively. Precipitation data were taken from a close-by Agrometeo weather station at Strickhof (<800 m, https://www.agrometeo.ch). Measurement gaps caused by technical issues (e.g., sensor failure) were filled with data from the Strickhof station if available and with data from a Meteoswiss station at the Zurich Airport (9.5 km, https://gate.meteoswiss.ch/idaweb/) otherwise. Overall, more than 97% of the data after gap filling originated from the local weather station or Strickhof weather station.

Genetic Marker Data

Genetic marker data (90k SNP array) for the GABI-WHEAT panel are publicly available [29]. The private marker data were created using a 25k SNP array. Two sets were compiled: A pure GABI-WHEAT marker data set, and an extended marker set consisting of overlapping marker from both public and private markers. Marker locations on the reference genome IWGSC RefSeq v1.0 were collected with InterMine [42] from https://urgi.versailles.inrae.fr and complemented with locations previously mapped with blastn from an earlier project [12].

SNPs in both sets were first filtered for minor allele frequency (5%) and missing values (5%). Genotypes in both sets were then tested for missing marker data with a missing rate of 1% (all passed, no genotype had to be removed). Finally, missing marker data (0.44% for GABI-WHEAT set, 0.54% for extended set) were imputed based on a k-nearest-neighbour implementation in R (scrime [43]) per chromosome. Markers were sorted by chromosome and location for later use in local connectivity models such as CNNs. Markers with ambiguous positions were left in the set but marked accordingly.

The resulting GABI-WHEAT genetic marker set includes 372 genotypes and 18'846 markers. The extended set includes 824 genotypes and 11'943 markers. Based on the marker data, kinship matrices [44] were calculated according to Yang [45].

Compilation as Data Set

Train/Test Split According to Genetic Relatedness

The most interesting application of trait prediction approaches is to predict the performance of unseen genotypes in unseen years [46, 47]. The accuracy of such predictions typically depends on the relatedness of genotypes, usually assessed through cross-validation. However, cross-validation is computationally expensive for complex deep learning models. To address this issue, we propose an alternative approach that balances the train/test set using genetic relatedness [48]. This method theoretically yields performance close to the average of all cross-validation runs.

To implement this approach, we used the R package STPGA [49] to determine a test set complementing the training set with the algorithm 'D_{opt}' as suggested in [50]. The training set was further split into training and validation sets using the same method. We define four test sets to allow for specific evaluation of methods depending on their use. The test sets differ based on if their genotypes and environments occur in the train set (denoted as seen):

i. Test (P): Unseen plots with seen genotypes and seen environments (all years except 2019)
ii. Test (G): Unseen genotypes with seen environments
iii. Test (E): Unseen environment (2019) with seen genotypes
iv. Test (G and E): Unseen genotypes and unseen environment (2019)

To validate our balanced splitting approach, we also performed five-fold cross-validations, allowing for a quality check of the balanced splits. The splits were calculated separately for the pure GABI-WHEAT set and for the extended set. The extended set additionally includes F8 generation genotypes with private marker data. For the GABI-WHEAT set, this resulted in 262 genotypes in the training set, 24 in the validation set, and 30 in Test (G and E) test set (Figure 5). For the extended set, this resulted in 747 genotypes in the training set, 30 in the validation set, and 30 in Test (G and E) test set.

Preparation as Hugging Face Data Set

All data was aggregated into a single table with a row for each plot containing the image sequence, the aligned image sequence, traits, environmental data, marker data, and additional metadata. The table was split using the aforementioned splits and converted to a single Hugging Face data sets DatasetDict using the schema shown in Table 8. The table and DatasetDict contain None values for completely missing entries. Missing data in sequences are absent.

Data Validation and Quality Control

Heritabilities of Low-level Traits

Heritability is a statistic used in breeding to quantify how much of a trait's variation is attributable to the examined genetic material, ranging from 0 to 1. For field phenotyping traits, heritability can indicate the quality of a trait, as one is interested in methods that extract highly genotype-specific values, i.e., traits with high heritability. To assess the quality of low-level traits, heritability was calculated for each time point by setting the genotype factor g_i in Equation 1 to random and estimating genetic and non-genetic variances, as described by Oakey et al. [51]. The resulting heritabilities (Figure 4) follow an expected temporal pattern: increasing with growth and decreasing towards the end of the growth phase. These findings align with previously reported values for the same data set, e.g. 0.77 for senescence traits (2016-2018) [16] and 0.61/0.59 for derived plant height traits (start/stop growth) [12]. Additionally, plant height measurements obtained using TLS have been shown to strongly correlate with drone-based height estimations (correlation of 0.99) [12].

Heritabilities of Intermediate and Target Traits

To test for the quality of intermediate and target traits, heritability was calculated according to Oakey et al.[51] by setting the genotype factor g_i in Equation 1 to random and estimating genetic and non-genetic variances. The results (Table 5) are in accordance with values reported for the same data set before, e.g., 0.55 for grain yield in 2016–2018 [16], 0.97 for heading date in 2016–2018 [16], 0.84 for grain protein content in 2016 and 2017 [16], and 0.98 for final height in 2015–2018 [12]. The quality of the public marker data set for the GABI-WHEAT panel was demonstrated in Gogna et al. by means of testing for genomic prediction ability [13]. The same

quality check was performed on the data set presented herein, once with the public marker data from the GABI-WHEAT panel, once with the extended set that includes private marker data as well.

Three different GBLUP-based genomic prediction models were trained: One linear mixed model with simple main effect and identity variance (ID), one with simple main effect and diagonal variance (DIAG), and one with a simple main effect and random regression to environmental covariates [52]. As environmental covariates, metrics were based on the Standardized Precipitation and Evapotranspiration Index (SPEI) [53], Vapour Pressure Deficit (VPD), air temperature at 2.0 m above ground, and precipitation. For the SPEI and temperature, the mean, maximum and minimum value over the season were used, for VPD, the mean and maximum, and for precipitation, the sum of the values was used. All models were implemented in ASReml-R using code by [54].

Results are reported for the three scenarios unseen genotypes in unseen environment (Test (G and E), Table 9), unseen environments (Test (E), Table 10), and unseen genotypes (Test (G), Table 11). The test (G) corresponds to the scenario reported in Gogna et al. [13]. Results show comparable performance for heading date and final height, superior performance for protein content, and inferior performance for grain yield (Table 11). These results are in accordance to the heritabilities found for the traits (Table 5). If comparing models, no notable difference in performance was found.

Genomic Prediction Ability of Unseen Multi-environment Trial

Gogna et al. have published a MET data set for yield, protein content, heading date and final height comprising eight environments (five locations and one to two years) for the GABI-WHEAT panel [13]. 312 of the measured genotypes overlap with the FIP 1.0 data set, 60 are unseen in the FIP 1.0 data set but marker data are available. The unseen genotypes (60) in unseen environments (Test (G and E)) and seen genotypes (312) in unseen environments (Test (E)) were taken as independent test sets in new environments for a genomic prediction approach similar to the one described in the previous section. For the random regression model, hourly temperature, precipitation and relative humidity data for the German environments were extracted from the Climate Data Center (CDC) of the German Weather Service, those for the French environments from Meteo France (SYNOP, 3-hourly data only). These additional environmental covariate data and MET data are available in the data repository for convenience, but not part of the core data set (see folder 'MET_repository_clone').

For grain yield, the random regression model outperformed the other models (Table 9). For all other traits, no clear advantage of the random regression model over the other models was visible. Again, the results suggest comparable accuracies to Gogna et al. [13] for final height, heading date and protein content, and inferior performance for grain yield (Table 9).

Re-use Potential and Limitations

In this work, we provide baselines for genomic prediction approaches, trait extractions from images, and subsequent trait dynamics modeling. Accordingly, we see the largest re-use potential of the presented data set for the development and evaluation of new modelling and prediction approaches in crop genomics and phenomics. The multi-faceted data set allows modelling approaches on various levels:

i. Genomic prediction approaches that include genotype-environment interactions: The presented data enhance the data by Gogna et al. [13] by 6 environments, totalling to 14 environments that are characterized by environmental covariates. The presented benchmark of a genomic prediction with random regressions to environmental covariates [52] provides a baseline that novel approaches can challenge.
ii. Modelling plant growth and development with longitudinal modelling approaches: The four low-level traits canopy cover, plant height, wheat head count and senescence cover the full growing season of winter wheat in 6 environments that are characterized by environmental covariates. Baseline approaches for plant height growth modelling [8, 9, 19, 20, 21, 12], canopy cover growth modelling [25] and senescence dynamics modelling [15, 16, 17] for subsets of the presented data exist.
iii. Image-based phenomic predictions and combined phenomic and genomic prediction approaches: The dense time series of images allow training and analysing end-to-end modelling approaches (e.g., deep learning based) that predict target traits such as yield based on images.

While the data set opens up the possibility of analysing HTFP data to a wide audience, it also has its inherent limitations that should be taken into account if working with it:

- The immobility of the FIP restricts the data set to only one location.
- Field-based data collection introduces various sources of errors that one must consider in analysis (see e.g. [20] for a discussion).
- Yield measurements in the FIP and hence this data set are more prone to error than in METs.
- Annotation at the image level only requires further annotation effort if semantic segmentation or object detection methods are targeted.
- While the aligned image time series provide extensive opportunities to analyze growth dynamics, this kind of highly preprocessed image data is rare and therefore interoperability with other data sources is yet limited.

Examples

To run the following examples the Huggingface *datasets* [27] library is required. The examples were run using version *3.3.2*.

Example 1: Basic access to data set via hugging face datasets package

```
import datasets
fip1 = datasets.load_dataset("mikeboss/FIP1")
fip1_train = fip1["train"]
print(fip1_train)
# Output:
Dataset({
    features: ['plot_uid', 'yearsite_uid', 'crop_type', 'experiment_number', 'plot_number', 'range', 'row', 'lot', 'latitude', 'longitude', 'spatial_check', 'sowing_date', ...],
    num_rows: 2930
})
```

Listing 1. Example 1

Example 2: Load aligned inner plot cutouts as stacked numpy array

```
fip1 = fip1.select_columns("inner_plot_images")
fip1 = fip1.cast_column("inner_plot_images", datasets.Sequence(datasets.Image()))
fip1 = fip1.with_format("numpy")
print(fip1["train"][0]["inner_plot_images"].shape)
# Output:
```

```
(35, 640, 960, 3)
```

Listing 2. Example 2

Example 3: Access low-level trait time series

```
print(fip1["train"][0]["height_values"])
# Output:
[np.float16(0.2231), np.float16(0.2725), np.float16(0.3188)
    , ...]
```

Listing 3. Example 3

Example 4: Access specific target traits

```
fip1 = fip1.select_columns(["yield_value", "protein_value",
     "height_final_value", "heading_value"])
print(fip1["train"][0])
# Output:
{'yield_value': np.float16(8.64), 'protein_value': np.
    float16(13.74), 'height_final_value': np.float16
    (1.062), 'heading_value': np.float16(1591.0)}
```

Listing 4. Example 4

Example 5: Access marker data

```
fip1 = fip1.with_format("numpy")
print(fip1["train"][0]["marker_biallelic_codes"])
print(fip1["train"][0]["marker_metadata_strings"])
# Output:
[2 2 2 ... 2 2 2]
['0000000001:chr1A:1145398:1145498:BS00000713_51_T_C'
 '0000000003:chr1A:1174887:1174987:
    Excalibur_c10657_1280_C_T'
 '0000000004:chr1A:1176337:1176337:
    wsnp_Ex_c10657_17376086_C_T' ...
 'wsnp_bf474966A_Ta_2_1_T_C' 'wsnp_bm138650D_Ta_2_2_G_A'
 'wsnp_cd454041D_Ta_2_1_C_T']
```

Listing 5. Example 5

Example 6: Access environmental data in January

```
fip1 = fip1.select_columns(
    ["temperature_air_10cm_values", "
     temperature_air_10cm_dates"]
)
fip1 = fip1.map(
    lambda values, dates: {
        "temperature_air_10cm_values_january": [
            value for value, date in zip(values, dates,
     strict=True) if date.month == 1
        ]
    },
    input_columns=["temperature_air_10cm_values", "
     temperature_air_10cm_dates"],
)
print(fip1["train"][0]["temperature_air_10cm_values_january
    "][:10])
# Output:
[np.float16(2.6), np.float16(2.7), np.float16(2.8), ...]
```

Listing 6. Example 6

Availability of Source Code and Requirements

The code to recreate the derived data and the data set is publicly available in three repositories, namely the *FIP 1.0 Data Set - Traits*, *fip1-alignment*, and *fip1-dataset* repositories.

The complete process to create the data set involves extracting trait data from the raw data using the *FIP 1.0 Data Set - Traits* repository, then aligning the image time-series using the *fip1-alignment* repository, and finally aggregating the derived data into the final data set using the *fip1-dataset* repository.

In addition, the data set can be recreated using the *fip1-dataset* repository from the derived data that is freely available in the ETH research collection.

Trait Data Compilation

Project name: FIP 1.0 Data Set - Traits
Project home page: https://gitlab.ethz.ch/crop_phenotyping/fip-1.0-data-set-traits
Operating system(s): Platform independent
Programming language: R, Python
License: GNU GPL v3

Image Data Alignment

Project name: fip1-alignment
Project home page: https://gitlab.ethz.ch/crop_phenotyping/fip1-alignment
Operating system(s): Platform independent
Programming language: Python
License: GNU GPL v3

Data Set Compilation

Project name: fip1-dataset
Project home page: https://gitlab.ethz.ch/crop_phenotyping/fip1-dataset
Operating system(s): Platform independent
Programming language: Python
License: GNU GPL v3

Data Availability

- Data Repository: http://doi.org/20.500.11850/697773
- Hugging Face Data set: https://huggingface.co/datasets/mikeboss/FIP1
- Public GABI marker data repository (also integrated in main Data Repository and Hugging Face Data set): https://doi.org/10.5061/dryad.n02v6wwzc
- Private Agroscope marker data repository: Confidential (Contact: Boulos Chalhoub, boulos.chalhoub@agroscope.admin.ch). This repository contains marker data (Illumina Infinium 25k array) from eight generation (F8) breeding lines that are unregistered and property of Agroscope. Access can be requested by stating the intended purpose of use and the willingness to sign a material transfer agreement (MTA).

Declarations

Glossary

F8 Eighth Generation Breeding Material. 2, 3, 5

RGB Red, Green and Blue. 2, 4

Acronyms

BLUE Best Linear Unbiased Estimate. 4

CNNs Convolutional Neural Networks. 2, 5

FIP Field Phenotyping Platform. 2–4, 6, 9
FIP 1.0 Field Phenotyping Platform 1.0. 2, 3, 6, 14

GABI-WHEAT Genomanalyse im Biologischen System Pflanze - Weizen. 2, 3, 5, 6, 9, 11

HTFP High-throughput field phenotyping. 2, 6

INVITE Innovation in Variety Testing. 2

MET Multi-Environment Trial. 2, 4, 6

QMER Quarter of Maximum Elongation Rate. 4

SfM Structure from Motion. 4
SIFT Scale-invariant feature transform. 3
SpATS Spatial Analysis of Field Trials with Splines. 5
SPEI Standardized Precipitation and Evapotranspiration Index. 6

TLS Terrestrial Laser Scanner. 2, 4, 5

VPD Vapour Pressure Deficit. 6

Consent for Publication

Not applicable.

Competing Interests

The author(s) declare that they have no competing interests.

Funding

A.W. discloses support for the research of this work from Swiss National Science Foundation [grant number 169542 and 200756]. L.R. discloses support for the research of this work from Swiss Data Science Center [grant number PHENO-MINE C21-04].

Author's Contributions

Lukas Roth: Conceptualization, Methodology, Software, Validation, Formal analysis, Investigation, Data Curation, Writing - Original Draft, Visualization, Supervision, Funding acquisition. Mike Boss: Conceptualization, Methodology, Software, Validation, Formal analysis, Investigation, Data Curation, Writing - Original Draft, Visualization. Norbert Kirchgessner: Conceptualization, Methodology, Software, Validation, Formal analysis, Investigation, Data Curation, Writing - Original Draft, Visualization. Helge Aasen: Investigation, Supervision. Brenda Patricia Aguirre-Cuellar: Investigation. Price Pius Atuah Akiina: Investigation. Jonas Anderegg: Methodology, Data Curation, Investigation. Joaquin Gajardo Castillo: Software, Data Curation. Xiaoran Chen: Investigation. Simon Corrado: Investigation. Krzysztof Cybulski: Software, Data Curation. Beat Keller: Investigation, Supervision. Stefan Göbel Kortstee: Investigation. Lukas Kronenberg: Methodology, Data Curation, Investigation. Frank Liebisch: Investigation, Supervision. Paraskevi Nousi: Investigation. Corina Oppliger: Investigation. Gregor Perich: Investigation. Johannes Pfeifer: Investigation. Kang Yu: Investigation. Nicola Storni: Software, Data Curation, Investigation. Flavian Tschurr: Software, Data Curation, Investigation. Michele Volpi: Investigation, Supervision. Simon Treier: Investigation, Data Curation. Hansueli Zellweger: Investigation. Olivia Zumsteg: Investigation. Andreas Hund: Conceptualization, Methodology, Writing - Review & Editing, Supervision, Project administration. Achim Walter: Conceptualization, Writing - Review & Editing, Supervision, Project administration, Funding acquisition.

Acknowledgements

Not applicable.

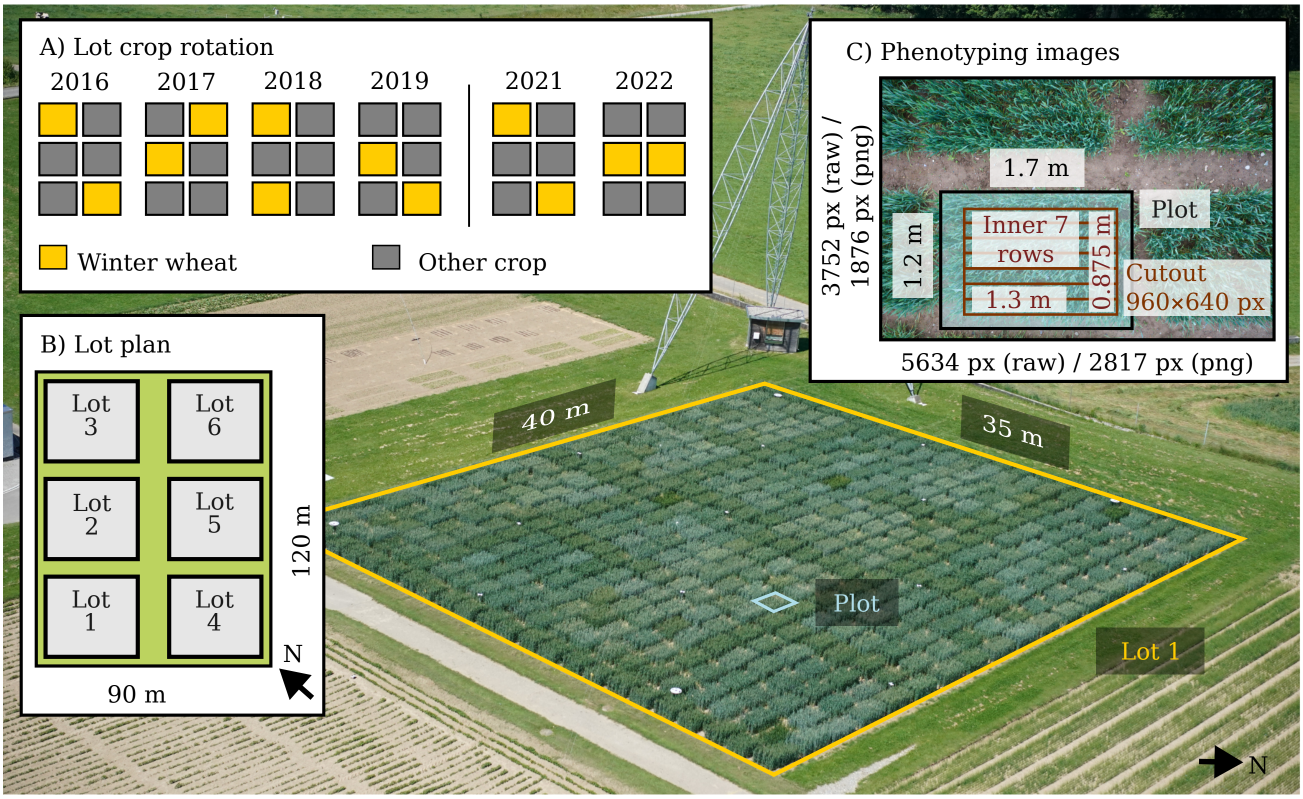

Figure 1. The data set source: 12 wheat lots in 6 years (2016–2022) with >350 wheat plots each, resulting in >4,000 plots from which >160,000 images were taken. The background image shows the Field Phenotyping Platform (FIP) lot 1 with wheat plots. All wheat lots were integrated in a regular crop rotation with other crops (A) according to a permanent lot plan (B). Images taken with the FIP show one complete plot each, the positions of the complete plot and the inner 7 rows are annotated (C).

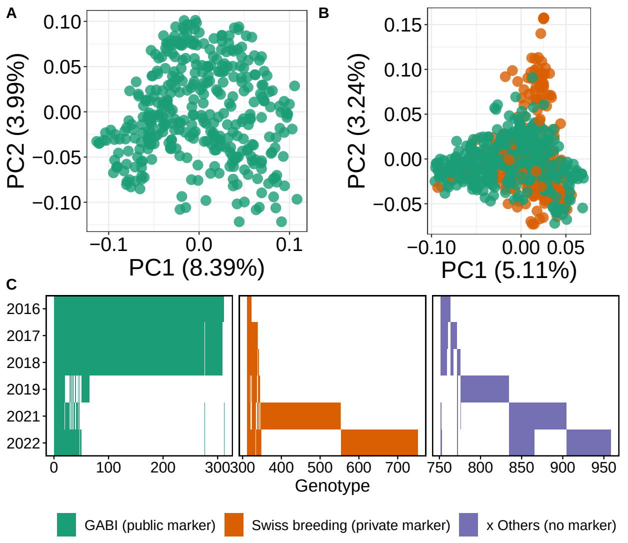

Figure 2. The examined genotype sets with their genetic relatedness visualized in SNP-marker based principal component analysis based on public GABI-WHEAT markers (A) and public and private markers combined (B), and year of cultivation of genotypes (C).

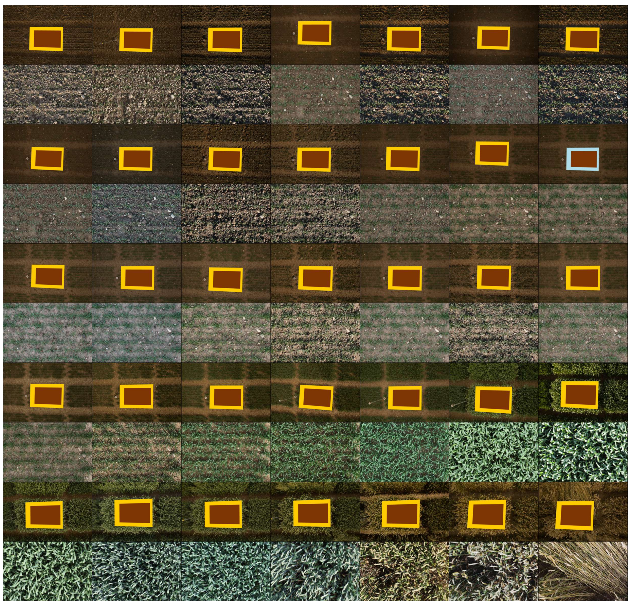

Figure 3. An image time series of a plot over one season with the aligned inner plot cutouts in each second row. The initial alignment polygon, shown in light blue, is based on alignment with a drone orthophoto. All other alignment polygons, shown in orange, are transformed from the initial polygon or another aligned polygon. The inner plot polygons, shown in brown, are based on a single transform from an aligned polygon to the inner rows of the plot. Images are adjusted for visibility.

Table 1. Sowing and harvest dates

Year	Sowing Date	Harvest Date
2016	2015-10-13	2016-07-27
2017	2016-11-01	2017-07-19
2018	2017-11-02	2018-07-14
2019	2018-10-17	2019-07-23
2021	2020-10-21	2021-07-29
2022	2021-11-25	2022-07-19

Table 2. Data set sizes for original images and aligned images per year. For each category, the number of images and minimum / maximum / mean time-series length are given.

Year	Plots	Original images				Aligned images			
		# Images	Min	Max	Mean	# Images	Min	Max	Mean
2016	710	26237	35	39	36.95	25566	26	39	36.01
2017	756	19717	23	30	26.08	14534	9	29	19.22
2018	756	21642	26	31	28.63	21266	12	31	28.13
2019	252	7451	28	34	29.57	6445	20	34	25.57
2021	792	48451	58	63	61.17	48285	58	63	60.97
2022	792	37274	44	51	47.06	36926	44	51	46.62
Total	4058	160772	23	63	39.62	153022	9	63	37.71

Table 3. Time-series data set sizes for low-level traits. For each low-level trait the number of measurements and minimum / maximum / mean time-series length are given.

Year	Plots	Canopy Cover				Plant Height				Wheat head count				Senescence rating			
		#	Min	Max	Mean	#	Min	Max	Mean	#	Min	Max	Mean	#	Min	Max	Mean
2016	710	710	27	39	36.00	710	22	22	22.00	710	1	11	9.51	703	0	9	8.78
2017	756	756	9	24	18.52	756	21	21	21.00	756	1	18	9.77	756	1	10	9.77
2018	756	756	12	27	21.50	756	36	43	39.72	755	0	18	10.35	756	12	12	12.00
2019	252	252	8	25	21.89	252	38	46	44.55	228	0	10	3.57	–	–	–	–
2021	792	792	55	61	59.21	792	45	48	46.56	792	30	36	33.96	–	–	–	–
2022	792	792	31	41	38.46	792	42	45	42.53	792	12	24	21.55	–	–	–	–
Total	4058	4058	8	61	34.18	4058	21	48	35.13	4057	0	36	16.47	2215	0	12	5.59

Table 4. Data set sizes and key characteristics for genotypes as well as intermediate and target traits. Numbers describe absolute sizes, i.e., how many plots and genotypes were examined in a specific year, and how many of this plots/time series are annotated with target traits grain yield, grain protein content, heading date, and final height.

Year	Plots	Genotypes	Grain yield / Grain yield (adjusted)	Protein content	Heading date	Final height
2016	710	335	710	685	710	710
2017	756	352	754	0	377	756
2018	756	353	756	674	369	756
2019	252	90	248	144	177	252
2021	792	346	661	781	453	792
2022	792	373	744	792	719	792
Total	4058	904	3873	3076	2805	4058

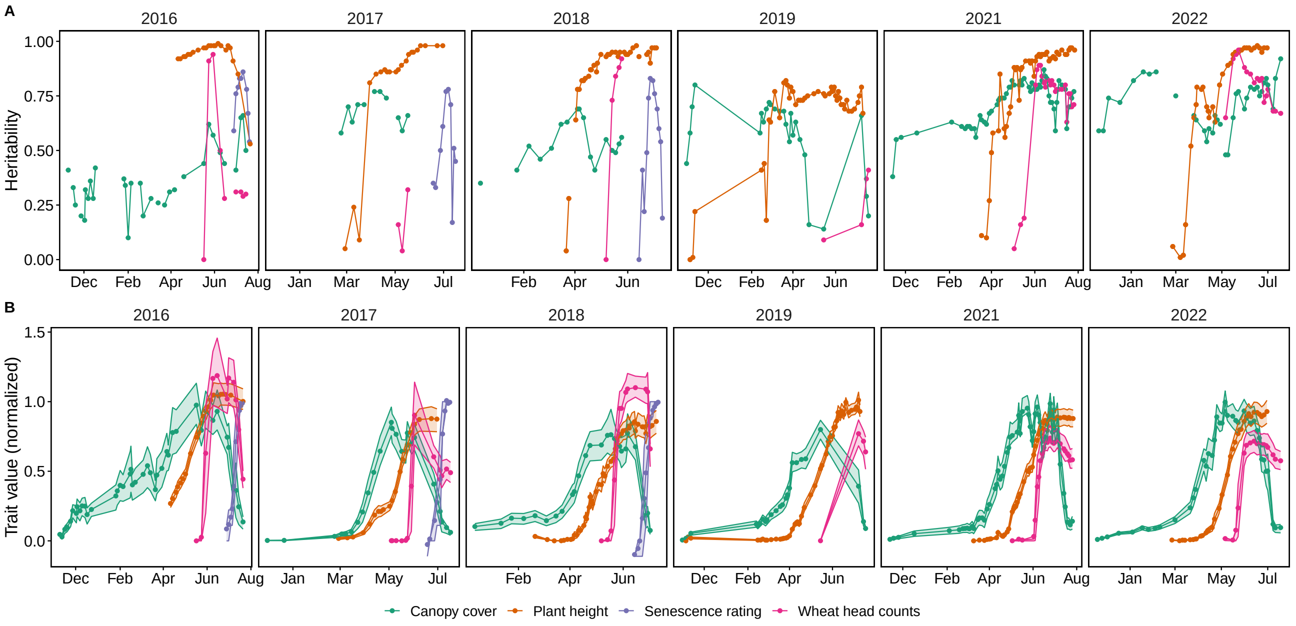

Figure 4. Heritability of the four low-level traits per point in time for all years (2016–2022) (A) and normalized measured trait values (B). Indicated are means (points) and the 25% and 75% percentiles (areas).

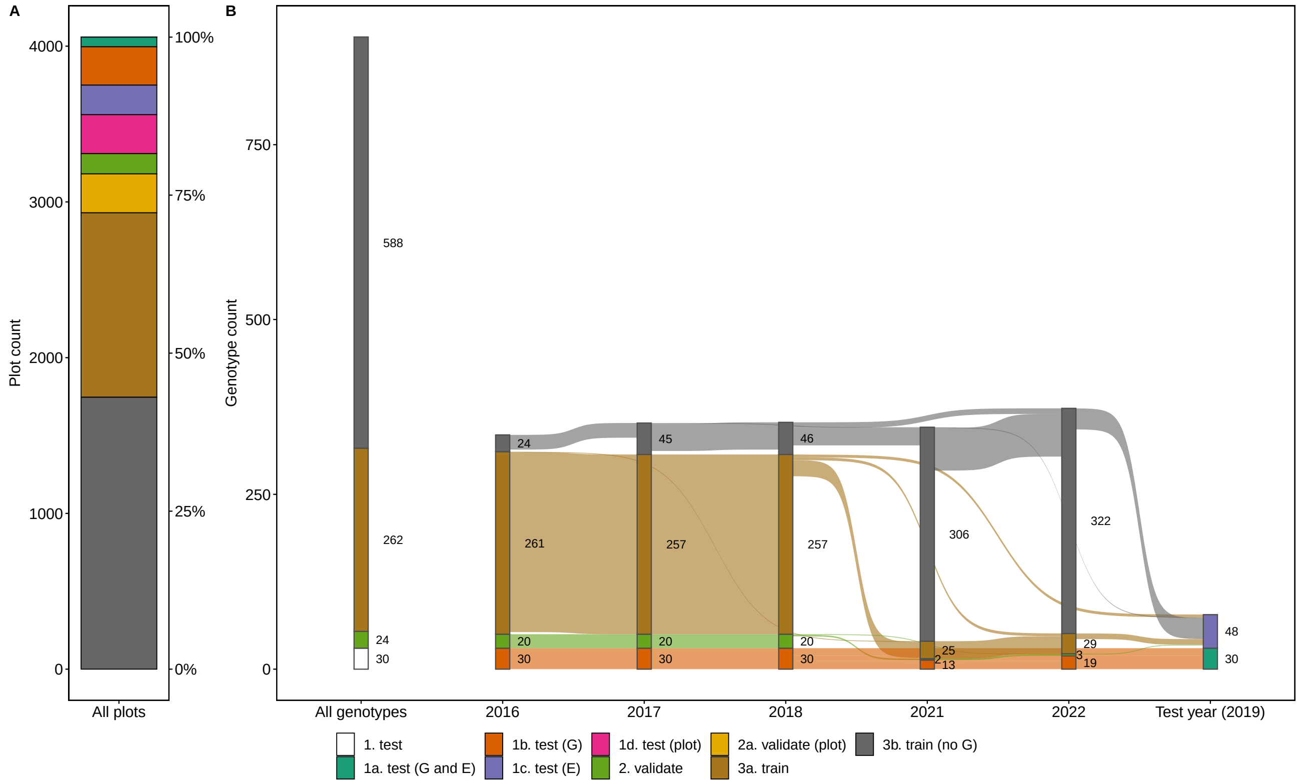

Figure 5. Training, validation and test splits for the GABI-WHEAT marker data based genotype set. The test set includes four subsets, unseen genotypes (G), unseen environments (E), unseen genotypes in unseen environments (G and E), and seen genotypes in seen years on unseen plots (plots). The validation set includes two subsets, unseen genotypes (default) and seen genotypes on unseen plots (plots). The splits are shown based on plots (A) and based on genotype count (B), genotypes occurring in different sets are reported at the lowest level (train < validation < test).

Table 5. Heritabilities (H^2) of intermediate and target traits for all years.

Trait	Heritability (H^2)					
	2016	2017	2018	2019	2021	2022
Grain yield	0.58	0.36	0.33	0.79	0.41	0.48
Grain yield (adjusted)	(s)	(s)	(s)	0.83	(s)	(s)
Protein content	0.82	-	0.81	0.80	0.55	0.86
Heading date	0.96	N/A	0.90	0.89	0.81	0.93
Final height	0.97	0.98	0.97	0.93	0.96	0.97

N/A: Unreplicated measurement, heritability not available
-: No measurements in this year
Grain yield (adjusted): Plot size adjusted yield ($yield_{5\,m} = yield_{1\,m} / 1.50$)
(s): All plot same size, see results for "Grain yield"

Table 6. Variance decomposition results of intermediate and target traits for all years.

Trait	Component	Percentage of total variance					
		2016	2017	2018	2019	2021	2022
Grain yield	Genotype	8%	11%	1%	1%	18%	3%
	Spatial	81%	52%	94%	98%	41%	91%
	Residual	11%	37%	5%	1%	41%	6%
Grain yield (adjusted)	Genotype	(s)	(s)	(s)	28%	(s)	(s)
	Spatial	(s)	(s)	(s)	59%	(s)	(s)
	Residual	(s)	(s)	(s)	13%	(s)	(s)
Protein content	Genotype	1%	-	2%	11%	7%	6%
	Spatial	98%	-	97%	85%	83%	92%
	Residual	0%	-	1%	4%	11%	2%
Heading date	Genotype	61%	N/A	58%	57%	77%	35%
	Spatial	34%	N/A	36%	29%	2%	61%
	Residual	4%	N/A	6%	14%	20%	4%
Final height	Genotype	60%	87%	5%	19%	6%	12%
	Spatial	36%	9%	95%	77%	94%	88%
	Residual	3%	3%	0%	3%	0%	1%

N/A: Unreplicated measurement, variance components not available
-: No measurements in this year
Grain yield (adjusted): Plot size adjusted yield ($yield_{5\,m} = yield_{1\,m} / 1.50$)
(s): All plots same size, see results for "Grain yield"

Table 7. Measured traits that were used to annotate image time series. Label refers to the name used in the data set for the trait.

Trait	Unit	Label	Description
**Low-level traits**			
Canopy cover	(0...1)	canopy_cover	Soil coverage by plants from a nadir view
Plant height	m	height	Distance top of soil to top of canopy
Wheat head count	Count	spike_count	Number of visible wheat spikes
Senescence rating	(0...10)	senescence	End-of-season leaf decay ratings
**Intermediate traits**			
Heading	Date	heading	Date when 50% of wheat spikes visible
Final height	m	height_final	Plant height at end-of-season
**Target traits**			
Grain yield	t/ha	yield	Total weight of harvested grains
Grain yield (adjusted)	t/ha	yield_adjusted	Plot size adjusted yield
Protein content	%	protein	Grain protein content

Table 8. A schema of the data set equivalent to the Hugging Face data set schema. Sequence indicates a variable length of values while Array2D indicates a fixed shape of the data.

Field Name	Type	Field Name	Type
**identifiers**		height_final_value	float16
plot_uid	string	height_final_date	date32
yearsite_uid	string	height_final_blue	float16
crop_type	string	height_final_heritability	float16
experiment_number	uint8	height_final_trait_id	uint8
plot_number	int16	height_final_trait_name	string
**location**		height_final_method_id	uint8
range	uint8	height_final_method_name	string
row	uint8	height_final_si_unit	string
lot	uint8	height_final_responsible	string
latitude	float64	**target traits**	
longitude	float64	yield_value	float16
spatial_check	int32	yield_date	date32
**dates**		yield_blue	float16
sowing_date	date32	yield_heritability	float16
harvest_date	date32	yield_trait_id	uint8
harvest_year	uint16	yield_trait_name	string
**images**		yield_method_id	uint16
images	Sequence[string]	yield_method_name	string
image_dates	Sequence[date32]	yield_si_unit	string
image_times	Sequence[time32[s]]	yield_responsible	string
**alignments**		yield_adjusted_value	float16
alignment_plot_soil_polygons	Sequence[Array2D[float16]]	yield_adjusted_date	date32
alignment_num_steps	Sequence[uint8]	yield_adjusted_blue	float16
alignment_dates	Sequence[date32]	yield_adjusted_heritability	float16
alignment_times	Sequence[time32[s]]	yield_adjusted_trait_id	uint8
alignment_initial_date	date32	yield_adjusted_trait_name	string
alignment_inner_plot_transform	Array2D[float16]	yield_adjusted_method_id	uint16
inner_plot_images	Sequence[string]	yield_adjusted_method_name	string
image_inner_plot_transforms	Sequence[Array2D[float16]]	yield_adjusted_si_unit	string
**markers**		yield_adjusted_responsible	string
genotype_id	string	protein_value	float16
marker_biallelic_codes	Sequence[uint8]	protein_date	date32
marker_metadata_strings	Sequence[string]	protein_blue	float16
**low-level traits**		protein_heritability	float16
canopy_cover_values	Sequence[float16]	protein_trait_id	uint8
canopy_cover_dates	Sequence[date32]	protein_trait_name	string
canopy_cover_trait_ids	Sequence[uint8]	protein_method_id	uint16
canopy_cover_trait_name	Sequence[string]	protein_method_name	string
canopy_cover_method_ids	Sequence[uint16]	protein_si_unit	string
canopy_cover_method_name	Sequence[string]	protein_responsible	string
canopy_cover_si_unit	string	**environment**	
canopy_cover_responsible	string	temperature_air_10cm_values	Sequence[float16]
height_values	Sequence[float16]	temperature_air_10cm_dates	Sequence[date32]
height_dates	Sequence[date32]	temperature_air_10cm_times	Sequence[time32[s]]
height_trait_ids	Sequence[uint8]	temperature_air_200cm_values	Sequence[float16]
height_trait_name	Sequence[string]	temperature_air_200cm_dates	Sequence[date32]
height_method_ids	Sequence[uint16]	temperature_air_200cm_times	Sequence[time32[s]]
height_method_name	Sequence[string]	temperature_soil_5cm_values	Sequence[float16]
height_si_unit	string	temperature_soil_5cm_dates	Sequence[date32]
height_responsible	string	temperature_soil_5cm_times	Sequence[time32[s]]
spike_count_values	Sequence[float16]	humidity_air_10cm_values	Sequence[float16]
spike_count_dates	Sequence[date32]	humidity_air_10cm_dates	Sequence[date32]
spike_count_trait_ids	Sequence[uint8]	humidity_air_10cm_times	Sequence[time32[s]]
spike_count_trait_name	Sequence[string]	humidity_air_200cm_values	Sequence[float16]
spike_count_method_ids	Sequence[uint16]	humidity_air_200cm_dates	Sequence[date32]
spike_count_method_name	Sequence[string]	humidity_air_200cm_times	Sequence[time32[s]]
spike_count_si_unit	string	precipitation_200cm_values	Sequence[float16]
spike_count_responsible	string	precipitation_200cm_dates	Sequence[date32]
senescence_values	Sequence[float16]	precipitation_200cm_times	Sequence[time32[s]]
senescence_dates	Sequence[date32]	irradiance_solar_200cm_values	Sequence[float16]
senescence_trait_ids	Sequence[uint8]	irradiance_solar_200cm_dates	Sequence[date32]
senescence_trait_name	Sequence[string]	irradiance_solar_200cm_times	Sequence[time32[s]]
senescence_method_ids	Sequence[uint16]		
senescence_method_name	Sequence[string]		
senescence_si_unit	string		
senescence_responsible	string		
**intermediate traits**			
heading_value	float16		
heading_date	date32		
heading_blue	float16		
heading_heritability	float16		
heading_trait_id	uint8		
heading_trait_name	Sequence[string]		
heading_method_id	uint16		
heading_method_name	Sequence[string]		
heading_si_unit	string		
heading_responsible	string		

Table 9. Genomic prediction accuracy (correlation) and bias (RMSE) of intermediate and target traits for unseen genotypes in unseen environments (Test (G and E)).

Split set	Trait	ID			DIAG			RREG			ID			DIAG			RREG		
		Correlation									RMSE								
		B	CV	MET	B	CV	MET	B	CV	MET	B	CV	MET	B	CV	MET	B	CV	MET
GABI	Grain Yield	0.19	0.35	0.22	0.18	0.34	0.24	0.16	0.33	0.34	1.25	1.32	1.45	1.25	1.33	1.45	1.52	1.67	1.09
GABI	Grain Yield (adjusted)	0.16	0.34	0.21	0.16	0.34	0.23	0.15	0.33	0.31	1.57	1.67	2.52	1.57	1.67	2.51	1.86	1.99	3.1
GABI	Protein Content	0.36	0.46	0.46	0.34	0.45	0.46	0.33	0.43	0.46	1.11	1.13	1.23	1.13	1.14	1.23	0.65	0.67	1.55
GABI	Heading Date	0.59	0.55	0.67	0.59	0.55	0.67	0.59	0.55	0.67	6.57	7.31	5.77	6.54	7.28	5.77	5.35	6.08	5.86
GABI	Plant Height	0.8	0.75	0.81	0.8	0.74	0.81	0.57	0.61	0.75	0.06	0.07	0.08	0.06	0.06	0.08	0.18	0.17	0.17
Extended	Grain Yield	0.17	0.32	0.27	0.17	0.32	0.28	0.17	0.31	0.41	1.27	1.27	1.42	1.26	1.27	1.42	1.51	1.59	1.1
Extended	Grain Yield (adjusted)	0.23	0.31	0.27	0.23	0.31	0.27	0.23	0.3	0.37	1.66	1.86	2.51	1.66	1.86	2.5	1.92	2.13	3.03
Extended	Protein Content	0.37	0.64	0.52	0.37	0.64	0.53	0.36	0.63	0.52	1.12	1.06	1.25	1.12	1.07	1.25	0.81	0.79	1.55
Extended	Heading Date	0.6	0.53	0.66	0.61	0.57	0.68	0.62	0.55	0.64	6.95	6.65	5.73	6.95	6.66	5.71	5.59	5.3	5.88
Extended	Plant Height	0.8	0.76	0.82	0.8	0.78	0.81	N/A	0.65	0.8	0.05	0.05	0.08	0.05	0.05	0.08	N/A	0.34	0.17

ID: Identical variances; DIAG: Varying variances per year; RREG: Random regression to environmental covariates; N/A: Failed convergence
Test set: B: Balanced FIP 1.0 data set; CV: 5-fold cross-validation on FIP 1.0 data set; MET: Unseen multi-environment trial data set

Table 10. Genomic prediction accuracy (correlation) and bias (RMSE) of intermediate and target traits for seen genotypes in unseen environments (Test (E)).

Split set	Trait	ID			DIAG			RREG			ID			DIAG			RREG		
		Correlation									RMSE								
		B	CV	MET	B	CV	MET	B	CV	MET	B	CV	MET	B	CV	MET	B	CV	MET
GABI	Grain Yield	0.73	0.36	0.26	0.74	0.35	0.27	0.67	0.3	0.33	1.52	1.31	1.53	1.53	1.32	1.53	1.97	1.68	1.11
GABI	Grain Yield (adjusted)	0.76	0.37	0.26	0.76	0.36	0.27	0.69	0.31	0.31	1.97	1.74	2.4	1.98	1.74	2.4	2.31	2.04	2.99
GABI	Protein Content	0.92	0.79	0.52	0.93	0.78	0.52	0.92	0.77	0.52	0.83	0.95	1.37	0.84	0.96	1.36	0.34	0.49	1.74
GABI	Heading Date	0.94	0.9	0.9	0.94	0.9	0.9	0.94	0.9	0.89	7.22	7.05	5.06	7.2	7.04	5.06	5.97	5.78	5.16
GABI	Plant Height	0.96	0.97	0.92	0.95	0.97	0.92	0.64	0.87	0.72	0.04	0.03	0.08	0.04	0.03	0.08	0.14	0.15	0.18
Extended	Grain Yield	0.61	0.38	0.29	0.62	0.39	0.3	0.59	0.37	0.37	1.19	1.23	1.52	1.18	1.22	1.52	1.55	1.51	1.15
Extended	Grain Yield (adjusted)	0.69	0.46	0.29	0.7	0.47	0.29	0.68	0.45	0.35	1.85	1.74	2.38	1.85	1.74	2.38	2.14	2	2.91
Extended	Protein Content	0.87	0.83	0.53	0.87	0.83	0.53	0.86	0.81	0.53	0.9	0.95	1.38	0.91	0.96	1.38	0.55	0.58	1.72
Extended	Heading Date	0.89	0.89	0.9	0.89	0.89	0.9	0.89	0.89	0.89	6.55	6.56	5.04	6.56	6.56	5.04	5.15	5.14	5.21
Extended	Plant Height	0.94	0.95	0.92	0.94	0.95	0.92	N/A	0.87	0.91	0.04	0.04	0.08	0.04	0.04	0.08	N/A	0.34	0.16

ID: Identical variances; DIAG: Varying variances per year; RREG: Random regression to environmental covariates; N/A: Failed convergence
Test set: B: Balanced FIP 1.0 data set; CV: 5-fold cross-validation on FIP 1.0 data set; MET: Unseen multi-environment trial data set

Table 11. Genomic prediction accuracy (correlation) and bias (RMSE) of intermediate and target traits for unseen genotypes in seen environments (Test (G)).

Split set	Trait	ID		DIAG		RREG		ID		DIAG		RREG	
		Correlation						RMSE					
		B	CV	B	CV	B	CV	B	CV	B	CV	B	CV
GABI	Grain Yield	0.45	0.44	0.44	0.42	0.45	0.44	1.04	1	1.04	1	1.04	1
GABI	Grain Yield (adjusted)	0.45	0.44	0.44	0.42	0.45	0.44	0.69	0.66	0.69	0.66	0.69	0.66
GABI	Protein Content	0.61	0.58	0.62	0.58	0.61	0.59	0.75	0.88	0.73	0.87	0.75	0.87
GABI	Heading Date	0.59	0.53	0.59	0.53	0.59	0.53	2.08	2.24	2.1	2.24	2.08	2.24
GABI	Plant Height	0.83	0.7	0.83	0.71	0.76	0.69	0.07	0.08	0.07	0.08	0.08	0.08
Extended	Grain Yield	0.48	0.37	0.47	0.39	0.48	0.37	0.99	1.04	0.99	1.04	0.99	1.04
Extended	Grain Yield (adjusted)	0.48	0.37	0.47	0.39	0.48	0.37	0.65	0.69	0.65	0.69	0.65	0.69
Extended	Protein Content	0.7	0.79	0.71	0.79	0.7	0.79	0.68	0.69	0.68	0.7	0.68	0.69
Extended	Heading Date	0.63	0.53	0.65	0.58	0.64	0.53	1.93	2.13	1.88	2.05	1.91	2.11
Extended	Plant Height	0.82	0.67	0.83	0.71	N/A	0.63	0.06	0.07	0.06	0.07	N/A	0.07

ID: Identical variances; DIAG: Varying variances per year; RREG: Random regression to environmental covariates; N/A: Failed convergence
Test set: B: Balanced FIP 1.0 data set; CV: 5-fold cross-validation on FIP 1.0 data set

References

1. Tilman D, Balzer C, Hill J, Befort BL. Global Food Demand and the Sustainable Intensification of Agriculture. Proceedings of the National Academy of Sciences 2011;108(50):20260–20264.
2. Pretty J, Sutherland WJ, Ashby J, Auburn J, Baulcombe D, Bell M, et al. The Top 100 Questions of Importance to the Future of Global Agriculture. International Journal of Agricultural Sustainability 2010;8(4):219–236.
3. Martre P, Dueri S, Guarin JR, Ewert F, Webber H, Calderini D, et al. Global needs for nitrogen fertilizer to improve wheat yield under climate change. Nature Plants 2024;10(7):1081–1090.
4. Kiss T, Dixon LE, Soltész A, Bányai J, Mayer M, Balla K, et al. Effects of Ambient Temperature in Association with Photoperiod on Phenology and on the Expressions of Major Plant Developmental Genes in Wheat (Triticum Aestivum L.). Plant, Cell & Environment 2017;40(8):1629–1642.
5. White JW, Hoogenboom G, Kimball BA, Wall GW. Methodologies for Simulating Impacts of Climate Change on Crop Production. Field Crops Research 2011 Dec;124(3):357–368.
6. Araus JL, Kefauver SC, Zaman-Allah M, Olsen MS, Cairns JE. Translating High-Throughput Phenotyping into Genetic Gain. Trends in Plant Science 2018;23(5):451–466.
7. Kirchgessner N, Liebisch F, Yu K, Pfeifer J, Friedli M, Hund A, et al. The ETH Field Phenotyping Platform FIP: A Cable-Suspended Multi-Sensor System. Functional Plant Biology 2017;44:154–168.
8. Kronenberg L, Yu K, Walter A, Hund A. Monitoring the Dynamics of Wheat Stem Elongation: Genotypes Differ at Critical Stages. Euphytica 2017;213(157).
9. Kronenberg L, Yates S, Boer MP, Kirchgessner N, Walter A, Hund A. Temperature Response of Wheat Affects Final Height and the Timing of Stem Elongation under Field Conditions. Journal of Experimental Botany 2020;.
10. Roth L, Hund A, Aasen H. PhenoFly Planning Tool: Flight Planning for High-Resolution Optical Remote Sensing with Unmanned Areal Systems. Plant Methods 2018;14(116).
11. Roth L, Camenzind M, Aasen H, Kronenberg L, Barendregt C, Camp KH, et al. Repeated Multiview Imaging for Estimating Seedling Tiller Counts of Wheat Genotypes Using Drones. Plant Phenomics 2020;2020(3729715).
12. Roth L, Kronenberg L, Aasen H, Walter A, Hartung J, van Eeuwijk F, et al. High-Throughput Field Phenotyping Reveals That Selection in Breeding Has Affected the Phenology and Temperature Response of Wheat in the Stem Elongation Phase. Journal of Experimental Botany 2024 Mar;75(7):2084–2099.
13. Gogna A, Schulthess AW, Röder MS, Ganal MW, Reif JC. Gabi Wheat a Panel of European Elite Lines as Central Stock for Wheat Genetic Research. Scientific Data 2022;9(538).
14. Kollers S, Rodemann B, Ling J, Korzun V, Ebmeyer E, Argillier O, et al. Whole Genome Association Mapping of Fusarium Head Blight Resistance in European Winter Wheat (Triticum Aestivum L.). PLoS ONE 2013;8(2).
15. Anderegg J, Hund A, Karisto P, Mikaberidze A. In-Field Detection and Quantification of Septoria Tritici Blotch in Diverse Wheat Germplasm Using Spectral–Temporal Features. Frontiers in Plant Science 2019;10(1355).
16. Anderegg J, Yu K, Aasen H, Walter A, Liebisch F, Hund A. Spectral Vegetation Indices to Track Senescence Dynamics in Diverse Wheat Germplasm. Frontiers in Plant Science 2020;10(1749).
17. Anderegg J, Aasen H, Perich G, Roth L, Walter A, Hund A. Temporal Trends in Canopy Temperature and Greenness Are Potential Indicators of Late-Season Drought Avoidance and Functional Stay-Green in Wheat. Field Crops Research 2021;274(108311).
18. Roth L, Fossati D, Krähenbühl P, Walter A, Hund A. Image-based Phenomic Prediction Can Provide Valuable Decision Support in Wheat Breeding. Theoretical and Applied Genetics 2023 Jun;136(7):162.
19. Roth L, Piepho HP, Hund A. Phenomics Data Processing: Extracting Dose-Response Curve Parameters from High-Resolution Temperature Courses and Repeated Field-Based Wheat Height Measurements. in silico Plants 2022;4(1).
20. Roth L, Rodríguez-Álvarez MX, van Eeuwijk F, Piepho HP, Hund A. Phenomics Data Processing: A Plot-Level Model for Repeated Measurements to Extract the Timing of Key Stages and Quantities at Defined Time Points. Field Crops Research 2021;274(108314).
21. Pérez-Valencia DM, Rodríguez-Álvarez MX, Boer MP, Kronenberg L, Hund A, Bosquet LC, et al. A Two-stage Approach for the Spatio-temporal Analysis of High-throughput Phenotyping Data. Scientific Reports 2022;12(3177).
22. Zenkl R, Timofte R, Kirchgessner N, Roth L, Hund A, Van Gool L, et al. Outdoor Plant Segmentation With Deep Learning for High-Throughput Field Phenotyping on a Diverse Wheat Dataset. Frontiers in Plant Science 2022;12(774068).
23. Tschurr F, Kirchgessner N, Hund A, Kronenberg L, Anderegg J, Walter A, et al. Frost Damage Index: The Antipode of Growing Degree Days. Plant Phenomics 2023 Sep;0(ja).
24. David E, Madec S, Sadeghi-Tehran P, Aasen H, Zheng B, Liu S, et al. Global Wheat Head Detection (GWHD) Dataset: A Large and Diverse Dataset of High-Resolution RGB-Labelled Images to Develop and Benchmark Wheat Head Detection Methods. Plant Phenomics 2020;2020(3521852).
25. Roth L, Binder M, Kirchgessner N, Tschurr F, Yates S, Hund A, et al. From Neglecting to Including Cultivar-Specific Per Se Temperature Responses: Extending the Concept of Thermal Time in Field Crops. Plant Phenomics 2024 Jun;6:0185.
26. Wilkinson MD, Dumontier M, Aalbersberg IJ, Appleton G, Axton M, Baak A, et al. The FAIR Guiding Principles for Scientific Data Management and Stewardship. Scientific Data 2016 Dec;3(160018).
27. Lhoest Q, Villanova del Moral A, Jernite Y, Thakur A, von Platen P, Patil S, et al. Datasets: A Community Library for Natural Language Processing. In: Proceedings of the 2021 Conference on Empirical Methods in Natural Language Processing: System Demonstrations Online and Punta Cana, Dominican Republic: Association for Computational Linguistics; 2021. p. 175–184. https://aclanthology.org/2021.emnlp-demo.21.
28. Papoutsoglou EA, Faria D, Arend D, Arnaud E, Athanasiadis IN, Chaves I, et al. Enabling Reusability of Plant Phenomic Datasets with MIAPPE 1.1. New Phytologist 2020;227:260–273.
29. Gogna A, Börgel AWS, Röder M, Ganal MW, Reif JC, The Genotypic Data of Elite European Cultivar Panel Comprising 358 Winter and 14 Summer Wheat Varieties Released from 1975 to 2007 at Different Marker Densities. Dryad; 2022.
30. Kempton RA. The Design and Analysis of Unreplicated Field Trials. Vorträge für Pflanzenzüchtung 1984;7:219–242.
31. Inc TM, MATLAB version: 9.13.0 (R2022b). Natick, Massachusetts, United States: The MathWorks Inc.; 2022. https://www.mathworks.com.
32. Roth L, Aasen H, Walter A, Liebisch F. Extracting Leaf Area Index Using Viewing Geometry Effects—A New Perspective on High-Resolution Unmanned Aerial System Photography. ISPRS Journal of Photogrammetry and Remote Sensing 2018;141:161–175.
33. Roth L, Barendregt C, Bétrix CA, Hund A, Walter A. High-Throughput Field Phenotyping of Soybean: Spotting an Ideotype. Remote Sensing of Environment 2022;269(112797).
34. Sun J, Shen Z, Wang Y, Bao H, Zhou X. LoFTR: Detector-free local feature matching with transformers. In: Proceedings of the IEEE/CVF conference on computer vision and pattern recognition; 2021. p. 8922–8931.
35. Bradski G. The OpenCV Library. Dr Dobb's Journal of Software Tools 2000;.

36. Rierchert M. rawpy: RAW image processing for Python, a wrapper for libraw. github 2021;.
37. Roth L, Streit B. Predicting Cover Crop Biomass by Lightweight UAS-based RGB and NIR Photography: An Applied Photogrammetric Approach. Precision Agriculture 2018;19:93–114.
38. Tschurr F, Feigenwinter I, Fischer AM, Kotlarski S. Climate Scenarios and Agricultural Indices: A Case Study for Switzerland. Atmosphere 2020;11.
39. Pask A, Pietragalla J, Mullan D, Reynolds M, editors. Physiological Breeding II: A Field Guide to Wheat Phenotyping. Mexico, D.F.: CIMMYT; 2012.
40. Lancashire PD, Bleiholder H, Van den Boot T, Langelüddeke P, Strauss R, Weber E, et al. A Uniform Decimal Code for Growth Stages of Crops and Weeds. Annals of Applied Biology 1991;119:561–601.
41. Rodríguez-Álvarez MX, Boer MP, van Eeuwijk FA, Eilers PHC. Correcting for spatial heterogeneity in plant breeding experiments with P-splines. Spatial Statistics 2017;23:52 – 71.
42. Smith RN, Aleksic J, Butano D, Carr A, Contrino S, Hu F, et al. InterMine: A Flexible Data Warehouse System for the Integration and Analysis of Heterogeneous Biological Data. Bioinformatics (Oxford, England) 2012 Dec;28(23):3163–3165.
43. Schwender H, with a contribution of Arno Fritsch. scrime: Analysis of High-Dimensional Categorical Data Such as SNP Data; 2018, https://CRAN.R-project.org/package=scrime, r package version 1.3.5.
44. VanRaden PM. Efficient Methods to Compute Genomic Predictions. Journal of Dairy Science 2008;91(11):4414–4423.
45. Yang J, Benyamin B, McEvoy BP, Gordon S, Henders AK, Nyholt DR, et al. Common SNPs Explain a Large Proportion of the Heritability for Human Height. Nature Genetics 2010 Jul;42(7):565–569.
46. Malosetti M, Bustos-Korts D, Boer MP, van Eeuwijk FA. Predicting Responses in Multiple Environments: Issues in Relation to Genotype x Environment Interactions. Crop Science 2016;56:2210–2222.
47. Piepho HP. Prediction of and for New Environments: What's Your Model? Molecular Plant 2022;15(4):581–582.
48. Bustos-Korts D, Malosetti M, Chapman S, Biddulph B, van Eeuwijk F. Improvement of Predictive Ability by Uniform Coverage of the Target Genetic Space. G3 Genes|Genomes|Genetics 2016 Nov;6(11):3733–3747.
49. Akdemir D. STPGA: Selection of Training Populations by Genetic Algorithm; 2018, https://CRAN.R-project.org/package=STPGA, r package version 5.2.1.
50. Akdemir D, Isidro-Sánchez J. Design of Training Populations for Selective Phenotyping in Genomic Prediction. Scientific Reports 2019 Feb;9(1):1446.
51. Oakey H, Verbyla A, Pitchford W, Cullis B, Kuchel H. Joint Modeling of Additive and Non-Additive Genetic Line Effects in Single Field Trials. Theoretical and Applied Genetics 2006;113:809–819.
52. Jarquín D, Crossa J, Lacaze X, Du Cheyron P, Daucourt J, Lorgeou J, et al. A Reaction Norm Model for Genomic Selection Using High-Dimensional Genomic and Environmental Data. Theoretical and Applied Genetics 2014 Mar;127(3):595–607.
53. Beguería S, Vicente-Serrano SM, Reig F, Latorre B. Standardized Precipitation Evapotranspiration Index (SPEI) Revisited: Parameter Fitting, Evapotranspiration Models, Tools, Datasets and Drought Monitoring. International Journal of Climatology 2014;34(10):3001–3023.
54. Tolhurst DJ, Gaynor RC, Gardunia B, Hickey JM, Gorjanc G. Genomic Selection Using Random Regressions on Known and Latent Environmental Covariates. Theoretical and Applied Genetics 2022;(i).

Manuscript differences to first submission
Click here to access/download;Manuscript;FIP1.0_20250227_diff.pdf

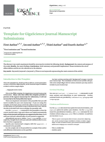

GigaScience, 0000, 1–16

doi: xx.xxxx/xxxx
Manuscript in Preparation
Data Note

DATA NOTE

The FIP 1.0 Data Set: Highly Resolved Annotated Image Time Series of 4,000 Wheat Plots Grown in Six Years

Lukas Roth[1,†], Mike Boss[1,†], Norbert Kirchgessner[1,†,*], Helge Aasen[1,a], Brenda Patricia Aguirre-Cuellar[1,b], Price Pius Atuah Akiina[1,c], Jonas Anderegg[1,d], Joaquin Gajardo Castillo[1], Xiaoran Chen[2], Simon Corrado[1], Krzysztof Cybulski[1,e], Beat Keller[1], Stefan Göbel Kortstee[1,f], Lukas Kronenberg[1,g], Frank Liebisch[1,h], Paraskevi Nousi[2], Corina Oppliger[1], Gregor Perich[1], Johannes Pfeifer[1,i], Kang Yu[1,k], Nicola Storni[1], Flavian Tschurr[1], Simon Treier[1], Michele Volpi[2], Hansueli Zellweger[1,l], Olivia Zumsteg[1], Andreas Hund[1] and Achim Walter[1]

[1]ETH Zürich, Institute of Agricultural Sciences, Zürich, Switzerland and [2]ETH Zürich and EPFL, Swiss Data Science Center, Switzerland and [a]Agroscope, Earth Observation of Agroecosystems Team, Zürich, Switzerland and [b]Universidad Nacional de Colombia, Facultad de Ciencias Agropecuarias, Bogotá, Colombia and [c]University of Wyoming, Department of Plant Sciences, Laramie, Wyoming, USA and [d]ETH Zurich, Plant Pathology Group, Zürich, Switzerland and [e]ETH Zurich, Seminar for Statistics, Zürich, Switzerland and [f]Sítio Florosa, São Paulo, Brazil and [g]The John Innes Centre, Crop Genetics, Norwich, United Kingdom and [h]Agroscope, Water Protection and Substance Flows Team, Zürich, Switzerland and [i]Federal Office for Agriculture and Food, Bonn, Germany and [k]Technical University of Munich, Precision Agriculture Lab, Freising, Germany and [l]Departement für Inneres und Volkswirtschaft (DIV), Landwirtschaftsamt, Thurgau, Switzerland

[*]corresponding author: Norbert Kirchgessner (norbert.kirchgessner@usys.ethz.ch)
[†]equal contribution
[‡] current affiliations listed as a–k

Abstract

Background: Understanding genotype-environment interactions of plants is crucial for crop improvement, yet limited by the scarcity of quality phenotyping data. This data note presents the Field Phenotyping Platform 1.0 data set, a comprehensive resource for winter wheat research that combines imaging, trait, environmental, and genetic data.
Findings: We provide time series data for more than 4,000 wheat plots, including aligned high-resolution image sequences totaling more than ~~151~~153,000 aligned images across six years. Measurement data for eight key wheat traits is included, namely canopy cover values, plant heights, wheat head counts, senescence ratings, heading date, final plant height, grain yield, and protein content. Genetic marker information and environmental data complement the time series. Data quality is demonstrated through heritability analyses and genomic prediction models, achieving accuracies aligned with previous research.
Conclusions: This extensive data set offers opportunities for advancing crop modeling and phenotyping techniques, enabling researchers to develop novel approaches for understanding genotype-environment interactions, analyzing growth dynamics, and predicting crop performance. By making this resource publicly available, we aim to accelerate research in climate-adaptive agriculture and foster collaboration between plant science and ~~computer vision~~ machine learning communities.

Key words: Winter wheat; High-throughput phenotyping; Field phenotyping platform; Yield; Protein content; Image time series; Deep learning data set

Data Description

Aim

Winter wheat provides a crucial share of calories for human nutrition, with global demand steadily increasing [1]. However, crop production faces challenges due to limited resources like water, agrochemicals, and land [2]. Climate change further threatens crop yields, necessitating responsible and efficient resource use [3].

Crop yields are substantially driven by complex interactions between plant genetics and environmental factors. For instance, genes involved in fruit formation interact with temperatures at flowering, influencing growth and yield potential [4]. Limited phenotyping data is seen as the major reason for the incomplete understanding of such genotype-environment interactions [5].

High-throughput field phenotyping (HTFP) was developed to address this data gap [6]. Imaging HTFP platforms allow researchers to monitor crop canopy development over time, generating dense time series data of plant growth. There are many approaches to process such data ranging from extracting traits at critical time points to modeling growth dynamics and finally using end-to-end methods that directly analyze image time series.

This data set aims to provide a comprehensive foundation for these diverse approaches. Our goal is to foster collaboration between plant physiology, biometrics, and computer vision research, ultimately improving the ability to predict genotype-environment interactions for current and future climates.

Context

The Field Phenotyping Platform (FIP) at ETH was established in 2015 to collect image time series of crops growing under realistic field conditions. The FIP's cable carrying system is capable of carrying a 90 kg sensor head [7]. The original sensor head, hereafter referred to as the FIP 1.0 head, was equipped with a red, green, and blue (RGB) camera and a Terrestrial Laser Scanner (TLS), among other sensors. Wheat field experiments were observed using FIP 1.0 over an eight-year period from 2015 to 2022, yielding six years of data collection, with 2015 and 2020 excluded due to incomplete measuring seasons (Figure 1). ~~Up to three times a week,~~ RGB images of all experimental units (so-called 'plots') were collected up to three times a week, and plant heights were measured simultaneously using either the TLS (2016, 2017) [8, 9] or drones (2018–2022) [10, 11, 12], two methods of height measurement that have been in good accordance with one another (R^2: 0.99 [12]). In 2023, the FIP 1.0 sensor head was replaced with a new, multi-view RGB sensor head. The described data set includes all RGB and height data collected in winter wheat experiments up to this replacement.

The area of approximately one hectare that the FIP can monitor is divided into six smaller parts (so-called 'lots') that are integrated into a crop rotation. The two FIP lots dedicated to winter wheat provide space for ~350 genotypes, replicated once per lot. For the first three years (2016–2018), the GABI-WHEAT [13] panel was grown as the genotype set. From 2019–2022, a subset of the GABI-WHEAT panel was grown in addition to other genotypes (Figure 2, green bars). The GABI-WHEAT panel consists of registered genotypes from different climatic regions of Europe [14, 13]. Genetic marker data and Multi-Environment Trial (MET) data from eight year-locations for GABI-WHEAT are publicly available.

The GABI-WHEAT panel was largely superseded by the Swiss breeding set in 2021 (Figure 2, ~~gray~~ orange bars). This new set primarily consists of eighth-generation (F8) breeding genotypes. ~~For~~ Private genetic marker data are available for the Swiss breeding set~~,~~ ~~genetic marker data exists but remains confidential.~~ . These can be shared directly through the breeders as part of a collaboration. The remaining genotypes, linked to specific projects such as Innovation in Variety Testing (INVITE), were present throughout all years but were generally only grown in a single year each (Figure 2, ~~in purple~~ purple bars). These genotypes currently lack available marker data.

In summary, by default the data set contains public genotypic data from ~300 genotypes over six years, allowing all the uses described in this data note. Through collaborations, this set can be expanded to ~800 genotypes.

Regular measurements with the FIP 1.0 head were accompanied by reference measurement campaigns as part of several projects. The heading date and senescence ratings were performed to investigate the relationships of senescence dynamics and diseases [15, 16, 17]. Yield measurements taken on the FIP field were combined with data from other locations to train phenomic prediction models [18]. The plant height measurements served as a basis to quantify the temperature response of wheat genotypes in the stem elongation phase [8, 9, 12]. The extracted plant height values demonstrated their usefulness in improving trait extraction methods from longitudinal data [19, 20, 21].

The images collected allowed to quantify canopy cover values [22] and examine their relationship to frost damage events [23] using Convolutional Neural Networks (CNNs). Using a combination of drone data and the high-resolution images the rows in the individual plots were identified [11]. In a small subset (375 images), the wheat heads were annotated and the data was integrated into the public global wheat head detection data set [24]. The image-based canopy cover values served as a test data set to evaluate the cultivar-specific extensions of the thermal time concept [25].

The culmination of these efforts has resulted in a unique, multi-dimensional data set. Dense image time series of diverse wheat genotypes are integrated with trait measurements, genetic markers, and environmental data. The data set has been designed to align with FAIR principles [26]:

- **Findable**: This publication and the Hugging Face data set card (https://doi.org/10.57967/hf/3191) provide detailed metadata and a comprehensive description of the data set's contents, making it discoverable to researchers.
- **Accessible**: The data is hosted on the Research Collection of ETH Zurich (https://doi.org/20.500.11850/697773), a reliable and openly accessible data storage.
- **Interoperable**: The use of the open-source Hugging Face datasets [27] package makes it easy to use and export to different formats. The data is fully MIAPPE v1.1 [28] conform. Given the shared genotypes the data set can be used to enhance the data by Gogna et al. [13] by 6 environments, to a total of 14 environments. The data set expands on existing sub-sets of the data already released that can be used as baseline approaches such as [8, 9, 19, 20, 21, 12, 25, 15, 16, 17].
- **Reusable**: The data is released under the ~~CC-BY 4.0 licence~~ ~~(~~CC0 1.0 Universal license (https://creativecommons.org/publicdomain/zero/1.0/), a permissive ~~licence~~ license that allows for further use of the data~~set~~.

Materials and Methods

Experimental Field Designs and Genotypes

All experiments were performed at the ETH research station of plant sciences in Lindau Eschikon, Switzerland (47.449 N, 8.682 E, 556 m a.s.l.). The soil characteristics were determined in 2015 (Eric

Compiled on: February 26, 2025.
Draft manuscript prepared by the author.

Schweizer AG, Thun, Switzerland). The soil type is eutric cambisol consisting of 21% clay and 21% silt with an organic matter content of 3.5% and pH 6.7. A crop rotation was implemented during and before the start of wheat experiments beginning with a year of soybean (*Glycine max* (L.) Merr.), then a year of buckwheat (*Fagopyrum esculentum* Moench) and finally wheat (*Triticum aestivum* L.). After preliminary crops were harvested, the soil was plowed and harrowed before wheat was drill-sown. The wheat was sown in 9 rows per plot with a row length of ~1.7 m, a row distance of 0.125 m and a sowing density of 370–400 plants m^{-2}.

A few days after sowing, herbicide (Herold SC, Bayer AG, Leverkusen, Germany) was applied to ensure weed-free plots. Several fungicides and insecticides were applied in spring to ensure healthy plants. The fertilizer was split into three doses (~ 1:3:1), one at tillering stage, one at start of stem elongation, and one after heading. Approximately 140 kg N, 90 kg P_2O_5, and 100 kg K_2O per ha were applied, depending on site-specific soil analysis. No irrigation was applied. The sowing and harvest dates for each year are provided in Table 1.

For 2016–2018, a GABI-WHEAT panel subset (consisting of ~300 European winter wheat cultivars from the GABI-WHEAT panel [14, 29]) was complemented by 35–52 Swiss winter wheat varieties of commercial importance. For 2019, a small subset of the GABI-WHEAT panel (54 genotypes) was grown alongside genotypes from other experiments (e.g., Swiss variety testing). In 2019, these other genotypes were grown on larger plots with a row length of 5.5 m while GABI-WHEAT genotypes were grown on the same size plots as in the other years. In 2021 and 2022, sets of F8 genotypes from the Swiss breeding program of Agroscope (Nyon, Switzerland) were grown. For an overview of genotype overlaps between years, see Figure 2.

For all years, an experimental design following the principles of good practice—replication, randomization, and blocking [30]—was chosen. The described panels of, on average, 350 genotypes per year were replicated once, each replication was randomized and grown on a different lot in the FIP area (Figure 1). Each replication was augmented with checks in a 3×3 block arrangement. For further details on the experimental design, see [8, 9, 12].

Image Data

The FIP system is divided into two independent parts, (1) the carrier system built and maintained by Spidercam (Spidercam GmbH, Feistritz im Rosental, Austria), and (2) the custom-built FIP 1.0 sensor head and control software [7].

Carrier: The Spidercam Cable-Suspended System

The carrier system, detailed in Kirchgessner et al. [7], uses four corner-mounted poles with pulleys. Cables connect these pulleys to winches, enabling 3-D movement of the FIP 1.0 sensor head. A working distance of 2–3 m from the canopy was maintained during measurements.

Sensors: The FIP 1.0 Imaging Head

The FIP 1.0 sensor head carried, amongst other sensors, a 21 MP full frame DSLR camera (EOS 5D Mark II, 35 mm lens (Canon Inc., Tokyo, Japan) [7]. The camera was triggered automatically via a custom MATLAB script (The Mathworks [31]). The images were mostly captured using auto white balance, auto exposure, an ISO of 100, an exposure time of 1/250 second (4 ms), and zero exposure bias value. The ground sampling distance is approximately 0.55 mm.

Image ~~registration~~Registration

The positions of the captured images of the plots change throughout the season due to inaccuracies in the camera carrier system and intentional height adjustments due to plant growth. To allow for consistent image feature extraction, the time series needed to be aligned using an image registration pipeline. Image registration transforms the images so that points that are at the same physical location in the real world are aligned to the same point in the image ~~plane~~planes. The used image registration pipeline consists of two steps: a deep learning-based feature matcher and a transformation estimation step. The feature matcher is used to find features that correspond to the same location between an image pair, which are then used to estimate the transformation between the images. The registration was performed between image pairs instead of the whole sequence directly, commonly referred to as image alignment.

To predict aligned polygons of the inner rows (Figure 1) for an image time series three individual steps were performed. First an initial reference polygon was aligned with a drone orthomosaic or prior data. Then subsequent aligned polygons were found based on this initial polygon or a previously aligned polygon. Finally, a single transformation between alignments and the inner plots was estimated and applied to all aligned polygons. The aligned result of a time series can be seen in Figure 3.

To find the initial reference polygon, the 3D-world-coordinates of the plot corners were first extracted from drone-based orthomosaics (2018–2022), from other projects [32, 10, 11, 33, 18, 12], or from extrapolations of these plot corners to earlier years (2016–2017). The 3D plot corners from this initial extraction process were matched to the best-fitting image in the image time series to find their 2D counterparts, which were used as the initial reference polygon.

Using the initial reference image and polygon, the initial image was matched with other images and the resulting transformation used to find the other aligned plot corners. Matching features between different time points in a crop season is challenging due to significant changes in conditions between images, such as varying lighting, plant growth and changing appearance of the plants. For this reason, a modified version of the deep learning LoFTR [34] feature matcher was fine-tuned to focus on consistent soil features, such as stones. Even these consistent objects slowly change their positions throughout the season or rapidly between strong precipitation events. Therefore, the transformations were estimated between image pairs and not the complete time series at a time. The estimated transformations between the image pairs are homographies based on the soil plane.

A prior alignment strategy employing Scale-invariant feature transform (SIFT) based feature matching required manual alignment of about 10,000 image pairs to fill gaps where the matcher failed. While suitable for smaller scale projects, this approach was deemed unviable for the complete data set. These manual alignments, created by choosing four point-pairs to find suitable homographies, were used to fine-tune the LoFTR feature matcher. The homographies correspond to the transformations between the common soil plane of the image pairs. This simplification was used to fine-tune the model to find matches solely in the soil plane, as features in the canopy are at incorrect positions due to not being on the homography plane. Since the canopy moves, sometimes significantly, between time-points, this reduces the number of incorrect matches that the feature matcher produces. The model was fine-tuned for 20 epochs with randomly cropped, rotated and distorted image pairs. These augmentations were applied to each image individually, adjusting the homographies accordingly. The augmentations were chosen such that the image pairs were always partially overlapping.

To verify the correctness of the matches and their homographies, several checks were employed. Basic checks, such as ensuring enough inliers, constraining the ratios of the side lengths and angles of the polygon spanned by the plot corners to their 3D counterparts, and other image-based checks were applied with high thresholds. The essential matrix was calculated, and the warped plot corners were compared to their closest point on the epipolar line. The essential matrix was used to triangulate the 3D points

of the plot corners, and they were compared to the original 3D-world-coordinates by checking the ratios and angles of the polygons spanned by the plot corners. These ratios were used to scale the predicted relative camera movement and compare it individually to the maximum possible movements in each axis. Finally, these predictions and checks were done twice with the image pair swapped and compared to each other. This check filtered cases where LoFTR predicted structured noise that corresponded to the identity homography between the image pairs. To filter outliers these steps were iteratively repeated, and the best prediction based on the checks was chosen.

When an image could not be matched to the initial image, it was iteratively matched with the closest found alignment, based on the number of transformations and then the date differences. Matches were used to find homographies using OpenCV [35], which were then used to warp the plot corners (Figure 1, 'Plot').

The complete process, except the initial 3D-world-coordinates extraction, was iteratively repeated by going from very tight to looser restrictions in the checks. The fine-tuned LoFTR model was bootstrapped, that is, further trained at each step by adding the trusted subset of its predictions that passed the checks in the previous step to the training set.

Finally after predicting all plot corners, a relative inner plot polygon was extracted to mitigate border effects and further correct the alignment. The seven inner rows of plants were detected based on segmented images showing plant and soil pixels. Then, plots were further rectified by rotating them step-wise (-1.5° to 1.5° in steps of 0.2°) to maximize the distance between the minimum and maximum numbers of plant pixels in image columns [25]. Inner plots were filtered if they did not contain the complete plot.

In total, the alignment and inner plot detection was successful for more than 95% of all images~~(151~~. The number of successfully aligned images per year can be seen in Table 2. Both the 160,772 original images and their corresponding 153,~~150 out of 158,891 images).~~ 022 inner plot cutouts are made available as part of this data set.

Image ~~preprocessing~~Preprocessing

The raw images were converted to PNG format, an accessible and lossless image format, using rawpy [36] with minimal post-processing, reducing resolution to half size, employing a linear demosaicing algorithm, and disabling auto adjustments in order to preserve the original sensor data with minimal artifacts or alterations. The inner plots were directly cut out of the pre-processed image by combining the plot and its relative inner plot transformation.

Reference Measurements

As part of several projects [8, 9, 15, 16, 18, 12], reference measurements such as grain yield and growth stage ratings were taken. Those traits can be divided into low-level traits (time series of traits that develop over time), intermediate traits (traits extracted from low-level traits that describe the growth dynamics over time [20, 19]) and target traits (observations that are usually targeted in breeding and agriculture, e.g., yield) (Table 7). Trait measurements may have been made at times different from the image captures. All measurements are scalars that correspond to a single measurement at a given time point during the season.

Low-level Traits

Plant height measurements were performed with a TLS (Focus 3D S 120, 905 nm laser, Faro Technologies Inc., Lake Mary, USA) for 2016 and 2017 on the same date as image acquisition [8]. From 2019–2022, drone RGB images [10, 11] were used to extract plant height estimations with Structure from Motion (SfM) [37]. FIP image collection dates and drone campaign dates were typically within days of each other but did not necessarily overlap. The accuracy of TLS and drone measurements to approximate manual plant height measurements were demonstrated in the respective publications (TLS [8]: R^2: 0.99, drone [37]: R^2: 0.96). The two methods are in good accordance with one another (R^2: 0.99, intercept: 0.057 m, slope: 1.0 [12]).

From images, canopy cover was extracted using a deep learning model described in Zenkl et al. [22], a model that reached a pixel accuracy of 0.945 on a FIP test set. The percentage of soil covered by plant parts was determined for the seven-row inner plot (Figure 1) as described in Tschurr et al. [38]. On the same seven-row inner plot, wheat head count estimations were determined for all dates from May to end of season. As the wheat head detection method, the winning model of the global wheat head challenge [24] (`https://github.com/ksnxr/GWC_solution`) was used, a model that achieved an average domain accuracy of 0.7 on a test set that included FIP images.

Senescence was assessed visually ~~, separately for the flag leaf and the whole~~ in 2016, 2017 and 2018 from approximately 20 days after flowering to full senescence for the central plot area canopy, following guidelines provided by Pask et al. [39]. ~~Flag leaf senescence and whole plot senescence~~ Plot senescence was scored according to Anderegg et al. [16] based on the portion of green leaf area on a scale from 0 to 10, equivalent to 0 to 100 %. To avoid bias, manual ratings were performed by the same expert in all three years.

The number of total measurements, as well as the minimum, maximum, and mean number of measurements per year for each low-level trait, can be seen in Table 3.

Intermediate Traits

Heading date was visually assessed as the date when 50% of the spikes were fully emerged from the flag leaf sheath [17] (BBCH 59, [40]). To ensure consistent ratings over time, heading date ratings were started at approximately BBCH 55 and continue to BBCH 61, with two to three rating events per week.

Final height was extracted from TLS or SfM plant height measurements using the Quarter of Maximum Elongation Rate (QMER) method described in Roth et al. [20]~~. —~~, which has a reported accuracy of close to 1.0 on simulated data. The number of measurements per year for intermediate and target traits can be seen in Table 4.

Target Traits

Yield for small plots in all years was estimated based on two rows of nine rows (row seven and eight). Ears within these two rows were hand-harvested, dried for at least 24 hours at 30 °C, and threshed using a stand thresher (Saatmeister Allesdrescher K35; Saatzucht Baumann, Germany). Yield for large plots in 2019 was determined with a combine harvester (Nursery- master Elite; Wintersteiger, Ried im Innkreis, Austria). Weight was determined using a scale, and water content with a Wile 55 moisture meter (Farmcomp Oy; FIN04360 Tuusula, Finland). Grain yield was mathematically normalized to 14 % water content. Grain protein content was determined using near-infrared transmission spectroscopy (InfratecTM 1241 Grain Analyzer; Foss, DK-3400 Hilleroed, Denmark).

~~Adjusted genotype means within year calculation~~

In 2019, plot sizes varied between experiments, which influences yield measurements. While large plot sizes deliver absolute estimates comparable with METs, in our experience, small plot sizes tend to overestimate yield per area. To compensate for this effect, adjusted genotype means (see next Section) of twelve common genotypes between the experiments with large and small plots were used to calculate a conversion factor. A linear regression with intercept zero estimated a conversion factor of 1.50 from large to small plots (R^2 of 0.47). This conversion factor was used to

transform all yield measurement estimates of small plots to the range of large plots, resulting in a new trait 'Grain yield (adjusted)'.

Adjusted Genotype Means within Year Calculation

All intermediate and target traits were processed to adjusted genotype means (Best Linear Unbiased Estimate (BLUE)) using a linear mixed model in SpATS~~for low-level traits [41] and ASReml-R [42] for intermediate and target traits~~,

$$y_{ijk} = m_c + g_i + p_{r(k)} + p_{c(k)} + S(r(k)), c(k)) + e_{ijk}\,, \qquad (1)$$

where y_{ijk} is the measured trait value for the ith genotype in the jth year for plot k in row $r(k)$ and column $c(k)$. m_c is a fixed effect marking check varieties ($m_c \in [0,1]$), g_i is a fixed genotype effect, $p_{r(k)}$ and $p_{c(k)}$ are ~~fixed~~ random spatial row and column effects, and e_{ijk} a spatially independent residual. ~~For SpATS,~~ $S(r(k)), c(k))$ is a spatial smooth surface in row and column direction as defined in [41]~~, whereas for ASReml-R, it is an auto-regression of order 1 in both row and column direction and two splines in row and column direction, $AR1_{r(k)} \times AR1_{c(k)} + Spl(r(k)) + Spl(c(k))$.~~.

Environmental Covariates

Air temperature, relative humidity, short wavelength solar irradiance, and soil temperature were measured at a local weather station in proximity to the experimental field above or below a grass strip [25]. Air temperature and relative humidity were measured 2 m and 0.1 m above ground, short wavelength solar irradiance 2 m above ground, and soil temperature 0.05 m below ground, respectively. Precipitation data were taken from a close-by Agrometeo weather station at Strickhof (<800 m, https://www.agrometeo.ch). Measurement gaps caused by technical issues (e.g., sensor failure) were filled with data from the Strickhof station if available and with data from a Meteoswiss station at the Zurich Airport (9.5 km, https://gate.meteoswiss.ch/idaweb/) otherwise. Overall, more than 97% of the data after gap filling originated from the local weather station or Strickhof weather station.

Genetic ~~marker data~~Marker Data

Genetic marker data (90k SNP array) for the GABI-WHEAT panel are publicly available [29]. The private marker data were created using a ~~45k~~ 25k SNP array. Two sets were compiled: A pure GABI-WHEAT marker data set, and an extended marker set consisting of overlapping marker from both public and private markers. Marker locations on the reference genome IWGSC RefSeq v1.0 were collected with InterMine [43] from https://urgi.versailles.inrae.fr and complemented with locations previously mapped with blastn from an earlier project [12].

SNPs in both sets were first filtered for minor allele frequency (5%) and missing values (5%). Genotypes in both sets were then tested for missing marker data with a missing rate of 1% (all passed, no genotype had to be removed). Finally, missing marker data (0.44% for GABI-WHEAT set, 0.54% for extended set) were imputed based on a k-nearest-neighbour implementation in R (scrime [44]) per chromosome. Markers were sorted by chromosome and location for later use in local connectivity models such as CNNs. Markers with ambiguous positions were left in the set but marked accordingly.

The resulting GABI-WHEAT genetic marker set includes 372 genotypes and 18'846 markers. The extended set includes 824 genotypes and 11'~~904~~ 943 markers. Based on the marker data, kinship matrices [45] were calculated according to Yang [46].

Compilation as Data ~~set~~Set

Train/Test Split According to Genetic Relatedness

The most interesting application of trait prediction approaches is to predict the performance of unseen genotypes in unseen years [47, 48]. The accuracy of such predictions typically depends on the relatedness of genotypes, usually assessed through cross-validation. However, cross-validation is computationally expensive for complex deep learning models. To address this issue, we propose an alternative approach that balances the train/test set using genetic relatedness [49]. This method theoretically yields performance close to the average of all cross-validation runs.

To implement this approach, we used the R package STPGA [50] to determine a test set complementing the training set with the algorithm 'D_{opt}' as suggested in [51]. The training set was further split into training and validation sets using the same method. We define four test sets to allow for specific evaluation of methods depending on their use. The test sets differ based on if their genotypes and environments occur in the train set (denoted as seen):

i. Test (P): Unseen plots with seen genotypes and seen environments (all years except 2019)
ii. Test (G): Unseen genotypes with seen environments
iii. Test (E): Unseen environment (2019) with seen genotypes
iv. Test (G and E): Unseen genotypes and unseen environment (2019)

To validate our balanced splitting approach, we also performed five-fold cross-validations, allowing for a quality check of the balanced splits. The splits were calculated separately for the pure GABI-WHEAT set and for the extended set~~, including~~. The extended set additionally includes F8 generation genotypes with private marker data~~in the latter~~. For the GABI-WHEAT set, this resulted in 262 genotypes in the training set, 24 in the validation set, and 30 in Test (G and E) test set (Figure 5). For the extended set, this resulted in ~~695~~ 747 genotypes in the training set, 30 in the validation set, and 30 in Test (G and E) test set.

Preparation as Hugging Face Data ~~set~~Set

All data was aggregated into a single table with a row for each plot containing the image sequence, the aligned image sequence, traits, environmental data, marker data, and additional metadata. The table was split using the aforementioned splits and converted to a single Hugging Face data sets DatasetDict using the schema shown in Table 8. The table and DatasetDict contain None values for completely missing entries. Missing data in sequences are absent.

Data Validation and Quality Control

Heritabilities of ~~low-level traits~~Low-level Traits

Heritability is a statistic used in breeding to quantify how much of a trait's variation is attributable to the examined genetic material, ranging from 0 to 1. For field phenotyping traits, heritability can indicate the quality of a trait, as one is interested in methods that extract highly genotype-specific values, i.e., traits with high heritability. To assess the quality of low-level traits, heritability was calculated for each time point by setting the genotype factor g_i in Equation 1 to random and estimating genetic and non-genetic variances, as described by Oakey et al. [52]. The resulting heritabilities (Figure 4) follow an expected temporal pattern: increasing with growth and decreasing towards the end of the growth phase. These findings align with previously reported values for the same data set, e.g. 0.77 for senescence traits (2016–2018) [16] and 0.61/0.59 for derived plant height traits (start/stop growth) [12]. Additionally, plant height measurements obtained using TLS have been shown to strongly correlate with drone-based height estimations (correlation

of 0.99) [12].

Heritabilities of ~~intermediate~~ Intermediate and ~~target traits~~Target Traits

To test for the quality of intermediate and target traits, heritability was calculated according to ~~Cullis~~ Oakey et al.~~[53]~~ [52] by setting the genotype factor g_i in Equation 1 to random and estimating genetic and non-genetic variances. The results (Table 5) are in accordance with values reported for the same data set before, e.g., 0.55 for grain yield in 2016–2018 [16], 0.97 for heading date in 2016–2018 [16], 0.84 for grain protein content in 2016 and 2017 [16], and 0.98 for final height in 2015–2018 [12]. ~~y~~ The quality of the public marker data set ~~fodata setershortGABI~~for the GABI-WHEAT panel was demonstrated in Gogna et al. by means of testing for genomic prediction ability [13]. The same quality check was performed on the data set presented herein, once with the public marker data from the GABI-WHEAT panel, once with the extended set that includes private marker data as well.

Three different GBLUP-based genomic prediction models were trained: One linear mixed model with simple main effect and identity variance (ID), one with simple main effect and diagonal variance (DIAG), and one with a simple main effect and random regression to environmental covariates [54]. As environmental covariates, metrics were based on the Standardized Precipitation and Evapotranspiration Index (SPEI) [55], Vapour Pressure Deficit (VPD), air temperature at 2.0 m above ground, and precipitation. For the SPEI and temperature, the mean, maximum and minimum value over the season were used, for VPD, the mean and maximum, and for precipitation, the sum of the values was used. All models were implemented in ASReml-R using code by [56].

~~The results suggest comparable accuracies to~~ Results are reported for the three scenarios unseen genotypes in unseen environment (Test (G and E), Table 9), unseen environments (Test (E), Table 10), and unseen genotypes (Test (G), Table 11). The test (G) correspons to the scenario reported in Gogna et al. [13]~~for final height, .~~ . Results show comparable performance for heading date and final height, superior performance for protein content, and inferior performance for grain yield (Table ~~9~~11). These results are in accordance to the heritabilities found for the traits (Table 5). ~~The random regression model could only outperform the two other modelsfor the trait heading date~~If comparing models, no notable difference in performance was found.

Genomic ~~prediction ability~~ Prediction Ability of ~~unseen multi-environment trial~~Unseen Multi-environment Trial

Gogna et al. have published a MET data set for yield, protein content, heading date and final height comprising eight environments (five locations and one to two years) for the GABI-WHEAT panel [13]. 312 of the measured genotypes overlap with the FIP 1.0 data set, 60 are unseen in the FIP 1.0 data set but marker data are available. The unseen genotypes (60) in unseen environments ~~[13]~~ (Test (G and E)) and seen genotypes (312) in unseen environments (Test (E)) were taken as independent test sets in new environments for a genomic prediction approach similar to the one described in the previous section. For the random regression model, hourly temperature, precipitation and relative humidity data for the German environments were extracted from the Climate Data Center (CDC) of the German Weather Service, those for the French environments from Meteo France (SYNOP, 3-hourly data only). These additional environmental covariate data and MET data are available in the data repository for convenience, but not part of the core data set (see folder 'MET_repository_clone').

For grain yield, the random regression model outperformed the other models ~~for both the GABI-WHEAT and extended marker set~~ (Table 9). For all other traits, no clear advantage of the random regression model over the other models was visible. Again, the results suggest comparable accuracies to Gogna et al. [13] for final height, heading date and protein content, and ~~slightly~~ inferior performance for grain yield (Table 9).

Re-use Potential and Limitations

In this work, we provide baselines for genomic prediction approaches, trait extractions from images, and subsequent trait dynamics modeling. Accordingly, we see the largest re-use potential of the presented data set for the development and evaluation of new modelling and prediction approaches in crop genomics and phenomics. The multi-faceted data set allows modelling approaches on various levels:

i. Genomic prediction approaches that include genotype-environment interactions: The presented data enhance the data by Gogna et al. [13] by 6 environments, totalling to 14 environments that are characterized by environmental covariates. The presented benchmark of a genomic prediction with random regressions to environmental covariates [54] provides a baseline that novel approaches can challenge.

ii. Modelling plant growth and development with longitudinal modelling approaches: The four low-level traits canopy cover, plant height, wheat head count and senescence cover the full growing season of winter wheat in 6 environments that are characterized by environmental covariates. Baseline approaches for plant height growth modelling [8, 9, 19, 20, 21, 12], canopy cover growth modelling [25] and senescence dynamics modelling [15, 16, 17] for subsets of the presented data exist.

iii. Image-based phenomic predictions and combined phenomic and genomic prediction approaches: The dense time series of images allow training and analysing end-to-end modelling approaches (e.g., deep learning based) that predict target traits such as yield based on images.

While the data set opens up the possibility of analysing HTFP data to a wide audience, it also has its inherent limitations that should be taken into account if working with it:

- The immobility of the FIP restricts the data set to only one location.
- Field-based data collection introduces various sources of errors that one must consider in analysis (see e.g. [20] for a discussion).
- Yield measurements in the FIP and hence this data set are more prone to error than in METs.
- Annotation at the image level only requires further annotation effort if semantic segmentation or object detection methods are targeted.
- While the aligned image time series provide extensive opportunities to analyze growth dynamics, this kind of highly preprocessed image data is rare and therefore interoperability with other data sources is yet limited.

Examples

To run the following examples the Huggingface *datasets* [27] library is required. The examples were run using version *3.3.2*.

Example 1: Basic access to data set via hugging face datasets package

```
import datasets
fip1 = datasets.load_dataset("mikeboss/FIP1")
fip1_train = fip1["train"]
print(fip1)
print(fip1_train)
# Output:
DatasetDict({
    train: Dataset({
        features: ['plot_uid', 'yearsite_uid', 'crop_type', 'experiment_number', 'plot_number', 'dates', 'times', 'sowing_date', 'harves[illegible]
Dataset({
    features: ['plot_uid', 'yearsite_uid', 'crop_type', 'experin[illegible]le', 'longitud[illegible]
    num_rows: 2930
})
    validation: ...
    test_plot: ...
    test_genotype: ...
    test_environment: ...
    test_genotype_environment: ...
})
```

Listing 1. Example 1

Example 2: Load aligned inner plot cutouts as stacked ~~pytorch tensor~~numpy array

```
fip1 = fip1.select_columns("inner_plot_images")
fip1 = fip1.cast_column("inner_plot_images", datasets.
    Sequence(datasets.Image()))
fip1 = fip1.with_format("pt")
fip1 = fip1.with_format("numpy")
print(fip1["train"][0]["inner_plot_images"].shape)
# Output:
torch.Size([26, 3, 640, 960])
(35, 640, 960, 3)
```

Listing 2. Example 2

Example 3: Access low-level trait time series

```
print(fip1["train"][0]["height_values"])
# Output:
[0.3284, 0.3833, 0.4375, 0.4749, 0.5024, 0.5396, 0.582, 0.7456, 0.9165, 0.9385, 1.018, 1.13, 1.2, 1.242, 1.268, 1.37, 1.369, 1.372, 1.38[illegible]
[np.float16(0.2231), np.float16(0.2725), np.float16(0.3188), ...]
```

Listing 3. Example 3

Example 4: Access specific target traits

```
fip1 = fip1.select_columns(["yield_value", "protein_value", "he[illegible]
fip1 = fip1.select_columns(["yield_value", "protein_value", "height_final_value", "heading_value"])
print(fip1["train"][0])
# Output:
{'yield_value': 12.04, 'protein_value': 13.61, 'height_final_value': 1.346, 'heading_date_value': 1576.0}
{'yield_value': np.float16(8.64), 'protein_value': np.float16(13.74), 'height_final_value': np.float16(1.062), 'heading_value': np.float[illegible]
```

Listing 4. Example 4

Example 5: Access marker data

```
fip1 = fip1.with_format("numpy")
print(fip1["train"][0]["marker_biallelic_codes"])
print(fip1["train"][0]["marker_metadata_strings"])
# Output:
[2 2 2 ... 2 2 2]
['0000000001:chr1A:1145398:1145498:BS00000713_51_T_C'
 '0000000003:chr1A:1174887:1174987:
    Excalibur_c10657_1280_C_T'
 '0000000004:chr1A:1176337:1176337:
    wsnp_Ex_c10657_17376086_C_T' ...
 'wsnp_bf474966A_Ta_2_1_T_C' 'wsnp_bm138650D_Ta_2_2_G_A'
 'wsnp_cd454041D_Ta_2_1_C_T']
```

Listing 5. Example 5

Example 6: Access environmental data in January

```
fip1 = fip1.select_columns(
    ["temperature_air_10cm_values", "
     temperature_air_10cm_dates"]
)
fip1 = fip1.map(
    lambda values, dates: {
        "temperature_air_10cm_values_january": [
            value for value, date in zip(values, dates,
     strict=True) if date.month == 1
        ]
    },
    input_columns=["temperature_air_10cm_values", "
     temperature_air_10cm_dates"],
)
print(fip1["train"][0]["temperature_air_10cm_values_january
    "][:10])
# Output:
[2.6, 2.7, 2.8, 2.4, 0.6, 0.6, 1.3, 2.3, 2.6, 2.9]
[np.float16(2.6), np.float16(2.7), np.float16(2.8), ...]
```

Listing 6. Example 6

Availability of Source Code and Requirements

The code to recreate the derived data and the data set is publicly available in three repositories, namely the *FIP 1.0 Data Set - Traits*, *fip1-alignment*, and *fip1-dataset* repositories.

The complete process to create the data set involves extracting trait data from the raw data using the *FIP 1.0 Data Set - Traits* repository, then aligning the image time-series using the *fip1-alignment* repository, and finally aggregating the derived data into the final data set using the *fip1-dataset* repository.

In addition, the data set can be recreated using the *fip1-dataset* repository from the derived data that is freely available in the ETH research collection.

Trait ~~data compilation~~Data Compilation

Project name: FIP 1.0 Data Set - Traits
Project home page: `https://gitlab.ethz.ch/crop_phenotyping/fip-1.0-data-set-traits`
Operating system(s): ~~e.g.~~ Platform independent
Programming language: R, Python
License: GNU GPL v3

Image ~~data alignment~~Data Alignment

Project name: fip1-alignment
Project home page: `https://gitlab.ethz.ch/crop_phenotyping/fip1-alignment`
Operating system(s): ~~e.g.~~ Platform independent
Programming language: Python
License: GNU GPL v3

Data ~~set compilation~~Set Compilation

Project name: fip1-dataset
Project home page: `https://gitlab.ethz.ch/crop_phenotyping/fip1-dataset`
Operating system(s): ~~e.g.~~ Platform independent

Programming language: Python
License: GNU GPL v3

Data Availability

- Data Repository: http://doi.org/20.500.11850/697773
- Hugging Face Data set: https://huggingface.co/datasets/mikeboss/FIP1
- Public GABI marker data repository (also integrated in main Data Repository and Hugging Face Data set): https://doi.org/10.5061/dryad.n02v6wwzc
- Private Agroscope marker data repository: Confidential (Contact: Boulos Chalhoub, boulos.chalhoub@agroscope.admin.ch). This repository contains marker data (Illumina Infinium 25k array) from eight generation (F8) breeding lines that are unregistered and property of Agroscope. Access can be requested by stating the intended purpose of use and the willingness to sign a material transfer agreement (MTA).

Declarations

Consent for Publication

Not applicable.

Competing Interests

The author(s) declare that they have no competing interests.

Funding

A.W. discloses support for the research of this work from Swiss National Science Foundation [grant number 169542 and 200756]. L.R. discloses support for the research of this work from Swiss Data Science Center [grant number PHENO-MINE C21-04].

Author's Contributions

Lukas Roth: Conceptualization, Methodology, Software, Validation, Formal analysis, Investigation, Data Curation, Writing - Original Draft, Visualization, Supervision, Funding acquisition. Mike Boss: Conceptualization, Methodology, Software, Validation, Formal analysis, Investigation, Data Curation, Writing - Original Draft, Visualization. Norbert Kirchgessner: Conceptualization, Methodology, Software, Validation, Formal analysis, Investigation, Data Curation, Writing - Original Draft, Visualization. Helge Aasen: Investigation, Supervision. Brenda Patricia Aguirre-Cuellar: Investigation. Price Pius Atuah Akiina: Investigation. Jonas Anderegg: Methodology, Data Curation, Investigation. Joaquin Gajardo Castillo: Software, Data Curation. Xiaoran Chen: Investigation. Simon Corrado: Investigation. Krzysztof Cybulski: Software, Data Curation. Beat Keller: Investigation, Supervision. Stefan Göbel Kortstee: Investigation. Lukas Kronenberg: Methodology, Data Curation, Investigation. Frank Liebisch: Investigation, Supervision. Paraskevi Nousi: Investigation. Corina Oppliger: Investigation. Gregor Perich: Investigation. Johannes Pfeifer: Investigation. Kang Yu: Investigation. Nicola Storni: Software, Data Curation, Investigation. Flavian Tschurr: Software, Data Curation, Investigation. Michele Volpi: Investigation, Supervision. Simon Treier: Investigation, Data Curation. Hansueli Zellweger: Investigation. Olivia Zumsteg: Investigation. Andreas Hund: Conceptualization, Methodology, ~~Writing – Review & Editing~~Writing - Review & Editing, Supervision, Project administration. Achim Walter: Conceptualization, ~~Writing – Review & Editing~~Writing - Review & Editing, Supervision, Project administration, Funding acquisition.

Acknowledgements

Not applicable.

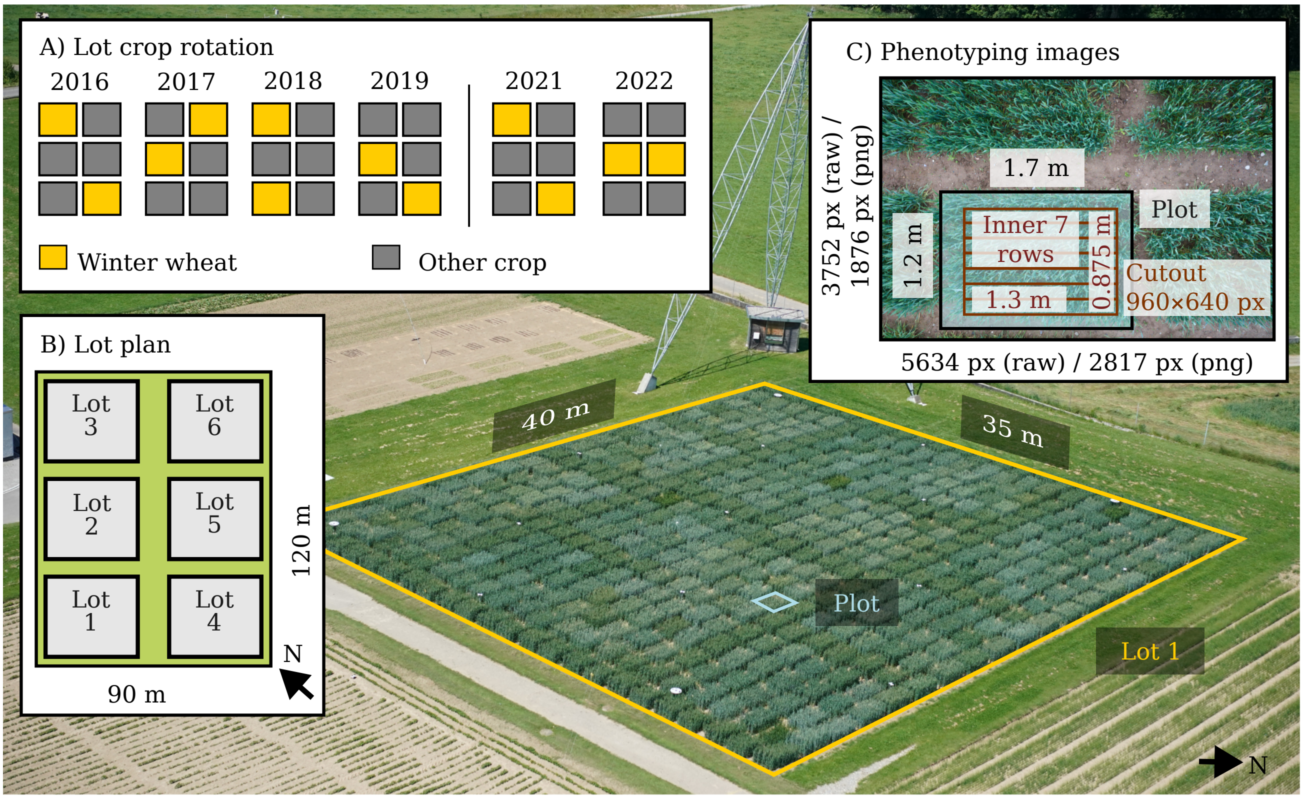

Figure 1. The data set source: 12 wheat lots in 6 years (2016–2022) with >350 wheat plots each, resulting in >4,000 plots from which >160,000 images were taken. The background image shows the Field Phenotyping Platform (FIP) lot 1 with wheat plots. All wheat lots were integrated in a regular crop rotation with other crops (A) according to a permanent lot plan (B). Images taken with the FIP show one complete plot each, the positions of the complete plot and the inner 7 rows are annotated (C).

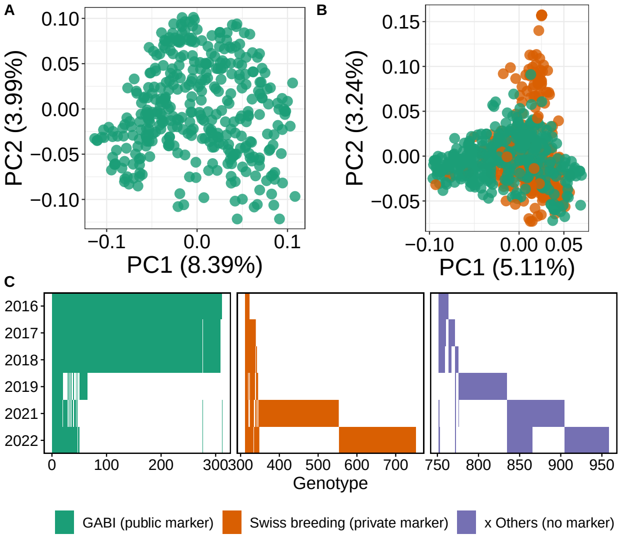

Figure 2. The examined genotype sets with their genetic relatedness visualized in SNP-marker based principal component analysis based on public GABI-WHEAT markers (A) and public and private markers combined (B), and year of cultivation of genotypes (C).

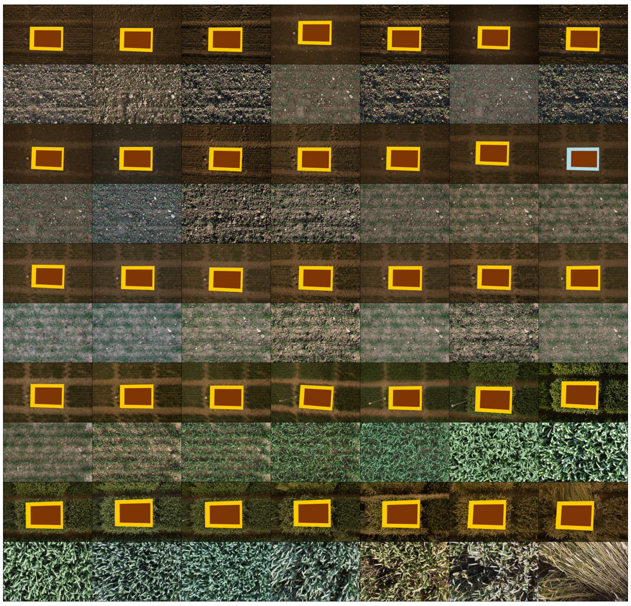

Figure 3. An image time series of a plot over one season with the aligned inner plot cutouts in each second row. The initial alignment polygon, shown in light blue, is based on alignment with a drone orthophoto. All other alignment polygons, shown in orange, are transformed from the initial polygon or another aligned polygon. The inner plot polygons, shown in brown, are based on a single transform from an aligned polygon to the inner rows of the plot. Images are adjusted for visibility.

Table 1. Sowing and harvest dates

Year	Sowing Date	Harvest Date
2016	2015-10-13	2016-07-27
2017	2016-11-01	2017-07-19
2018	2017-11-02	2018-07-14
2019	2018-10-17	2019-07-23
2021	2020-10-21	2021-07-29
2022	2021-11-25	2022-07-19

Table 2. Data set sizes for original images and key characteristicsaligned images per year. Numbers describe absolute sizesFor each category, i.e., how many plots and genotypes were examined in a specific year, and how many the number of this plotsimages and minimum / time series maximum / mean time-series length are annotated with target traits yield, grain protein content, heading date, and final heightgiven.

Year	Plots	Original images				Aligned images			
		# Images	Min	Max	Mean	# Images	Min	Max	Mean
2016	710	26237	35	39	36.95	25566	26	39	36.01
2017	756	19717	23	30	26.08	14534	9	29	19.22
2018	756	21642	26	31	28.63	21266	12	31	28.13
2019	252	7451	28	34	29.57	6445	20	34	25.57
2021	792	48451	58	63	61.17	48285	58	63	60.97
2022	792	37274	44	51	47.06	36926	44	51	46.62
Total	4058	160772	23	63	39.62	153022	9	63	37.71

Table 3. Time-series data set sizes for low-level traits. For each low-level trait the number of measurements and minimum / maximum / mean time-series length are given.

Year	Plots	Canopy Cover				Plant Height				Wheat head count				Senescence rating			
		#	Min	Max	Mean	#	Min	Max	Mean	#	Min	Max	Mean	#	Min	Max	Mean
2016	710	710	27	39	36.00	710	22	22	22.00	710	1	11	9.51	703	0	9	8.78
2017	756	756	9	24	18.52	756	21	21	21.00	756	1	18	9.77	756	1	10	9.77
2018	756	756	12	27	21.50	756	36	43	39.72	755	0	18	10.35	756	12	12	12.00
2019	252	252	8	25	21.89	252	38	46	44.55	228	0	10	3.57	–	–	–	–
2021	792	792	55	61	59.21	792	45	48	46.56	792	30	36	33.96	–	–	–	–
2022	792	792	31	41	38.46	792	42	45	42.53	792	12	24	21.55	–	–	–	–
Total	4058	4058	8	61	34.18	4058	21	48	35.13	4057	0	36	16.47	2215	0	12	5.59

Table 4. Data set sizes and key characteristics for genotypes as well as intermediate and target traits. Numbers describe absolute sizes, i.e., how many plots and genotypes were examined in a specific year, and how many of this plots/time series are annotated with target traits grain yield, grain protein content, heading date, and final height.

Year	Plots	Genotypes	Grain yield / Grain yield (adjusted)	Protein content	Heading date	Final height
2016	710	335	710	685	710	710
2017	756	352	754	0	377	756
2018	756	353	756	674	369	756
2019	252	90	248	144	177	252
2021	792	346	661	781	453	792
2022	792	373	744	792	719	792
Total	4058	904	3873	3076	2805	4058

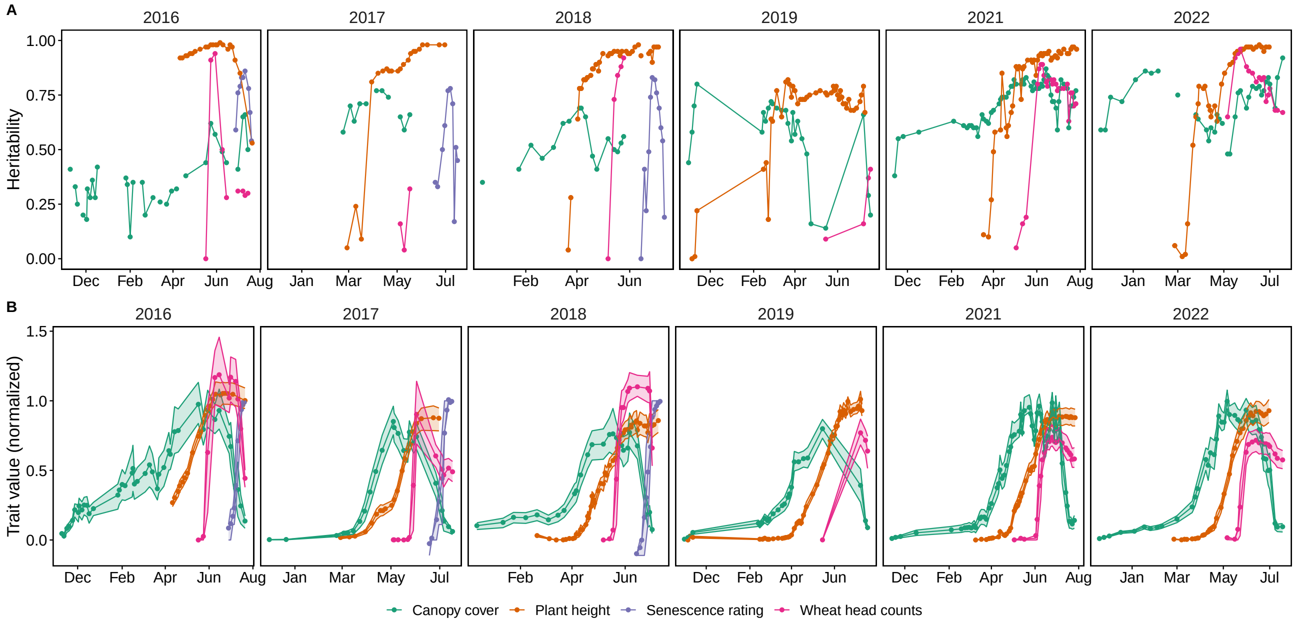

Figure 4. Heritability of the four low-level traits per point in time for all years (2016–2022) (A) and normalized measured trait values (B). Indicated are means (points) and the 25% and 75% percentile percentiles (areas).

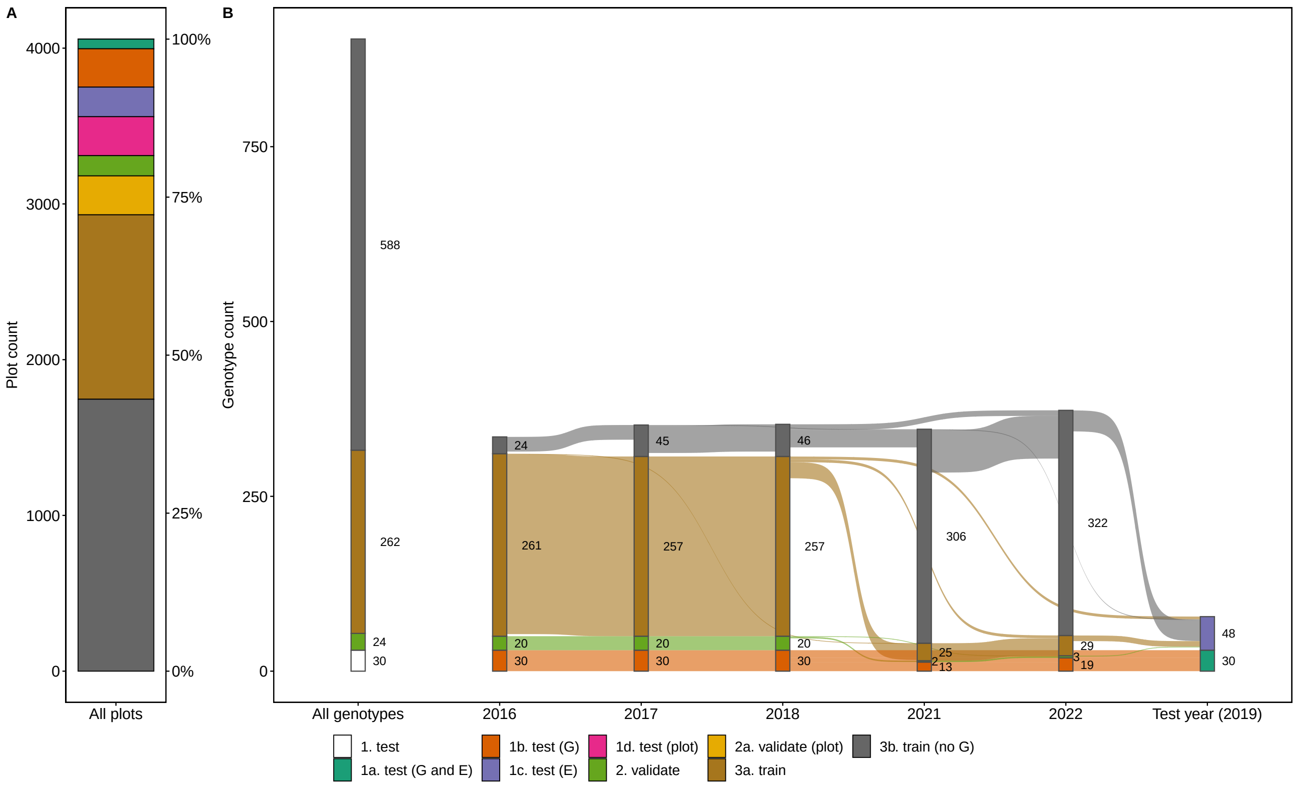

Figure 5. Training, validation and test splits for the GABI-WHEAT marker data based genotype set. The test set includes four subsets, unseen genotypes (G), unseen environments (E), unseen genotypes in unseen environments (G and E), and seen genotypes in seen years on unseen plots (plots). The validation set includes two subsets, unseen genotypes (default) and seen genotypes on unseen plots (plots). The splits are shown based on plots (A) and based on genotype count (B), genotypes occurring in different sets are reported at the lowest level (train < validation < test).

Table 5. Heritabilities (H^2) of intermediate and target traits for all years.

Trait	Heritability (H^2)					
	2016	2017	2018	2019	2021	2022
Grain yield	0.58	0.36	0.33	0.79	0.41	0.48
Grain yield (adjusted)	(s)	(s)	(s)	0.83	(s)	(s)
Protein content	0.82	-	0.81	0.80	0.55	0.86
Heading date	0.96	N/A	0.90	0.89	0.81	0.93
Final height	0.97	0.98	0.97	0.93	0.96	0.97

N/A: Unreplicated measurement, heritability not available
-: No measurements in this year
Grain yield (adjusted): Plot size adjusted yield ($\text{yield}_{5\text{ m}} = \text{yield}_{1\text{ m}} / 1.50$)
(s): All plot same size, see results for "Grain yield"

Table 6. Variance decomposition results of intermediate and target traits for all years.

Trait	Component	Percentage of total variance					
		2016	2017	2018	2019	2021	2022
Grain yield	Genotype	8%	11%	1%	1%	18%	3%
	Spatial	81%	52%	94%	98%	41%	91%
	Residual	11%	37%	5%	1%	41%	6%
Grain yield (adjusted)	Genotype	(s)	(s)	(s)	28%	(s)	(s)
	Spatial	(s)	(s)	(s)	59%	(s)	(s)
	Residual	(s)	(s)	(s)	13%	(s)	(s)
Protein content	Genotype	1%	-	2%	11%	7%	6%
	Spatial	98%	-	97%	85%	83%	92%
	Residual	0%	-	1%	4%	11%	2%
Heading date	Genotype	61%	N/A	58%	57%	77%	35%
	Spatial	34%	N/A	36%	29%	2%	61%
	Residual	4%	N/A	6%	14%	20%	4%
Final height	Genotype	60%	87%	5%	19%	6%	12%
	Spatial	36%	9%	95%	77%	94%	88%
	Residual	3%	3%	0%	3%	0%	1%

N/A: Unreplicated measurement, variance components not available
-: No measurements in this year
Grain yield (adjusted): Plot size adjusted yield ($yield_{5\,m} = yield_{1\,m} / 1.50$)
(s): All plots same size, see results for "Grain yield"

Table 7. Measured traits that were used to annotate image time series. Label refers to the name used in the data set for the trait.

Trait	Unit	Label	Description
**Low-level traits**			
Canopy cover	(0...1)	canopy_cover	Soil coverage by plants from a nadir view
Plant height	m	height	Distance top of soil to top of canopy
Wheat head count	Count	spike_count	Number of visible wheat spikes
Senescence rating	(0...10)	senescence	End-of-season leaf decay ratings
**Intermediate traits**			
Heading	Date	heading	Date when 50% of wheat spikes visible
Final height	m	height_final	Plant height at end-of-season
**Target traits**			
Grain yield	t/ha	yield	Total weight of harvested grains
Grain yield (adjusted)	t/ha	yield_adjusted	Plot size adjusted yield
Protein content	%	protein	Grain protein content

Table 8. A schema of the data set ~~similar~~ equivalent to the ~~actual schema of the~~ Hugging Face data set schema. Sequence indicates a variable length of values while Array2D indicates a ~~consistent~~ fixed shape of the data.

Field Name	Type
**identifiers**	
plot_uid	string
yearsite_uid	string
crop_type	string
experiment_number	uint8
plot_number	int16
**location**	
range	uint8
row	uint8
lot	uint8
latitude	float64
longitude	float64
spatial_check	int32
**dates**	
sowing_date	date32
harvest_date	date32
harvest_year	uint16
**images**	
images	Sequence[string]
image_dates	Sequence[date32]
image_times	Sequence[time32[s]]
**alignments**	
alignment_plot_soil_polygons	Sequence[Array2D[float16]]
alignment_num_steps	Sequence[uint8]
alignment_dates	Sequence[date32]
alignment_times	Sequence[time32[s]]
alignment_initial_date	date32
alignment_inner_plot_transform	Array2D[float16]
inner_plot_images	Sequence[string]
image_inner_plot_transforms	Sequence[Array2D[float16]]
**markers**	
genotype_id	string
marker_biallelic_codes	Sequence[uint8]
marker_metadata_strings	Sequence[string]
**low-level traits**	
canopy_cover_values	Sequence[float16]
canopy_cover_dates	Sequence[date32]
canopy_cover_trait_ids	Sequence[uint8]
canopy_cover_trait_name	Sequence[string]
canopy_cover_method_ids	Sequence[uint16]
canopy_cover_method_name	Sequence[string]
canopy_cover_si_unit	string
canopy_cover_responsible	string
height_values	Sequence[float16]
height_dates	Sequence[date32]
height_trait_ids	Sequence[uint8]
height_trait_name	Sequence[string]
height_method_ids	Sequence[uint16]
height_method_name	Sequence[string]
height_si_unit	string
height_responsible	string
spike_count_values	Sequence[float16]
spike_count_dates	Sequence[date32]
spike_count_trait_ids	Sequence[uint8]
spike_count_trait_name	Sequence[string]
spike_count_method_ids	Sequence[uint16]
spike_count_method_name	Sequence[string]
spike_count_si_unit	string
spike_count_responsible	string
senescence_values	Sequence[float16]
senescence_dates	Sequence[date32]
senescence_trait_ids	Sequence[uint8]
senescence_trait_name	Sequence[string]
senescence_method_ids	Sequence[uint16]
senescence_method_name	Sequence[string]
senescence_si_unit	string
senescence_responsible	string
**intermediate traits**	
heading_value	float16
heading_date	date32
heading_blue	float16
heading_heritability	float16
heading_trait_id	uint8
heading_trait_name	Sequence[string]
heading_method_id	uint16
heading_method_name	Sequence[string]
heading_si_unit	string
heading_responsible	string
height_final_value	float16
height_final_date	date32
height_final_blue	float16
height_final_heritability	float16
height_final_trait_id	uint8
height_final_trait_name	string
height_final_method_id	uint8
height_final_method_name	string
height_final_si_unit	string
height_final_responsible	string
**target traits**	
yield_value	float16
yield_date	date32
yield_blue	float16
yield_heritability	float16
yield_trait_id	uint8
yield_trait_name	string
yield_method_id	uint16
yield_method_name	string
yield_si_unit	string
yield_responsible	string
yield_adjusted_value	float16
yield_adjusted_date	date32
yield_adjusted_blue	float16
yield_adjusted_heritability	float16
yield_adjusted_trait_id	uint8
yield_adjusted_trait_name	string
yield_adjusted_method_id	uint16
yield_adjusted_method_name	string
yield_adjusted_si_unit	string
yield_adjusted_responsible	string
protein_value	float16
protein_date	date32
protein_blue	float16
protein_heritability	float16
protein_trait_id	uint8
protein_trait_name	string
protein_method_id	uint16
protein_method_name	string
protein_si_unit	string
protein_responsible	string
**environment**	
temperature_air_10cm_values	Sequence[float16]
temperature_air_10cm_dates	Sequence[date32]
temperature_air_10cm_times	Sequence[time32[s]]
temperature_air_200cm_values	Sequence[float16]
temperature_air_200cm_dates	Sequence[date32]
temperature_air_200cm_times	Sequence[time32[s]]
temperature_soil_5cm_values	Sequence[float16]
temperature_soil_5cm_dates	Sequence[date32]
temperature_soil_5cm_times	Sequence[time32[s]]
humidity_air_10cm_values	Sequence[float16]
humidity_air_10cm_dates	Sequence[date32]
humidity_air_10cm_times	Sequence[time32[s]]
humidity_air_200cm_values	Sequence[float16]
humidity_air_200cm_dates	Sequence[date32]
humidity_air_200cm_times	Sequence[time32[s]]
precipitation_200cm_values	Sequence[float16]
precipitation_200cm_dates	Sequence[date32]
precipitation_200cm_times	Sequence[time32[s]]
irradiance_solar_200cm_values	Sequence[float16]
irradiance_solar_200cm_dates	Sequence[date32]
irradiance_solar_200cm_times	Sequence[time32[s]]

Table 9. Genomic prediction accuracy (correlation) and bias (RMSE) of intermediate and target traits for unseen genotypes in unseen environments (Test (G and E)).

Split set	Trait	ID			DIAG			RREG			ID			DIAG			RREG		
		Correlation									RMSE								
		B	CV	MET	B	CV	MET	B	CV	MET	B	CV	MET	B	CV	MET	B	CV	MET
GABI	Grain Yield	0.19	0.35	0.22	0.18	0.34	0.24	0.16	0.33	0.34	1.25	1.32	1.45	1.25	1.33	1.45	1.52	1.67	1.09
GABI	Grain Yield (adjusted)	0.16	0.34	0.21	0.16	0.34	0.23	0.15	0.33	0.31	1.57	1.67	2.52	1.57	1.67	2.51	1.86	1.99	3.1
GABI	Protein Content	0.36	0.46	0.46	0.34	0.45	0.46	0.33	0.43	0.46	1.11	1.13	1.23	1.13	1.14	1.23	0.65	0.67	1.55
GABI	Heading Date	0.59	0.55	0.67	0.59	0.55	0.67	0.59	0.55	0.67	6.57	7.31	5.77	6.54	7.28	5.77	5.35	6.08	5.86
GABI	Plant Height	0.8	0.75	0.81	0.8	0.74	0.81	0.57	0.61	0.75	0.06	0.07	0.08	0.06	0.06	0.08	0.18	0.17	0.17
Extended	Grain Yield	0.17	0.32	0.27	0.17	0.32	0.28	0.17	0.31	0.41	1.27	1.27	1.42	1.26	1.27	1.42	1.51	1.59	1.1
Extended	Grain Yield (adjusted)	0.23	0.31	0.27	0.23	0.31	0.27	0.23	0.3	0.37	1.66	1.86	2.51	1.66	1.86	2.5	1.92	2.13	3.03
Extended	Protein Content	0.37	0.64	0.52	0.37	0.64	0.53	0.36	0.63	0.52	1.12	1.06	1.25	1.12	1.07	1.25	0.81	0.79	1.55
Extended	Heading Date	0.6	0.53	0.66	0.61	0.57	0.68	0.62	0.55	0.64	6.95	6.65	5.73	6.95	6.66	5.71	5.59	5.3	5.88
Extended	Plant Height	0.8	0.76	0.82	0.8	0.78	0.81	N/A	0.65	0.8	0.05	0.05	0.08	0.05	0.05	0.08	N/A	0.34	0.17

ID: Identical variances; DIAG: Varying variances per year; RREG: Random regression to environmental covariates; ~~NA~~N/A: Failed convergence ~~of models~~
Test set: B: Balanced FIP 1.0 data set; CV: 5-fold cross-validation on FIP 1.0 data set; MET: Unseen multi-environment trial data set

Table 10. Genomic prediction accuracy (correlation) and bias (RMSE) of intermediate and target traits for seen genotypes in unseen environments (Test (E)).

Split set	Trait	ID			DIAG			RREG			ID			DIAG			RREG		
		Correlation									RMSE								
		B	CV	MET	B	CV	MET	B	CV	MET	B	CV	MET	B	CV	MET	B	CV	MET
GABI	Grain Yield	0.73	0.36	0.26	0.74	0.35	0.27	0.67	0.3	0.33	1.52	1.31	1.53	1.53	1.32	1.53	1.97	1.68	1.11
GABI	Grain Yield (adjusted)	0.76	0.37	0.26	0.76	0.36	0.27	0.69	0.31	0.31	1.97	1.74	2.4	1.98	1.74	2.4	2.31	2.04	2.99
GABI	Protein Content	0.92	0.79	0.52	0.93	0.78	0.52	0.92	0.77	0.52	0.83	0.95	1.37	0.84	0.96	1.36	0.34	0.49	1.74
GABI	Heading Date	0.94	0.9	0.9	0.94	0.9	0.9	0.94	0.9	0.89	7.22	7.05	5.06	7.2	7.04	5.06	5.97	5.78	5.16
GABI	Plant Height	0.96	0.97	0.92	0.95	0.97	0.92	0.64	0.87	0.72	0.04	0.03	0.08	0.04	0.03	0.08	0.14	0.15	0.18
Extended	Grain Yield	0.61	0.38	0.29	0.62	0.39	0.3	0.59	0.37	0.37	1.19	1.23	1.52	1.18	1.22	1.52	1.55	1.51	1.15
Extended	Grain Yield (adjusted)	0.69	0.46	0.29	0.7	0.47	0.29	0.68	0.45	0.35	1.85	1.74	2.38	1.85	1.74	2.38	2.14	2	2.91
Extended	Protein Content	0.87	0.83	0.53	0.87	0.83	0.53	0.86	0.81	0.53	0.9	0.95	1.38	0.91	0.96	1.38	0.55	0.58	1.72
Extended	Heading Date	0.89	0.89	0.9	0.89	0.89	0.9	0.89	0.89	0.89	6.55	6.56	5.04	6.56	6.56	5.04	5.15	5.14	5.21
Extended	Plant Height	0.94	0.95	0.92	0.94	0.95	0.92	N/A	0.87	0.91	0.04	0.04	0.08	0.04	0.04	0.08	N/A	0.34	0.16

ID: Identical variances; DIAG: Varying variances per year; RREG: Random regression to environmental covariates; N/A: Failed convergence
Test set: B: Balanced FIP 1.0 data set; CV: 5-fold cross-validation on FIP 1.0 data set; MET: Unseen multi-environment trial data set

Table 11. Genomic prediction accuracy (correlation) and bias (RMSE) of intermediate and target traits for unseen genotypes in seen environments (Test (G)).

Split set	Trait	ID		DIAG		RREG		ID		DIAG		RREG	
		Correlation						RMSE					
		B	CV	B	CV	B	CV	B	CV	B	CV	B	CV
GABI	Grain Yield	0.45	0.44	0.44	0.42	0.45	0.44	1.04	1	1.04	1	1.04	1
GABI	Grain Yield (adjusted)	0.45	0.44	0.44	0.42	0.45	0.44	0.69	0.66	0.69	0.66	0.69	0.66
GABI	Protein Content	0.61	0.58	0.62	0.58	0.61	0.59	0.75	0.88	0.73	0.87	0.75	0.87
GABI	Heading Date	0.59	0.53	0.59	0.53	0.59	0.53	2.08	2.24	2.1	2.24	2.08	2.24
GABI	Plant Height	0.83	0.7	0.83	0.71	0.76	0.69	0.07	0.08	0.07	0.08	0.08	0.08
Extended	Grain Yield	0.48	0.37	0.47	0.39	0.48	0.37	0.99	1.04	0.99	1.04	0.99	1.04
Extended	Grain Yield (adjusted)	0.48	0.37	0.47	0.39	0.48	0.37	0.65	0.69	0.65	0.69	0.65	0.69
Extended	Protein Content	0.7	0.79	0.71	0.79	0.7	0.79	0.68	0.69	0.68	0.7	0.68	0.69
Extended	Heading Date	0.63	0.53	0.65	0.58	0.64	0.53	1.93	2.13	1.88	2.05	1.91	2.11
Extended	Plant Height	0.82	0.67	0.83	0.71	N/A	0.63	0.06	0.07	0.06	0.07	N/A	0.07

ID: Identical variances; DIAG: Varying variances per year; RREG: Random regression to environmental covariates; N/A: Failed convergence
Test set: B: Balanced FIP 1.0 data set; CV: 5-fold cross-validation on FIP 1.0 data set

References

1. Tilman D, Balzer C, Hill J, Befort BL. Global Food Demand and the Sustainable Intensification of Agriculture. Proceedings of the National Academy of Sciences 2011;108(50):20260–20264.
2. Pretty J, Sutherland WJ, Ashby J, Auburn J, Baulcombe D, Bell M, et al. The Top 100 Questions of Importance to the Future of Global Agriculture. International Journal of Agricultural Sustainability 2010;8(4):219–236.
3. Martre P, Dueri S, Guarin JR, Ewert F, Webber H, Calderini D, et al. Global needs for nitrogen fertilizer to improve wheat yield under climate change. Nature Plants 2024;10(7):1081–1090.
4. Kiss T, Dixon LE, Soltész A, Bányai J, Mayer M, Balla K, et al. Effects of Ambient Temperature in Association with Photoperiod on Phenology and on the Expressions of Major Plant Developmental Genes in Wheat (Triticum Aestivum L.). Plant, Cell & Environment 2017;40(8):1629–1642.
5. White JW, Hoogenboom G, Kimball BA, Wall GW. Methodologies for Simulating Impacts of Climate Change on Crop Production. Field Crops Research 2011 Dec;124(3):357–368.
6. Araus JL, Kefauver SC, Zaman-Allah M, Olsen MS, Cairns JE. Translating High-Throughput Phenotyping into Genetic Gain. Trends in Plant Science 2018;23(5):451–466.
7. Kirchgessner N, Liebisch F, Yu K, Pfeifer J, Friedli M, Hund A, et al. The ETH Field Phenotyping Platform FIP: A Cable-Suspended Multi-Sensor System. Functional Plant Biology 2017;44:154–168.
8. Kronenberg L, Yu K, Walter A, Hund A. Monitoring the Dynamics of Wheat Stem Elongation: Genotypes Differ at Critical Stages. Euphytica 2017;213(157).
9. Kronenberg L, Yates S, Boer MP, Kirchgessner N, Walter A, Hund A. Temperature Response of Wheat Affects Final Height and the Timing of Stem Elongation under Field Conditions. Journal of Experimental Botany 2020;.
10. Roth L, Hund A, Aasen H. PhenoFly Planning Tool: Flight Planning for High-Resolution Optical Remote Sensing with Unmanned Areal Systems. Plant Methods 2018;14(116).
11. Roth L, Camenzind M, Aasen H, Kronenberg L, Barendregt C, Camp KH, et al. Repeated Multiview Imaging for Estimating Seedling Tiller Counts of Wheat Genotypes Using Drones. Plant Phenomics 2020;2020(3729715).
12. Roth L, Kronenberg L, Aasen H, Walter A, Hartung J, van Eeuwijk F, et al. High-Throughput Field Phenotyping Reveals That Selection in Breeding Has Affected the Phenology and Temperature Response of Wheat in the Stem Elongation Phase. Journal of Experimental Botany 2024 Mar;75(7):2084–2099.
13. Gogna A, Schulthess AW, Röder MS, Ganal MW, Reif JC. Gabi Wheat a Panel of European Elite Lines as Central Stock for Wheat Genetic Research. Scientific Data 2022;9(538).
14. Kollers S, Rodemann B, Ling J, Korzun V, Ebmeyer E, Argillier O, et al. Whole Genome Association Mapping of Fusarium Head Blight Resistance in European Winter Wheat (Triticum Aestivum L.). PLoS ONE 2013;8(2).
15. Anderegg J, Hund A, Karisto P, Mikaberidze A. In-Field Detection and Quantification of Septoria Tritici Blotch in Diverse Wheat Germplasm Using Spectral–Temporal Features. Frontiers in Plant Science 2019;10(1355).
16. Anderegg J, Yu K, Aasen H, Walter A, Liebisch F, Hund A. Spectral Vegetation Indices to Track Senescence Dynamics in Diverse Wheat Germplasm. Frontiers in Plant Science 2020;10(1749).
17. Anderegg J, Aasen H, Perich G, Roth L, Walter A, Hund A. Temporal Trends in Canopy Temperature and Greenness Are Potential Indicators of Late-Season Drought Avoidance and Functional Stay-Green in Wheat. Field Crops Research 2021;274(108311).
18. Roth L, Fossati D, Krähenbühl P, Walter A, Hund A. Image-based Phenomic Prediction Can Provide Valuable Decision Support in Wheat Breeding. Theoretical and Applied Genetics 2023 Jun;136(7):162.
19. Roth L, Piepho HP, Hund A. Phenomics Data Processing: Extracting Dose-Response Curve Parameters from High-Resolution Temperature Courses and Repeated Field-Based Wheat Height Measurements. in silico Plants 2022;4(1).
20. Roth L, Rodríguez-Álvarez MX, van Eeuwijk F, Piepho HP, Hund A. Phenomics Data Processing: A Plot-Level Model for Repeated Measurements to Extract the Timing of Key Stages and Quantities at Defined Time Points. Field Crops Research 2021;274(108314).
21. Pérez-Valencia DM, Rodríguez-Álvarez MX, Boer MP, Kronenberg L, Hund A, Bosquet LC, et al. A Two-stage Approach for the Spatio-temporal Analysis of High-throughput Phenotyping Data. Scientific Reports 2022;12(3177).
22. Zenkl R, Timofte R, Kirchgessner N, Roth L, Hund A, Van Gool L, et al. Outdoor Plant Segmentation With Deep Learning for High-Throughput Field Phenotyping on a Diverse Wheat Dataset. Frontiers in Plant Science 2022;12(774068).
23. Tschurr F, Kirchgessner N, Hund A, Kronenberg L, Anderegg J, Walter A, et al. Frost Damage Index: The Antipode of Growing Degree Days. Plant Phenomics 2023 Sep;0(ja).
24. David E, Madec S, Sadeghi-Tehran P, Aasen H, Zheng B, Liu S, et al. Global Wheat Head Detection (GWHD) Dataset: A Large and Diverse Dataset of High-Resolution RGB-Labelled Images to Develop and Benchmark Wheat Head Detection Methods. Plant Phenomics 2020;2020(3521852).
25. Roth L, Binder M, Kirchgessner N, Tschurr F, Yates S, Hund A, et al. From Neglecting to Including Cultivar-Specific Per Se Temperature Responses: Extending the Concept of Thermal Time in Field Crops. Plant Phenomics 2024 Jun;6:0185.
26. Wilkinson MD, Dumontier M, Aalbersberg IJ, Appleton G, Axton M, Baak A, et al. The FAIR Guiding Principles for Scientific Data Management and Stewardship. Scientific Data 2016 Dec;3(160018).
27. Lhoest Q, Villanova del Moral A, Jernite Y, Thakur A, von Platen P, Patil S, et al. Datasets: A Community Library for Natural Language Processing. In: Proceedings of the 2021 Conference on Empirical Methods in Natural Language Processing: System Demonstrations Online and Punta Cana, Dominican Republic: Association for Computational Linguistics; 2021. p. 175–184. https://aclanthology.org/2021.emnlp-demo.21.
28. Papoutsoglou EA, Faria D, Arend D, Arnaud E, Athanasiadis IN, Chaves I, et al. Enabling Reusability of Plant Phenomic Datasets with MIAPPE 1.1. New Phytologist 2020;227:260–273.
29. Gogna A, Börgel AWS, Röder M, Ganal MW, Reif JC, The Genotypic Data of Elite European Cultivar Panel Comprising 358 Winter and 14 Summer Wheat Varieties Released from 1975 to 2007 at Different Marker Densities. Dryad; 2022.
30. Kempton RA. The Design and Analysis of Unreplicated Field Trials. Vorträge für Pflanzenzüchtung 1984;7:219–242.
31. Inc TM, MATLAB version: 9.13.0 (R2022b). Natick, Massachusetts, United States: The MathWorks Inc.; 2022. https://www.mathworks.com.
32. Roth L, Aasen H, Walter A, Liebisch F. Extracting Leaf Area Index Using Viewing Geometry Effects—A New Perspective on High-Resolution Unmanned Aerial System Photography. ISPRS Journal of Photogrammetry and Remote Sensing 2018;141:161–175.
33. Roth L, Barendregt C, Bétrix CA, Hund A, Walter A. High-Throughput Field Phenotyping of Soybean: Spotting an Ideotype. Remote Sensing of Environment 2022;269(112797).
34. Sun J, Shen Z, Wang Y, Bao H, Zhou X. LoFTR: Detector-free local feature matching with transformers. In: Proceedings of the IEEE/CVF conference on computer vision and pattern recognition; 2021. p. 8922–8931.
35. Bradski G. The OpenCV Library. Dr Dobb's Journal of Software Tools 2000;.

36. Rierchert M. rawpy: RAW image processing for Python, a wrapper for libraw. github 2021;.
37. Roth L, Streit B. Predicting Cover Crop Biomass by Lightweight UAS-based RGB and NIR Photography: An Applied Photogrammetric Approach. Precision Agriculture 2018;19:93–114.
38. Tschurr F, Feigenwinter I, Fischer AM, Kotlarski S. Climate Scenarios and Agricultural Indices: A Case Study for Switzerland. Atmosphere 2020;11.
39. Pask A, Pietragalla J, Mullan D, Reynolds M, editors. Physiological Breeding II: A Field Guide to Wheat Phenotyping. Mexico, D.F.: CIMMYT; 2012.
40. Lancashire PD, Bleiholder H, Van den Boot T, Langelüddeke P, Strauss R, Weber E, et al. A Uniform Decimal Code for Growth Stages of Crops and Weeds. Annals of Applied Biology 1991;119:561–601.
41. Rodríguez-Álvarez MX, Boer MP, van Eeuwijk FA, Eilers PHC. Correcting for spatial heterogeneity in plant breeding experiments with P-splines. Spatial Statistics 2017;23:52 – 71.
42. Butler D. asreml: Fits the Linear Mixed Model; 2018, www.vsni.co.uk, r package version 4.1.0.93.
43. Smith RN, Aleksic J, Butano D, Carr A, Contrino S, Hu F, et al. InterMine: A Flexible Data Warehouse System for the Integration and Analysis of Heterogeneous Biological Data. Bioinformatics (Oxford, England) 2012 Dec;28(23):3163–3165.
44. Schwender H, with a contribution of Arno Fritsch. scrime: Analysis of High-Dimensional Categorical Data Such as SNP Data; 2018, https://CRAN.R-project.org/package=scrime, r package version 1.3.5.
45. VanRaden PM. Efficient Methods to Compute Genomic Predictions. Journal of Dairy Science 2008;91(11):4414–4423.
46. Yang J, Benyamin B, McEvoy BP, Gordon S, Henders AK, Nyholt DR, et al. Common SNPs Explain a Large Proportion of the Heritability for Human Height. Nature Genetics 2010 Jul;42(7):565–569.
47. Malosetti M, Bustos-Korts D, Boer MP, van Eeuwijk FA. Predicting Responses in Multiple Environments: Issues in Relation to Genotype x Environment Interactions. Crop Science 2016;56:2210–2222.
48. Piepho HP. Prediction of and for New Environments: What's Your Model? Molecular Plant 2022;15(4):581–582.
49. Bustos-Korts D, Malosetti M, Chapman S, Biddulph B, van Eeuwijk F. Improvement of Predictive Ability by Uniform Coverage of the Target Genetic Space. G3 Genes|Genomes|Genetics 2016 Nov;6(11):3733–3747.
50. Akdemir D. STPGA: Selection of Training Populations by Genetic Algorithm; 2018, https://CRAN.R-project.org/package=STPGA, r package version 5.2.1.
51. Akdemir D, Isidro-Sánchez J. Design of Training Populations for Selective Phenotyping in Genomic Prediction. Scientific Reports 2019 Feb;9(1):1446.
52. Oakey H, Verbyla A, Pitchford W, Cullis B, Kuchel H. Joint Modeling of Additive and Non-Additive Genetic Line Effects in Single Field Trials. Theoretical and Applied Genetics 2006;113:809–819.
53. Cullis BR, Smith AB, Coombes NE. On the Design of Early Generation Variety Trials with Correlated Data. Journal of Agricultural, Biological, and Environmental Statistics 2006;11(4):381–393.
54. Jarquín D, Crossa J, Lacaze X, Du Cheyron P, Daucourt J, Lorgeou J, et al. A Reaction Norm Model for Genomic Selection Using High-Dimensional Genomic and Environmental Data. Theoretical and Applied Genetics 2014 Mar;127(3):595–607.
55. Beguería S, Vicente-Serrano SM, Reig F, Latorre B. Standardized Precipitation Evapotranspiration Index (SPEI) Revisited: Parameter Fitting, Evapotranspiration Models, Tools, Datasets and Drought Monitoring. International Journal of Climatology 2014;34(10):3001–3023.
56. Tolhurst DJ, Gaynor RC, Gardunia B, Hickey JM, Gorjanc G. Genomic Selection Using Random Regressions on Known and Latent Environmental Covariates. Theoretical and Applied Genetics 2022;(i).
