# Supplementary material for: The FIP 1.0 Data Set: Highly resolved annotated image time series of 4,000 wheat plots grown in 6 years
Source: Gigascience. 2025 Jun 11;14:giaf051. doi: 10.1093/gigascience/giaf051 (PMC12153353; doi:10.1093/gigascience/giaf051)
Supplement: giaf051_Authors_Response_To_Reviewer_Comments_original_submission [file giaf051_authors_response_to_reviewer_comments_original_submission.pdf]

Author's Response To Reviewer Comments

Dear editors of GigaScience,

Thank you for giving us the chance to perform major revisions on our data note manuscript based on the comments of two reviewers. In the last weeks, we have invested a collaborative effort into revising our manuscript as well as providing additional data and analyses to further improve its value for the community. In particular, we have reacted to the major concerns of reviewer #1 and have
clearly stated the availability of open genetic data for the majority of the presented data and the ability to have full access to all genetic data via collaborations under clearly defined conditions
addressed the MIAPPE standard and are now fully compatible with MIAPPE v1.1

In reaction to the concerns of reviewer #2 regarding quality control and consistency checks, we have substantially revised our manuscript to clarify open points.

Please find our point-by-point reply to all raised questions and issues by the reviewers as well as the revised manuscript and the manuscript with highlighted changes below.

Reviewer #1: Thank you for the submission. The dataset surely holds value for the plant breeding community

Thank you very much for your thorough review. We have substantially revised the manuscript to address your concerns. Please find indications of all revisions and reactions to your comments below.

but my major concerns are
(1) the availability of genetic data,

We agree that the dependence on non-public genetic data would restrict the value of the dataset. Yet, this is not the case: For the majority of the data presented in this data note, marker data are public. This data set allows all the uses described in the manuscript, and we used it as default for the baselines, splits, etc. (see e.g. Figure 5). Yet, we wanted to inform the reader of the data note that it is possible to extend the public dataset via collaborating with the Swiss plant breeders, which may be an interesting option for some, but absolutely no prerequisite. We have reformulated the section in the introduction to point this out more clearly:

“For the first three years (2016-2018), the GABI-WHEAT [13] panel was grown as the genotype set. From 2019-2022, a subset of the GABI panel was grown in addition to other genotypes (Figure 2, green bars). The GABI panel consists of registered genotypes from different climatic regions of Europe [14, 13]. Genetic marker data and MET data from eight year-locations for GABI-WHEAT are publicly available.

The GABI panel was largely superseded by the Swiss breeding set in 2021 (Figure 2, orange bars). This new set primarily consists of eighth-generation (F8) breeding genotypes.
Private genetic marker data are available for the Swiss breeding set. These can be shared directly through the breeders as part of a collaboration. The remaining genotypes, linked to specific projects such as INVITE, were present throughout all years but were generally only grown in a single year each (Figure 2, in purple). These genotypes currently lack available marker data.

In summary, by default the dataset contains public genotypic data from ~300 genotypes over six years, allowing all the uses described in this dataset note. Through collaborations, this set can be expanded to ~800 genotypes.”

(2) non-conformity to MIAPPE standards (https://www.miappe.org/).

We agree that MIAPPE is an important logical data model to ensure a minimum set of metadata describing phenotyping experiments. We have used MIAPPE as reference and checklist for the design of our own internal database that holds our experimental data and metadata (https://kp.ethz.ch/infrastructure/cropydb.html). This approach corresponds to the aims of MIAPPE (https://www.miappe.org/: “MIAPPE provides a specification including a checklist and a data model of metadata required to adequately describe plant phenotyping experiments.”)

Consequently, we consider the exports of the database in the shared data repositories accompanying this data note as fully conform to MIAPPE standards.

To further facilitate the re-use of our MIAPPE-compatible dataset, we have now added the necessary MIAPPE tsv files to both the research collection and Huggingface dataset repository. In addition, we also added the script to generate the MIAPPE files for the biological material, observation unit and observed variables to the fip1_dataset repository under miappe.py.

We have added a corresponding hint to the “Interoperable” declaration:
“Interoperable: The use of the open-source Hugging Face datasets [27] package makes it easy to use and export to different formats and is fully MIAPPE v1.1 [28] conform.”

These restrict value of the otherwise excellent publication. I would welcome a submission addressing these major points. In addition, I have some minor points for specific sections. Please use the strings in quotation marks ("") to locate the specific sections.
1. Context
* Change of Equipment: Please indicate how the change of equipment from TLS to drone affects data interoperability.

We have indeed demonstrated in previous publications that those two measurements are in high accordance with each other. We have revised the sentence accordingly:

“Up to three times a week, RGB images of all experimental units (so-called `plots') were collected, and plant heights were measured simultaneously using either the TLS (2016, 2017) [8, 9] or drones (2018--2022) [10, 11, 12], two methods of height measurement that have been in good accordance with one another (R2: 0.99 [12]).”

Furthermore, we added supplementary information to the materials and methods section:

“The accuracy of TLS and drone measurements to approximate manual plant height measurements were demonstrated in the respective publications (TLS [8]: R2 : 0.99, drone [37]: R2 : 0.96). The two methods are in good accordance with one another (R2 : 0.99, intercept: 0.057 m, slope: 1.0 [12]).”

* "Figure 2, gray bars": Kindly update Figure 2 to clarify the representation of the gray bars.

Sorry for that oversights in the text, we confused “grey bar” with “orange bar”:

“The GABI-WHEAT panel was largely superseded by the Swiss breeding set in 2021 (Figure 2, orange bars)”

* "Heads were annotated": Does this mean that not all relevant images were annotated? If so, please modify the title to avoid confusion.

Image annotation may be done on different levels, from labels of images to bounding boxes or polygons. All of these data are considered “annotations”. The time series in this data set are annotated with multiple labels per image (canopy cover, number of wheat heads, plant height, senescence) and multiple labels per time series (yield, protein content, heading date, final height). Hence, we have chosen the title “highly

resolved annotated image time series", which we found to adequately describe the characteristics of the dataset.

To point out not only the potential but also the limitations of the data set, we have added a discussion to the section "Re-use Potential" and renamed it to "Re-use Potential and Limitations":

"While the data set opens up the possibility of analysing HTFP data to a wide audience, it also has its inherent limitations that should be taken into account if working with it:
The immobility of the FIP restricts the data set to only one location.
Field-based data collection introduces various sources of errors that one must consider in analysis (see e.g. [20] for a discussion).
Annotation at the image level only requires further annotation effort if semantic segmentation or object detection methods are targeted."

If you do not agree with this argumentation and still have concerns about confusion on the readers side, we are of course open for suggestions on how to more accurately describe this type of annotations in the title.

* Description of FAIR: Please revise this section. Both links listed under "Findable" and "Accessible" are eligible for these tags. Please modify "Interoperability" with reference to the publication listed in the "Re-use Potential."

We have added the most important publications from the re-use potential to the interoperability section.

"Given the shared genotypes the data set can be used to enhance the data by Gogna et al. [13] by 6 environments, to a total of 14. The data set expands on existing sub-sets of the data already released that can be used as baseline approaches such as
[8, 9, 19, 20, 21, 12, 25, 15, 16, 17]."

2. Reference measurements
* "Senescence was": Was this measurement done for all relevant images? Please include this information.

The period and years of measurement were indeed missing for the senescence ratings, thank you for pointing this out. We revised the sentence:

"Senescence was assessed visually in 2016, 2017 and 2018 from approximately 20 days after flowering to full senescence for the central plot area canopy, following guidelines provided by Pask et al. [39]. Plot senescence was scored according to Anderegg et al. [16] based on the portion of green leaf area on a scale from 0 to 10, equivalent to 0 to 100 %."

* "Adjusted genotype means with year calculation": Please add variance decomposition data for traits.

Thank you for this suggestion. We extracted the estimated variance components for the intermediate and target traits and displayed the results in a new table, Table 4. When rerunning the mixed models, we took the opportunity to further increase the consistency with the low-level trait BLUEs and heritability estimates that were performed in SpATS, and changed estimations for intermediate and target traits also to SpATS. Heritabilities changed slightly, but not significantly (Table 3).

When doing the recalculations, we realized that the spatial variances for yield for 2019 were exceptionally high in comparison to other years. Further investigations revealed that not all plots in 2019 had the same size: While some were 1 m x 1.5 m size as all plots of all other years, 144 plots had size 5 m x 1.5 (yield plots). The yield values for those yield plots were on average 1.5 times lower than the ones for the small plots, despite being normalized to area. Hence, we concluded that the small plot size has led to a systematic overestimation of yield, which is not relevant if staying in sets with the same plot size, but becomes relevant for 2019 where plot sizes are heterogeneous.

We have added corresponding parts to the manuscript that explain how the conversion factor between the

two sizes was evaluated using common genotypes, and how yield for small plot sizes was compensated afterwards, leading to a new trait “Grain yield (adjusted)”. We also have re-run all baseline approaches (genomic predictions) and further clarified the description of the linear mixed models used and fall-back versions in case of failed convergence.

Changes:
Table 4: Variance decomposition results
Table 3: Heritability for Grain yield (adjusted) 2019 added
Table 7: Genomic prediction results for Grain yield (adjusted) added
Materials and Methods, Adjusted genotype means within year calculation: Correction of definition of fixed and random factors, fall-back options for failed convergence introduced
Materials and Methods, Target Traits: Description added how yield for small plot sizes was compensated:

“In 2019, plot sizes varied between experiments, which influences yield measurements.
While large plot sizes deliver absolute estimates comparable with METs, in our experience, small plot sizes tend to overestimate yield per area. To compensate for this effect, adjusted genotype means (see next Section) of twelve common genotypes between the experiments with large and small plots were used to calculate a conversion factor. A linear regression with intercept zero estimated a conversion factor of 1.50 from large to small plots (R2 of 0.47). This conversion factor was used to transform all yield measurement estimates of small plots to the range of large plots, resulting in a new trait `Grain yield (adjusted)'.”

3. Compilation as Data set
* "pure GABI-WHEAT set for the extended set": Please revise this sentence for clarity.

We have corrected and clarified the sentences:
“The splits were calculated separately for the pure GABI-WHEAT set and for the extended set. The extended set additionally includes F8 generation genotypes with private marker data.”

4. Heritabilities of intermediate and target traits
* "y of the public marker" - Please revise the sentence for clarity.

We have corrected the sentence:

“The quality of the public marker data set for the GABI-WHEAT panel was demonstrated in Gogna et al. by means of testing for genomic prediction ability [13].”

5. Genomic prediction ability of unseen multi-environment trial
* Is the CDC data part of the data publication? Please add this information.

We have clarified this point:

“These additional environmental covariate data and MET data are available in the data repository for convenience, but not part of the core data set (see folder `MET_repository_clone').”

6. Example 1 to 6
* Please revise all code for consistency and updated results. Also, include the necessary packages required to run the code.

Given the changes to the dataset we adjusted the code examples and added a clarifying sentence that only the datasets package is required.

To run the following examples the Huggingface datasets [27] library
is required. The examples were run using version 3.3.2.

7. Availability of Source code and Requirement Please create connectivity between repositories and add descriptive README files outlining their usage. Additionally, please provide instructions on how individual

repositories may be used.

We have updated the READMEs of the three repositories as well as added explanatory text to the "Availability of Source Code and Requirements" section:

"The code to recreate the derived data and the dataset is publicly available in three repositories, namely the FIP 1.0 Data Set - Traits, fip1-alignment, and fip1-dataset repositories.

The complete process to create the dataset involves extracting trait data from the raw data using the FIP 1.0 Data Set - Traits repository, then aligning the image time-series using the fip1-alignment repository, and finally aggregating the derived data into the final dataset using the fip1-dataset repository.

In addition, the dataset can be recreated using the fip1-dataset repository from the derived data that is freely available in the ETH research collection."

I appreciate your attention to these points and believe that addressing them will strengthen your manuscript.

Reviewer #2: The manuscript presents a comprehensive dataset spanning six years, encompassing data from eight key growth stages of wheat, along with corresponding phenotypic data. The construction of such a comprehensive dataset is highly valuable. However, from the perspective of dataset construction itself, quality control and consistency checks require further refinement.

Thank you very much for your thorough review. We have substantially revised the manuscript to address your concerns. Please find indications of all revisions and reactions to your comments below.

Specific issues are as follows:
1. How is the consistency check of parameters such as canopy cover and plant height at the eight key growth stages ensured? Especially for parameters like phenological stages and senescence assessment, which are determined through visual evaluation and thus susceptible to subjective influences, quality control and consistency check become particularly crucial. It is recommended to supplement relevant content for detailed explanation.

We have added supplementary information to the quality of all measured and extracted low-level and intermediate traits:

Plant height:
"The accuracy of TLS and drone measurements to approximate manual plant height measurements were demonstrated in the respective publications (TLS [8]: R2 : 0.99, drone [37]: R2 : 0.96). The two methods are in good accordance with one another (R2 : 0.99, intercept: 0.057 m, slope: 1.0 [12])."

Canopy cover:
"From images, canopy cover was extracted using a deep learning model described in Zenkl et al. [22], a model that reached a pixel accuracy of 0.945 on a FIP test set."

Wheat head count:
"As the wheat head detection method, the winning model of the global wheat head challenge [24] (https://github.com/ksnxr/GWC_solution) was used, a model that achieved an average domain accuracy of 0.7 on a test set that included FIP images."

Senescence:
"To avoid bias, manual ratings were performed by the same expert in all three years."

Heading date:
“To ensure consistent ratings over time, heading date ratings were started at approximately BBCH 55 and continue to BBCH 61, with two to three rating events per week.”

Final height:
“Final height was extracted from TLS or SfM plant height measurements using the QMER method described in Roth et al. [20], which has a reported accuracy of close to 1.0 on simulated data.”

2. For all images (151,150 out of 158,891 images), the success rate of alignment and within-field detection exceeded 95%. Does this mean that the final RGB sequence image dataset consists of 151,150 images?

Yes, this is the case if one uses the aligned sequences, but not if using unaligned images. We have revised this section and added a table that shows the number of successfully aligned images for each year. As we updated our the data set, numbers changed slightly.

“In total, the alignment and inner plot detection was successful for more than 95% of all images. The number of successfully aligned images per year can be seen in Table 2. Both the 160,772 original images and their corresponding 153,022 inner plot cutouts are made
available as part of this data set.”

3. Regarding plant height measurement, the text mentions that "TLS (2016, 2017) or UAV (2018 to 2022) was used to measure plant height." Given the potential differences in height measurements obtained from these two methods, how were these differences addressed in the manuscript?

We have indeed demonstrated in previous publications that those two measurements are in high accordance with each other. We have revised the sentence accordingly:

“Up to three times a week, RGB images of all experimental units (so-called `plots') were collected, and plant heights were measured simultaneously using either the TLS (2016, 2017) [8, 9] or drones (2018--2022) [10, 11, 12], two methods of height measurement that have been in good accordance with one another (R2: 0.99, Offset: 0.057 m [12]).”

4. Does this dataset cater to different tasks and include annotated data? If so, it is recommended to specify the concrete annotation methods and data.

The data set does not include image annotations, e.g. segmentation masks. Only scalar values for all traits are included. We have added two clarifying sentences in the measurements section.

“Trait measurements may have been made at times different from the image captures.
All measurements are scalars that correspond to a single measurement at a given time point during the season.”

5. If possible, it is recommended to provide a summary table that specifies the different types of data contained in the dataset along with their respective quantities, facilitating readers' comprehensive understanding of the dataset.

We have added a table for the images / aligned images as well as an additional table for the low-level traits. Both these tables contain the minimum, maximum and mean number of measurements for the aligned images / trait for each year (Table 3 and 4).

6. What are the potential limitations of this dataset? It is recommended to point them out.

We have enhanced the “Re-use Potential” section to a “Re-use Potential and Limitations” section:

“While the data set opens up the possibility of analysing HTFP data to a wide audience, it also has its inherent limitations that should be taken into account if working with it:

The immobility of the FIP restricts the data set to only one location.
Field-based data collection introduces various sources of errors that one must consider in analysis (see e.g. [20] for a discussion).
Yield measurements in the FIP and hence this data set are more prone to error than in METs.
Annotation at the image level only requires further annotation effort if semantic segmentation or object detection methods are targeted."
While the aligned image time series provides extensive opportunities to analyze growth dynamics, this kind of highly preprocessed image data is yet rare and therefore interoperability with other data sources is limited.

Editorial Manager®

Cookie Preference Center

We use cookies which are necessary to make our site work. We may also use additional cookies to analyze, improve and personalize our content and your digital experience. For more information, see our Cookie Policy.

You may choose not to allow some types of cookies. However, blocking some types may impact your experience of our site and the services we are able to offer. See the different category headings below to find out more or change your settings.

Allow all

Manage Consent Preferences

Strictly Necessary Cookies

Always active

These cookies are necessary for the website to function and cannot be switched off in our systems. They are usually only set in response to actions made by you which amount to a request for services, such as setting your privacy preferences, logging in or filling in forms. You can set your browser to block or alert you about these cookies, but some parts of the site will not then work. These cookies do not store any personally identifiable information.

Functional Cookies

These cookies enable the website to provide enhanced functionality and personalisation. They may be set by us or by third party providers whose services we have added to our pages. If you do not allow these cookies then some or all of these services may not function properly.

Performance Cookies

These cookies allow us to count visits and traffic sources so we can measure and improve the performance of our site. They help us to know which pages are the most and least popular and see how visitors move around the site.

Targeting Cookies

These cookies may be set through our site by our advertising partners. They may be used by those companies to build a profile of your interests and show you relevant adverts on other sites. If you do not allow these cookies, you will experience less targeted advertising.

Cookie List

Clear

Apply Cancel

Consent Leg.Interest

Confirm my choices

Powered by onetrus
