# Supplementary material for: The FIP 1.0 Data Set: Highly resolved annotated image time series of 4,000 wheat plots grown in 6 years
Source: Gigascience. 2025 Jun 11;14:giaf051. doi: 10.1093/gigascience/giaf051 (PMC12153353; doi:10.1093/gigascience/giaf051)
Supplement: giaf051_Reviewer_1_Report_original_submission — Abhishek Gogna, M.Sc. -- 12/2/2024 [file giaf051_reviewer_1_report_original_submission.pdf]

Reviewer Report

Title: The FIP 1.0 Data Set: Highly Resolved Annotated Image Time Series of 4,000 Wheat Plots Grown in Six Years

Version: Original Submission **Date:** 12/2/2024

Reviewer name: Abhishek Gogna, M.Sc.

Reviewer Comments to Author:

Thank you for the submission. The dataset surely holds value for the plant breeding community but my major concerns are (1) the availability of genetic data, (2) non-conformity to MIAPPE standards (https://www.miappe.org/). These restrict value of the otherwise excellent publication. I would welcome a submission addressing these major points. In addition, I have some minor points for specific sections. Please use the strings in quotation marks ("") to locate the specific sections.1. Context* Change of Equipment: Please indicate how the change of equipment from TLS to drone affects data interoperability.* "Figure 2, gray bars": Kindly update Figure 2 to clarify the representation of the gray bars.* "Heads were annotated": Does this mean that not all relevant images were annotated? If so, please modify the title to avoid confusion.* Description of FAIR: Please revise this section. Both links listed under "Findable" and "Accessible" are eligible for these tags. Please modify "Interoperability" with reference to the publication listed in the "Re-use Potential."2. Reference measurements* "Senescence was": Was this measurement done for all relevant images? Please include this information.* "Adjusted genotype means with year calculation": Please add variance decomposition data for traits.3. Compilation as Data set* "pure GABI-WHEAT set for the extended set": Please revise this sentence for clarity.4. Heritabilities of intermediate and target traits* "y of the public marker" - Please revise the sentence for clarity.5. Genomic prediction ability of unseen multi-environment trial* Is the CDC data part of the data publication? Please add this information.6. Example 1 to 6* Please revise all code for consistency and updated results. Also, include the necessary packages required to run the code.7. Availability of Source code and RequirementPlease create connectivity between repositories and add descriptive README files outlining their usage. Additionally, please provide instructions on how individual repositories may be used.I appreciate your attention to these points and believe that addressing them will strengthen your manuscript.

Level of Interest

Please indicate how interesting you found the manuscript: Choose an item.

Quality of Written English

Please indicate the quality of language in the manuscript: Choose an item.

Declaration of Competing Interests

Please complete a declaration of competing interests, considering the following questions:

- Have you in the past five years received reimbursements, fees, funding, or salary from an organisation that may in any way gain or lose financially from the publication of this manuscript, either now or in the future?
- Do you hold any stocks or shares in an organisation that may in any way gain or lose financially from the publication of this manuscript, either now or in the future?
- Do you hold or are you currently applying for any patents relating to the content of the manuscript?
- Have you received reimbursements, fees, funding, or salary from an organization that holds or has applied for patents relating to the content of the manuscript?
- Do you have any other financial competing interests?
- Do you have any non-financial competing interests in relation to this paper?

If you can answer no to all of the above, write 'I declare that I have no competing interests' below. If your reply is yes to any, please give details below.

I declare that I have no competing interests

I agree to the open peer review policy of the journal. I understand that my name will be included on my report to the authors and, if the manuscript is accepted for publication, my named report including any attachments I upload will be posted on the website along with the authors' responses. I agree for my report to be made available under an Open Access Creative Commons CC-BY license (http://creativecommons.org/licenses/by/4.0/). I understand that any comments which I do not wish to be included in my named report can be included as confidential comments to the editors, which will not be published.

Choose an item.

To further support our reviewers, we have joined with Publons, where you can gain additional credit to further highlight your hard work (see: https://publons.com/journal/530/gigascience). On publication of this paper, your review will be automatically added to Publons, you can then choose whether or not to claim your Publons credit. I understand this statement.

Yes Choose an item.
