# Supplementary material for: The FIP 1.0 Data Set: Highly resolved annotated image time series of 4,000 wheat plots grown in 6 years
Source: Gigascience. 2025 Jun 11;14:giaf051. doi: 10.1093/gigascience/giaf051 (PMC12153353; doi:10.1093/gigascience/giaf051)
Supplement: giaf051_Reviewer_2_Report_original_submission — Wanneng Yang -- 12/16/2024 [file giaf051_reviewer_2_report_original_submission.pdf]

Reviewer Report

Title: The FIP 1.0 Data Set: Highly Resolved Annotated Image Time Series of 4,000 Wheat Plots Grown in Six Years

Version: Original Submission **Date:** 12/16/2024

Reviewer name: Wanneng Yang

Reviewer Comments to Author:

The manuscript presents a comprehensive dataset spanning six years, encompassing data from eight key growth stages of wheat, along with corresponding phenotypic data. The construction of such a comprehensive dataset is highly valuable. However, from the perspective of dataset construction itself, quality control and consistency checks require further refinement. Specific issues are as follows:1. How is the consistency check of parameters such as canopy cover and plant height at the eight key growth stages ensured? Especially for parameters like phenological stages and senescence assessment, which are determined through visual evaluation and thus susceptible to subjective influences, quality control and consistency check become particularly crucial. It is recommended to supplement relevant content for detailed explanation.2. For all images (151,150 out of 158,891 images), the success rate of alignment and within-field detection exceeded 95%. Does this mean that the final RGB sequence image dataset consists of 151,150 images?3. Regarding plant height measurement, the text mentions that "TLS (2016, 2017) or UAV (2018 to 2022) was used to measure plant height." Given the potential differences in height measurements obtained from these two methods, how were these differences addressed in the manuscript?4. Does this dataset cater to different tasks and include annotated data? If so, it is recommended to specify the concrete annotation methods and data.5. If possible, it is recommended to provide a summary table that specifies the different types of data contained in the dataset along with their respective quantities, facilitating readers' comprehensive understanding of the dataset.6. What are the potential limitations of this dataset? It is recommended to point them out.

Level of Interest

Please indicate how interesting you found the manuscript: Choose an item.

Quality of Written English

Please indicate the quality of language in the manuscript: Choose an item.

Declaration of Competing Interests

Please complete a declaration of competing interests, considering the following questions:

- Have you in the past five years received reimbursements, fees, funding, or salary from an organisation that may in any way gain or lose financially from the publication of this manuscript, either now or in the future?
- Do you hold any stocks or shares in an organisation that may in any way gain or lose financially from the publication of this manuscript, either now or in the future?
- Do you hold or are you currently applying for any patents relating to the content of the manuscript?
- Have you received reimbursements, fees, funding, or salary from an organization that holds or has applied for patents relating to the content of the manuscript?
- Do you have any other financial competing interests?
- Do you have any non-financial competing interests in relation to this paper?

If you can answer no to all of the above, write 'I declare that I have no competing interests' below. If your reply is yes to any, please give details below.

I declare that I have no competing interests

I agree to the open peer review policy of the journal. I understand that my name will be included on my report to the authors and, if the manuscript is accepted for publication, my named report including any attachments I upload will be posted on the website along with the authors' responses. I agree for my report to be made available under an Open Access Creative Commons CC-BY license (http://creativecommons.org/licenses/by/4.0/). I understand that any comments which I do not wish to be included in my named report can be included as confidential comments to the editors, which will not be published.

Choose an item.

To further support our reviewers, we have joined with Publons, where you can gain additional credit to further highlight your hard work (see: https://publons.com/journal/530/gigascience). On publication of this paper, your review will be automatically added to Publons, you can then choose whether or not to claim your Publons credit. I understand this statement.

Yes Choose an item.
